# Catalytic Triad-Inspired Nanozyme Catalysts for Ester Hydrolysis in Organic Solvent Mixtures

**DOI:** 10.1021/acscatal.5c09181

**Published:** 2026-03-27

**Authors:** Hoya Ihara, Carlos A. Huang-Zhu, Tianwei Yan, Matthew D. Edgar, Siddarth H. Krishna, Reid C. Van Lehn, James A. Dumesic, George W. Huber

**Affiliations:** † Department of Chemical and Biological Engineering, 5228 University of Wisconsin-Madison, Madison, Wisconsin 53706, United States; ‡ Department of Chemistry, University of Wisconsin-Madison, Madison, Wisconsin 53706, United States

**Keywords:** Ester hydrolysis, Nanozyme, Molecular dynamics, Reaction kinetics analysis, Self-assembled monolayer

## Abstract

We synthesized a series of molecular nanozyme catalysts containing functional groups (imidazole, carboxylic acid, and hydroxyl groups) inspired by the catalytic triad found in natural serine hydrolases. Different structural features were incorporated using two distinct synthesis routes to investigate the influence of interactions beyond the active site on catalytic activity in designing molecular nanozymes. Molecular dynamics simulations suggest that nanozyme activity is affected by structural features that influence nanozyme hydrophilicity and the organization of the local solvent environment. The most active nanozyme showed activity comparable to the enzyme α-chymotrypsin for the hydrolysis of a model ester (4-nitrophenyl 4-hydroxybenzoate) in a 95:5 (v/v%) water/acetonitrile mixture at ambient temperature. Increasing the temperature and organic solvent content decreases the activity of α-chymotrypsin while enhancing the activity of the nanozymes. The nanozymes can be immobilized on supported metal nanoparticles using a dithiol self-assembled monolayer, which facilitates their removal from the postreaction solution. These results demonstrate the potential in creating solvent-tolerant bioinspired catalysts, thereby combining the advantages of biocatalysts and chemical catalysts as next-generation industrial catalysts.

## Graphical abstract

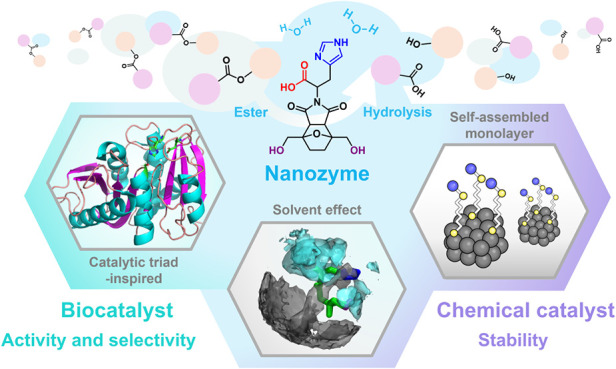

## 1 Introduction

Enzymes have high activity and selectivity for chemical reactions and are effective catalysts in industrial processes. Over the past several decades, the global industrial enzyme market has grown, reaching a value of US $7 billion by 2021, with hydrolytic enzymes accounting for the majority of those currently in use.^1,2^ However, minor changes in temperature, salt concentrations, pH, solvents, and the presence of other enzymes may disrupt protein folding, resulting in a reduction in catalytic efficiency.^3,4^ Accordingly, enzyme catalysts are often restricted to a narrow range of reaction conditions, such as operation at moderate temperatures in predominantly aqueous solutions. In general, the use of enzymes as biocatalysts in nonaqueous media would present significant advantages, including enhanced solubility of hydrophobic substrates, suppression of water-dependent side reactions, and reduced microbial contamination; however, enzyme inactivation at low organic solvent levels remains a key challenge for maintaining activity and stability.^3,5–8^ Furthermore, industrial enzymes are typically single-use, as spent enzymes are difficult to recover. Enzyme immobilization would allow for reuse, but can result in decreased activity or selectivity due to disruption of protein folding caused by the interaction with supports. These challenges limit the operational parameters and downstream processing of industrial enzymes, and often increase the production costs of final products.^9^ For ester hydrolysis, most ester substrates are intrinsically hydrophobic and require the presence of organic solvents to enhance their solubility. Consequently, hydrolytic reactions are typically conducted in mixtures of water and water-miscible organic solvents.^7,8^ Within the hydrolase class, serine hydrolases are active for ester hydrolysis and several of them are reported to remain active and stable in various organic solvents, particularly nonpolar solvents such as hexane and toluene. However, activity and stability of these enzymes decrease in the presence of water-miscible polar solvents, which can disrupt the hydration layer and induce the conformational change of active site.^10–12^ Bioinspired catalysts that integrate enzyme-like catalytic performance with high stability and the ability to operate under a broader range of reaction conditions are essential for next-generation industrial catalysts.

The high activity and selectivity of enzymes stem from active sites that have specific geometric configurations of amino acids with interactions beyond the active site such as hydrogen bonding, dispersion force, solvent effect, and other noncovalent interactions. Incorporating these features requires spatial control of functional groups.^13,14^ Initial efforts to develop functional alternatives to natural enzymes or nanozymes focused on homogeneous systems using cyclodextrins, metal complexes, and supramolecular assemblies, later evolving into heterogeneous systems with polymer and dendrimer scaffolds.^15–17^ Since the early 2000s, carbon-based and metal–organic framework (MOF)-based nanozymes have been developed^18–20^ and in the 2020s, organic nanozymes incorporating amino acid residue-like organic components emerged, further expanding functional diversity.^21–25^ Despite these advances, most nanozymes in the literature rely on metal-based active sites that mimic metalloenzyme active sites, with few organic material-based active sites developed to incorporate multiple amino acid residues present in enzyme active sites.

Here, we report the synthesis and catalytic performance of organic molecular nanozymes that incorporate functional groups inspired from those found in the active site of serine hydrolases. Serine hydrolases are active for ester hydrolysis and applied in diverse industries ranging from detergent and food to biomass conversion and fine chemical synthesis industries.^1,2,26^ Among these enzymes, polyethylene terephthalate hydrolase (PETase) has gathered significant attention due to its potential application in plastic recycling as an eco-friendly method.^27^ Figure 1A shows the polyethylene terephthalate hydrolase (PETase) from *Piscinibacter sakaiensis*.^28^ Most serine hydrolases possess a catalytic triad, a set of three coordinated functional groups typically consisting of the hydroxyl group of serine (SER), the imidazole ring of histidine (HIS), and the carboxylic acid group of aspartic acid (ASP) or glutamic acid (GA). These three functional groups work cooperatively as a charge-relay system to hydrolyze ester bonds through a two-step catalytic mechanism consisting of acylation and deacylation steps.^27–32^ The acylation step is initiated by a nucleophilic attack from the hydroxyl group of SER, which is activated via proton transfer interactions with HIS and GA. The subsequent deacylation step proceeds through the hydrolysis of the acyl-enzyme intermediate. In addition to substrate interactions with the active site catalytic triad, interactions such as hydrogen bonding with the oxyanion hole and π–π interactions with the aromatic rings of tryptophans play essential roles in enzymatic hydrolysis by PETase.^29–32^ In general, the active site is a hydrophilic domain in the hydrophobic enzyme structure.

**Figure 1:**
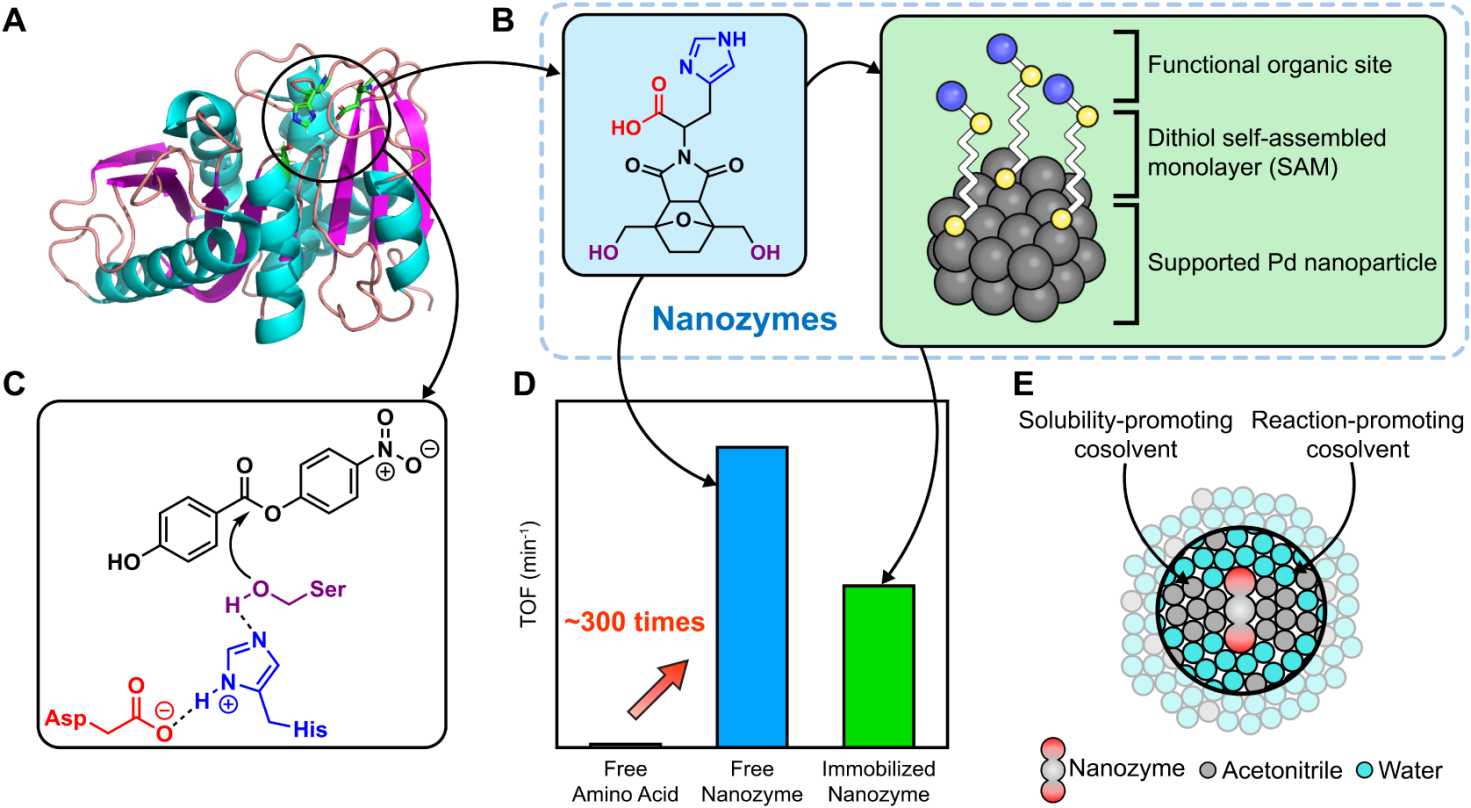
Conceptual representation of nanozyme synthesis and catalytic activity. (A) PETase from *Piscinibacter sakaiensis* [PDB ID: 6EQE] (B) Nanozyme featuring a synthetic catalytic triad inspired by PETase and its heterogenization via a self-assembled monolayer (SAM) of 1,6-hexanedithiol on Pd/SiO_2_. (C) Overview of model ester 4NP4HB hydrolysis by a catalytic triad initiated by a charge-relay system involving a carboxylate (red), imidazole (blue), and hydroxyl (purple) group. Similar functional groups were incorporated into nanozymes, with functional groups following the same color scheme. (D) Schematic comparison of catalytic activity of a free amino acid (histidine), a free nanozyme, and an immobilized nanozyme. (E) Schematic representation depicting how solvent effects dictate local chemical environments that control catalytic reactions (i.e., acetonitrile-rich regions that increase solute concentrations near the catalyst and water-rich regions that promote hydrolysis).

We synthesized nanozymes incorporating different functional groups from catalytic triads and different structural features to investigate the influence of beyond active site interactions on the catalytic activity for ester hydrolysis. Figure 1B shows the most active nanozyme, along with its immobilized form, which has carboxylate, imidazole, and hydroxyl groups inspired from the PETase catalytic triad. The nanozyme can be immobilized on a supported Pd nanoparticle using self-assembled monolayers (SAMs) (Figure 1B). We use the model substrate 4-nitrophenyl 4-hydroxybenzoate (4NP4HB) hydrolysis to compare the activity of nanozymes as shown in Figure 1C. The highest activity nanozyme has 300 times higher activity for 4NP4HB hydrolysis than free amino acid, histidine (Figure 1D). Figure 1E illustrates how nanozyme properties, including moieties other than catalytic triad, control the local solvent environment around the nanozyme to increase activity. Different structural features of nanozymes influence the clustering of reaction-promoting water molecules and solubility-promoting acetonitrile molecules around the nanozymes. Our nanozymes show comparable activity compared to a commercial enzyme (α-chymotrypsin) under standard enzymatic reaction conditions (25–40 °C in <5% v/v acetonitrile in water)^33,34^ and demonstrate enhanced activity in a solvent system containing high organic content (50% v/v acetonitrile) at elevated temperature (50 °C). Furthermore, the nanozymes can be immobilized on a three-dimensional scaffold without structural modification, for ease of separation from the reaction medium. Our approach differs from previous approaches by integrating the advantages of biocatalysts which are active and selective under mild conditions with those of chemical catalysts which provide high chemical and thermal stability. Advantages of our new approach are enhanced conversion efficiency for hydrophobic ester substrates, reduced risk of microbial contamination, and the potential for catalyst recycling.

## 2 Results and Discussion

### 2.1 Synthesis and Immobilization of Nanozymes

Figure 2 shows our approach to synthesize nanozymes (Figure 2A) and immobilize them on supported Pd nanoparticles (Figure 2B). Chemical structures and models of the nanozymes, substrate, and cosolvents are shown in Figure 3, and the abbreviations used in the nomenclature of the nanozymes are summarized in Table 1. Each component of a nanozyme name is derived from a precursor or functional group incorporated into its structure. The complete synthetic routes and NMR analyses for all nanozymes are shown in Figures S1–S4. Synthesis of the nanozyme typically starts with a HIS moiety protected with Boc and tosyl groups. The Boc protecting group is selectively removed first using methanesulfonic acid (MSA), thereby activating the amine group for subsequent reaction. The imidazole-protected HIS is then anchored to maleimide through a substitution reaction with *N*-(methoxycarbonyl)­maleimide.^35,36^ The correlations between HIS and maleimide signals, suggesting successful anchoring, are shown in the 2D HC HMBC NMR spectrum (Figure S3B). Tosyl protection on the imidazole prevents reaction with the maleimide double bond and facilitates efficient ring closure of the intermediate. The third functional moiety, the hydroxyl group, is attached via one of two distinct reaction pathways: a Diels–Alder reaction or thiol–Michael addition. Nanozymes synthesized via the Diels–Alder reaction incorporate a bulky adduct ring adjacent to the maleimide ring, and the double bond of the adduct ring is selectively hydrogenated to prevent the retro-Diels–Alder reaction.^37^ The tosyl protection is removed at the end for both reaction pathways. Previous studies on ester hydrolysis enzymes have demonstrated that the spatial arrangement of catalytic triad functional groups is essential for effective catalysis.^29–31^ The two different synthetic approaches employed in this paper are thus expected to produce nanozymes with different spatial arrangements of these functional groups and to introduce structural variations beyond the active site to assess corresponding influences on nanozyme activity.

**Table 1:**
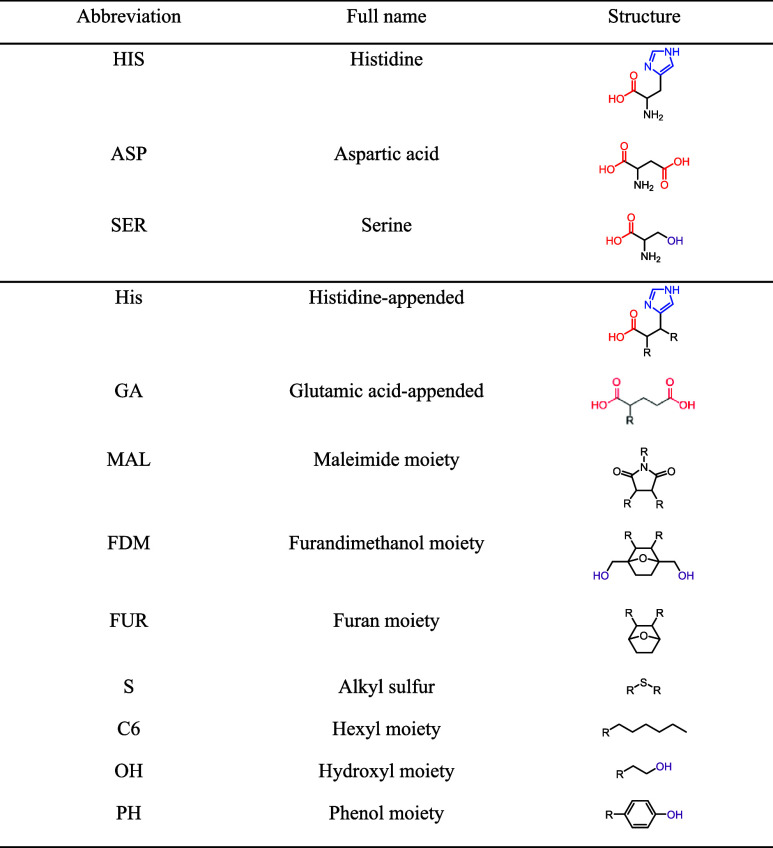
List of Abbreviations Used for Amino Acids and the Nomenclature of Nanozymes

**Figure 2:**
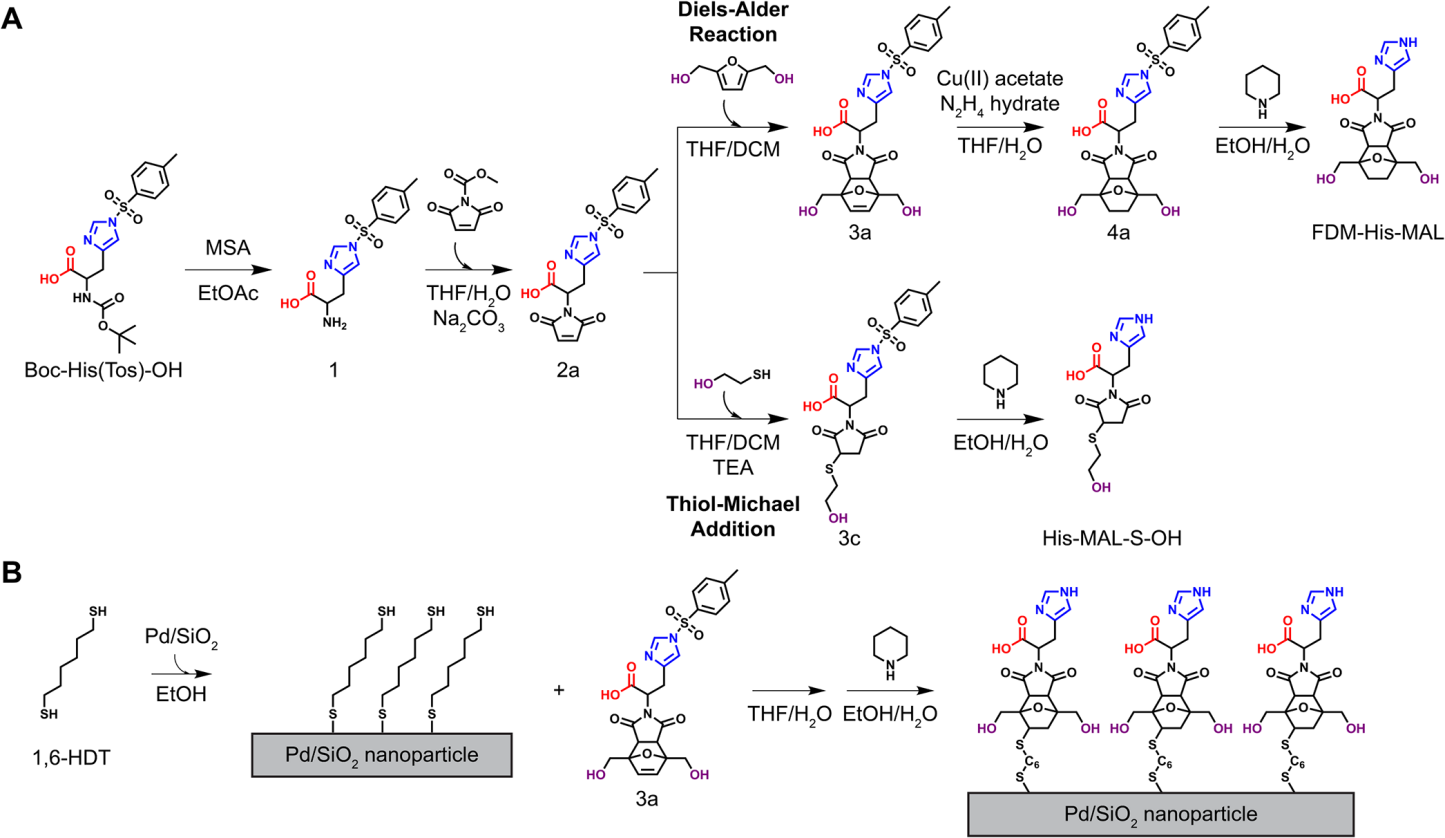
Synthesis of nanozymes with functional groups inspired by the PETase catalytic triad. (A) Reaction pathways for the synthesis of nanozymes via Diels–Alder reaction and via thiol–Michael addition. (B) Reaction pathways for the preparation of 1,6-hexanedithiol SAM on Pd/SiO_2_ and the functionalization of SAM via thiol–ene reaction.

**Figure 3:**
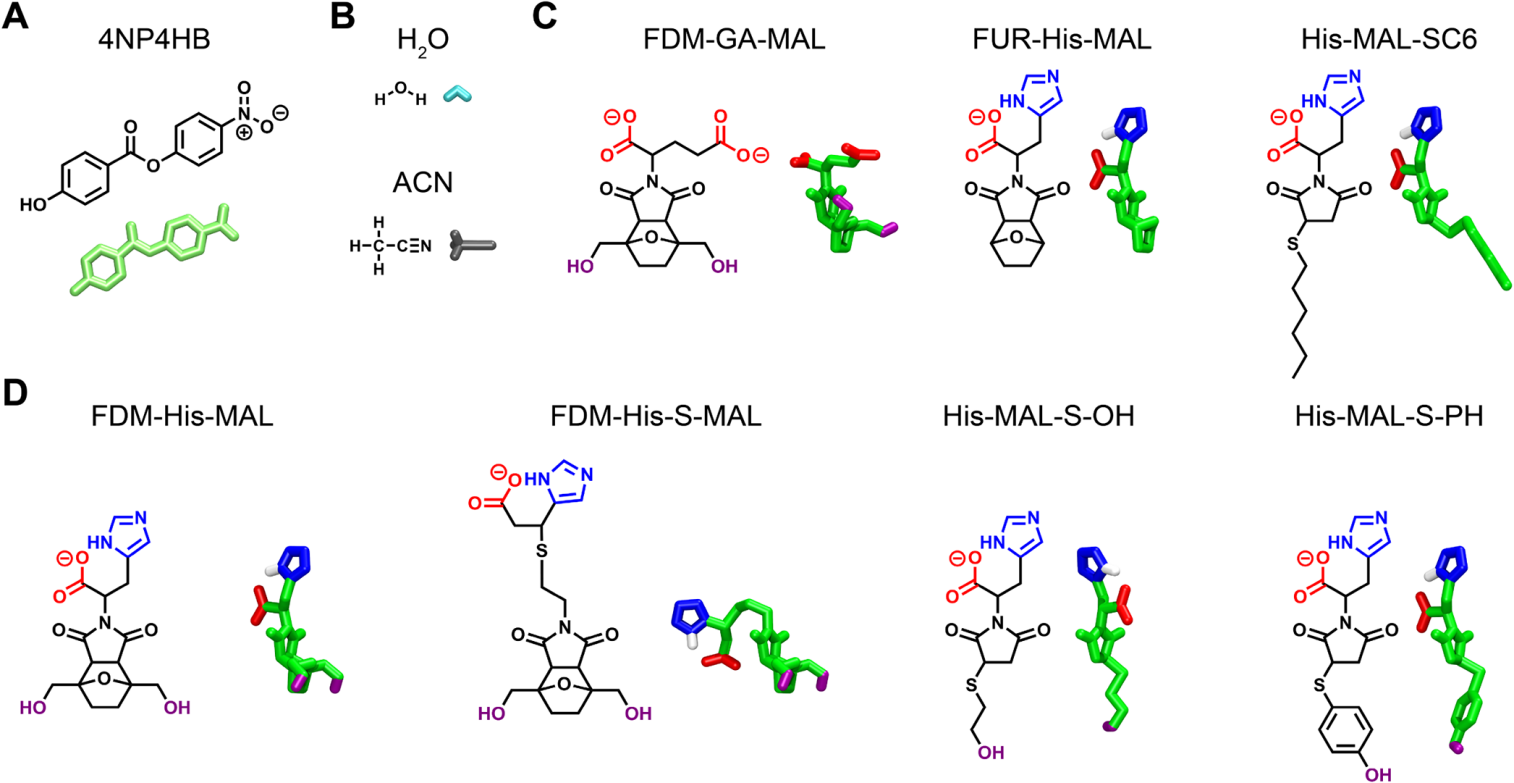
Chemical structures and all-atom models of the synthesized nanozymes, model ester, and cosolvents used in this study. (A) Model ester, (B) cosolvents, (C) nanozymes with two functional groups, and (D) nanozymes with three functional groups. Protonation states were defined according to the pK_a_ of functional groups at pH 8.4. The functional groups on the nanozymes are colored as follows: red for carboxylate, blue for imidazole, and purple for hydroxyl; the rest of the molecule is colored green. 4NP4HB is colored lime, water (H_2_O) is cyan, and acetonitrile (ACN) is gray. Only the hydrogen of the imidazole’s N_δ_ is shown (colored white) for visual purposes. This color scheme is used throughout the rest of this manuscript. All simulation snapshots were rendered with Visual Molecular Dynamics.^41^

The most active (vide infra) homogeneous nanozyme (FDM-His-MAL, where FDM denotes furandimethanol) was immobilized by functionalizing a 1,6-hexanedithiol SAM prepared on Pd/SiO_2_ (FDM-His-MAL-C6-Pd/SiO_2_). Immobilization as a dithiol SAM chemically anchors FDM-His-MAL on Pd nanoparticles without altering its structural features, thereby potentially preserving its beyond active site interactions after immobilization. The commercial Pd/SiO_2_ catalyst was characterized to have a Pd loading of 4.8 wt % by ICP-OES and a dispersion of 4% as determined by chemisorption. The surface Pd content was calculated to be 0.018 mmol/g of Pd/SiO_2_. Immobilization of the nanozyme was carried out in two steps, formation of a dithiol self-assembled monolayer (SAM), and functionalization of the SAM with FDM-His-MAL, as described in Figure 2B. CHNS elemental analysis was performed after each step to quantify the incorporation of nitrogen (N) and sulfur (S) on the Pd/SiO_2_ surface. Following dithiol SAM formation, sulfur was introduced via the adsorbed dithiol molecules and subsequent functionalization with FDM-His-MAL introduced nitrogen. The CHNS elemental analysis results are summarized in Table S1. Ideally, one end of the dithiol adsorbs on the Pd nanoparticle surface, while the other end reacts with the nanozyme double bond via a thiol–ene reaction. However, as previously reported, SAM formation by dithiols may result in undesirable configurations, as illustrated in Figure S5.^38^ Several studies have indicated that the uptake of alkanethiols on Pd surfaces typically ranges from one-half to one-third of the available surface Pd atoms.^38–40^ ICP-OES analysis of the immobilized FDM-His-MAL nanozyme revealed a sulfur content of 0.085 wt %, corresponding to dithiol content of 0.013 mmol/g. CHNS elemental analysis showed that the formed SAM on Pd/SiO_2_ contained approximately 0.007–0.010 mmol/g of dithiols. The dithiol loading was between 0.39 and 0.72 of the number of surface Pd atoms, consistent with literature values.^38–40^ This result confirms the minimal formation of undesired multilayers. However, the thiols may also lay flat on the Pd where both thiol ends bind to the metal surface, reducing the number of available thiol groups for nanozyme immobilization (Figure S5). Future research is needed to further improve the heterogenization of the nanozyme that we outline in this report.

### 2.2 Reaction Kinetics Analysis and Molecular Geometry Analysis

The catalytic activity of the nanozymes was measured for the hydrolysis of 4NP4HB into 4-hydroxybenzoic acid (4HB) and 4-nitrophenol (4NP) as shown in Figure 4A. The reaction was carried out at 50 °C in 50% acetonitrile and 50% aqueous borate buffer (pH 8.4) by volume. Although ester hydrolysis is promoted by water, a solvent mixture was used because 4NP4HB has low solubility in water. Reactant and product concentrations were monitored through absorbance measurements using UV–vis spectroscopy. As shown in Figure S6, the absorbance spectra are primarily composed of signals from 4NP4HB, 4NP, and 4HB. The nanozymes remained stable during the reaction as demonstrated in Figure S7. Figure S8 presents the conversion, yield, and carbon balance calculated from each sample. For all nanozyme runs, the carbon balance remained above 89% throughout the reaction, indicating that no significant side reactions occurred and that the main products were 4HB and 4NP.

**Figure 4:**
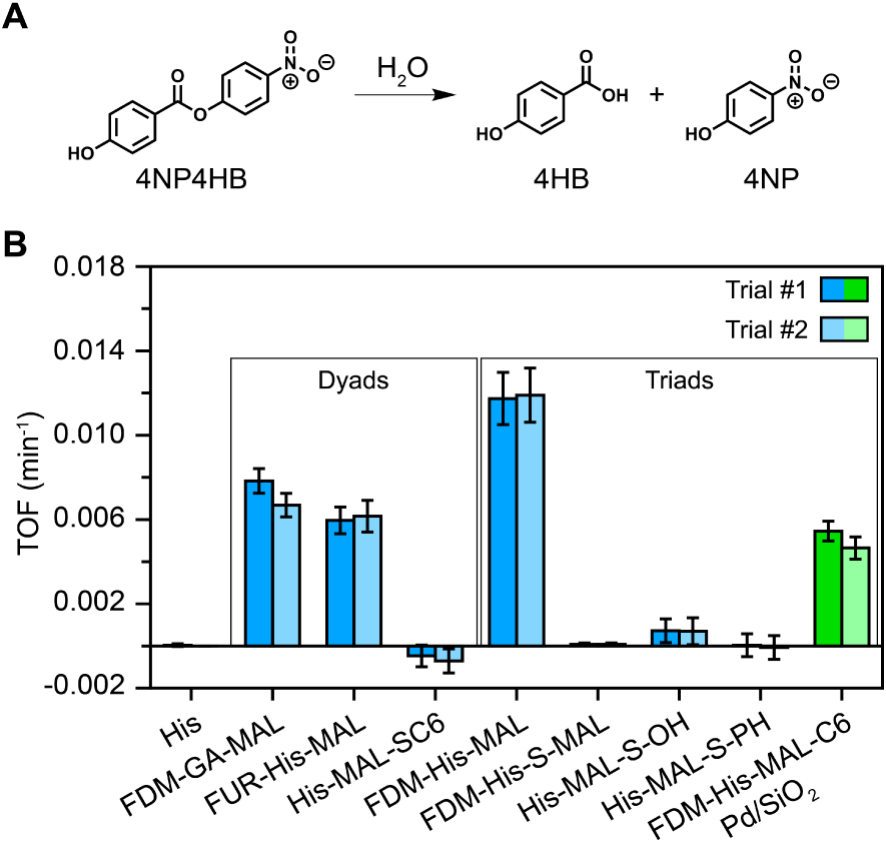
Intrinsic catalytic activity for 4NP4HB hydrolysis. (A) Reaction scheme for the hydrolysis of 4NP4HB into 4HB and 4NP. (B) The intrinsic catalytic activity (TOFs) for model ester hydrolysis at 50 °C in 50% acetonitrile and 50% borate buffer (pH 8.4) by volume with [4NP4HB]_i_ of 1.0 mM. Error was computed as the 95% confidence interval from nonlinear fitting. Homogeneous nanozymes (cat/feed = 0.5 mol/mol) are shown in blue and the heterogenized nanozyme (cat/feed = 0.1 or 0.3 mol/mol) is shown in green. Data are shown for two independent trials.

The apparent rate constant, k_app_ (min^–1^) was obtained by fitting the measured concentrations to a first-order kinetic model under conditions of large excess water. Equations 1 and 2 were used as the kinetic model; 4HB was excluded in the model because 4NP4HB, 4NP, and the nanozyme all absorb at wavelengths close to 4HB’s maximum absorbance (250 nm)—including it would lead to larger error in the model. We calculated k_app_ with 95% confidential intervals by fitting the experimental data to the kinetic model using MATLAB as shown in Figure S10.

1 $$\frac{d\left[4NP4\mathrm{HB}\right]}{\mathrm{dt}}=-k_{\mathrm{app}}\left[4\mathrm{NP}4\mathrm{HB}\right]$$

2 $$\frac{d\left[4\mathrm{NP}\right]}{\mathrm{dt}}=k_{\mathrm{app}}\left[4\mathrm{NP}\right]$$

The normalized rate constant, k_cat_ (min^–1^·M^–1^) was obtained by first subtracting the background hydrolysis activity of the solvent system (k_app,blank_) and the activity of trace amounts of piperidine impurity (k_piperidine_), and dividing by the concentration of the nanozyme catalyst using eq 3. The value of k_cat_ was then multiplied by the initial concentration of the substrate to determine the turnover frequency (TOF) (min^–1^) for the nanozyme (eq 4). The k_cat_ and TOF values obtained from 4NP4HB hydrolysis catalyzed by all nanozymes are summarized in Table S2.

3 $$k_{\mathrm{cat}}=\frac{k_{\mathrm{app}}-k_{\mathrm{app},\mathrm{blank}}-k_{\mathrm{piperidine}}\left[\mathrm{piperidine}\right]}{\left[\mathrm{catalyst}\right]}$$

4 $$\mathrm{TOF}=k_{\mathrm{cat}}\cdot {[4\mathrm{NP}4\mathrm{HB}]}_{i}$$

Figure S9 summarizes k_cat_ based on three catalytic runs performed under the same reaction conditions but with varied initial substrate concentrations. The variation had no significant effect on k_cat_, indicating that the hydrolysis of 4NP4HB follows first-order kinetics with respect to 4NP4HB.

Figure 4B summarizes the TOF of the precursor HIS, the homogeneous nanozymes, and the immobilized nanozyme. While only free HIS is shown in Figure 4B for comparison, the precursors of nanozymes or the individual HIS, GA, and SER amino acids in the catalytic triad exhibited little to no catalytic activity (Table S2 and Figures S11–S12). Possible trace impurities from the nanozyme synthesis, such as *p*-toluenesulfonic acid and piperidine, were also tested; *p*-toluenesulfonic acid showed no activity, while piperidine exhibited low activity. In addition, 4NP4HB hydrolysis catalyzed by a mixture of amino acids, HIS and SER, was performed. Although this mixture contains species that collectively have all the functional groups of the catalytic triad, it exhibited almost no activity (Figure S13). The most active nanozyme, FDM-His-MAL (synthesized by the Diels–Alder reaction), has all three catalytic triad-inspired functional groups as shown in Figure 3D (we refer to such nanozymes as triads) and exhibited ∼300-fold higher activity than free HIS. Nanozymes synthesized by the Diels–Alder route, including FDM-GA-MAL and FUR-His-MAL which have two functional groups as shown in Figure 3C (we refer to such nanozymes as dyads), showed a substantial increase of at least 150-fold higher activity than free HIS. In contrast, nanozymes produced through thiol–Michael addition showed little or no enhancement in catalytic activity for both dyads and triads. FDM-His-S-MAL is a triad nanozyme synthesized from urocanic acid using both the Diels–Alder reaction and thiol–Michael addition. Although this nanozyme incorporates all triad moieties and the Diels–Alder adduct ring, it exhibited almost no activity. These results suggesting that structural features beyond the active site, such as the presence of the Diels–Alder adduct ring or an alkyl-sulfur linkage, may have significant contributions to catalytic performance.

The heterogenized FDM-His-MAL had approximately half the catalytic activity compared to its homogeneous counterpart potentially due to adsorption of both thiol ends to the Pd nanoparticle as discussed previously (Figure S5). This structural orientation may reduce the effective number of accessible functional organic sites within the catalyst. Since the apparent rate constant is normalized by the total number of dithiols present on the catalyst surface, this normalization results in a lower TOF. All homogeneous nanozymes were evaluated at a catalyst-to-feed molar ratio of 0.5 for two trials, except for the highest activity nanozyme, FDM-His-MAL. For this nanozyme, a third trial was performed at a ratio of 0.1 for its homogeneous run, and its immobilized form was evaluated at ratios of 0.1 in the first trial and 0.3 in the second run. Variations in the catalyst-to-feed ratio showed no effect on the intrinsic activity, validating that the observed catalytic performance reflects the true intrinsic activity of the active sites, not external concentration effects.

Figure 4B indicates that incorporating functional groups representative of a catalytic triad into a nanozyme does not necessarily lead to high catalytic activity. For example, the most active nanozyme (FDM-His-MAL) is a triad, but FDM-His-S-MAL, His-MAL-S–OH, and His-MAL-S-PH are triads with low activity. FDM-His-S-MAL differs from FDM-His-MAL by using urocanic acid as the precursor and the incorporation of an alkyl-sulfur linkage. Furthermore, two of the dyad-based catalysts were active. Recently, QM/MM simulations have shown that PET hydrolysis is initiated by an acylation step where the catalytic triad assumes geometries that facilitate ester bond cleavage by SER.^30,31^ Acylation is preceded by the HIS imidazole deprotonating the SER hydroxyl in a tetrahedral conformation at the active site.^30,31,42,43^ This conformation is enabled by hydrogen bonding between the imidizole’s NH_δ_ and the carboxylate from a neighboring ASP, allowing the imidizole’s N_ε_ to become protonated by the SER hydroxyl group. While ab initio methods can model reaction mechanisms directly, our first aim was to identify factors that influence activity by using computationally efficient classical molecular dynamics (MD) simulations that are suitable for sampling spatial distributions of functional groups and local solvent environments^44,45^ for all synthesized nanozymes. We thus used MD simulations^46^ to investigate whether the geometrical arrangement of functional groups in highly active nanozymes would increase the probability of forming this hydrogen bond reported from QM/MM simulations, and to identify descriptors that potentially influence ester hydrolysis.

From MD simulations,^46^ we computed the probability of the NH_δ_–O hydrogen bond (Figures S16 and S17) when 4NP4HB was restrained far from and near each nanozyme, and compared to reference calculations of hydrogen bonding present in PETase with and without a PET dimer near the active site (Figure 5A).^31^ Since the resulting distributions were bimodal (Figure S16), we classified each mode as either the “closed” state, in which there is hydrogen bonding between the imidazole NH_δ_ and nearest carboxylate oxygen (Figure 5B), or the “open” state, in which these groups do not hydrogen bond and are far apart (Figure S18). When 4NP4HB is near the nanozyme, all nanozymes exhibit high probabilities (25–40%) of closed states relative to PETase (<19%). The NH_δ_–O hydrogen bond length is similar for all nanozymes and longer, on average, than the hydrogen bond in the PETase by ∼0.6 Å, although fluctuations in this length are ∼0.4 Å (Table S4) which overlaps with the average length in the PETase (Figures S16 and S19). FDM-His-S-MAL is an exception since its probability of assuming a closed state is less than 10% and even if 4NP4HB is near the nanozyme, NH_δ_–O hydrogen bonding is seldom observed (Figure S16). This minimal hydrogen bonding is consistent with the observed low activity of FDM-His-S-MAL. When 4NP4HB is far from the nanozyme (or active site of PETase), all nanozymes have a substantially smaller probability (7–12%) of assuming the closed state. The NH_δ_–O hydrogen bond lengths we computed were found in agreement with those reported for other hydrolase-inspired multifunctional catalysts that incorporate equivalent functional groups into micelles (∼2.7 Å), which were found to be close to that of α-chymotrypsin (2.64 Å).^23,47^ Furthermore, recent serine hydrolyses designed de novo with exceptionally high catalytic efficiencies were shown to preorganize amino acids into geometries with NH_δ_–O hydrogen bonds measuring 2.8–3.0 Å, further suggesting that these structural geometries are relevant for our nanozymes to hydrolyze ester bonds.^32^ These data show that, like the PETase and α-chymotrypsin, the nanozymes promote NH_δ_–O hydrogen bonding when the substrate is present. However, the resulting spatial arrangements of functional groups appear similar for all nanozymes, with the exception of FDM-His-S-MAL, and cannot explain experimentally observed differences in activity.

**Figure 5:**
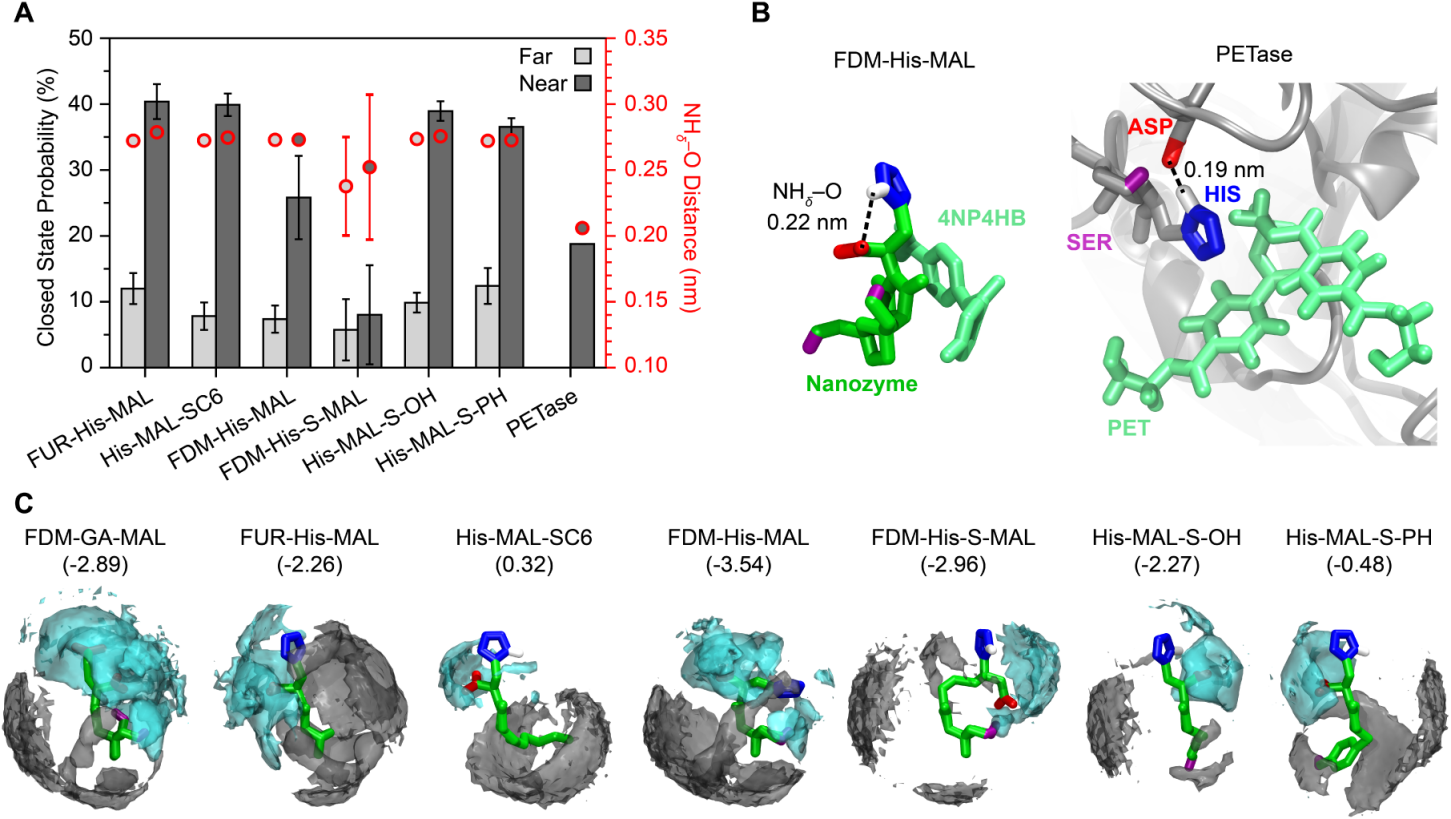
Analysis of molecular geometries during substrate interactions. (A) Computed probability for nanozymes with an imidazole group to achieve closed state conformations (bars, error computed as the standard error between six replicates) and the average distance between the imidazole NH_δ_ and the closest carboxylate oxygen atom (circles, error computed as the standard error between replicates that exhibited a bimodal distribution) in the closed state when 4NP4HB is far from (2.0 nm) or near (0.3 nm) the nanozyme. (B) Representative snapshots of the closed state conformation for FDM-His-MAL and PETase with the ester restrained near either the nanozyme’s maleimide or the enzyme’s catalytic triad. Snapshots illustrate hydrogen bonding between the imidazole NH_δ_ and the closest carboxylate oxygen atom. (C) Spatial distribution functions with isosurfaces drawn at 10 times the average number density normalized by the bulk solvent number density for water (cyan) and acetonitrile (gray) with 4NP4HB restrained near the nanozyme (only the nanozyme is shown in the snapshots for visual clarity). Numbers in parentheses represent the nanozyme octanol–water partition coefficient (LogP) estimated using Crippen’s method^48^ as implemented in Chemdraw 21.0.0.

We next investigated whether solvent effects may influence nanozyme activity by controlling the local chemical environment, following past studies that have demonstrated that organic solvent mixtures can affect Brønsted-acid-catalyzed reactions.^44,45,49^ Figure 5C shows spatial distribution functions (SDFs) of water and acetonitrile for configurations where 4NP4HB is near the nanozyme. The SDFs describe the three-dimensional number density of water or acetonitrile around the nanozyme-4NP4HB structure. Figure 5C shows that the local solvent environment near high-activity nanozymes (FDM-GA-MAL, FUR-His-MAL, and FDM-His-MAL) has large domains of water and acetonitrile, whereas the environment near low-activity nanozymes (His-MAL-SC6, His-MAL-S–OH, and His-MAL-S-PH) is enriched in acetonitrile. FDM-His-S-MAL is again an exception in that it is a low-activity nanozyme that is still surrounded by moderately sized cosolvent domains, but has limited NH_δ_–O hydrogen bonding (Figure 5). These results support the hypothesis that hydrogen bonding between the imidazole NH_δ_ to either of the carboxylate’s O along with the local solvent environment are important in mediating hydrolysis of 4NP4HB. These localized domains suggest that access to both water, as the reaction-promoting solvent, and acetonitrile, as the solubility-promoting cosolvent, promotes catalytic activity. These observations are in agreement with how polar aprotic cosolvents can compete against water to influence selectivity and change reaction mechanisms.^45^ These data, although not indicative of specific reaction mechanisms, provide insight into the complex interplay of nanozyme-substrate-solvent interactions that can help elucidate design factors and inform future and more detailed mechanistic studies.

Based on these observations, we studied whether the local water concentration would be higher near more hydrophilic nanozymes and lead to higher activity. We calculated the octanol–water partition coefficient, LogP, as a descriptor of nanozyme hydrophilicity to compare with activity trends (Figure 5C). Decreasing LogP indicates increased hydrophilicity, with LogP < 0 indicating a thermodynamic preference for water compared to octanol as a hydrophobic organic solvent. FDM-His-MAL exhibits the highest activity and is the most hydrophilic nanozyme (LogP = −3.54) whereas His-MAL-SC6 has the lowest activity and is the least hydrophilic (LogP = 0.32). Similarly, the trend in activity follows the trend in LogP for both the triads and dyads. However, the dyad FUR-His-MAL (LogP = −2.26) has substantially higher activity than the triad His-MAL-S–OH (LogP = −2.27) even though they have almost identical values of LogP. This analysis indicates that nanozyme hydrophilicity impacts activity. Since nanozyme hydrophilicity also depends upon features beyond the active site (e.g., the acyl chain in His-MAL-SC6), these findings underscore the challenges in nanozyme design due to the complex interplay between intra- and intermolecular interactions of functional groups and the surrounding local environment.

### 2.3 Solvent Effect and Local Chemical Environment

To further explore potential solvent effects and the role of nanozyme hydrophilicity, Figure 6A compares the activity of His-MAL-SC6 and FDM-His-MAL in 50% and 25% v/v acetonitrile (Table S6). Decreasing acetonitrile content had a negligible effect on TOF for His-MAL-SC6 but increased TOF by ∼2-fold for the more hydrophilic FDM-His-MAL. Based on our previous data (Figures 4 and 5), we suggest that increased access to water near the nanozyme may increase catalytic activity. To quantify this effect, we computed Γ* as a descriptor of preferential hydration as a function of the distance, r, from any atom in the nanozyme (Figure 6B). Values of Γ* > 1 indicate that water is enriched at that value of r, relative to the overall solvent content, and Γ* < 1 indicates that water is preferentially excluded (eq 11). This descriptor has been previously used to demonstrate that polyol molecules form a thin layer of water around the contour of proteinase inhibitors and lysozyme.^50,51^ Figure 6C shows Γ* in 0.1 nm intervals for configurations with 4NP4HB near the nanozyme.

**Figure 6:**
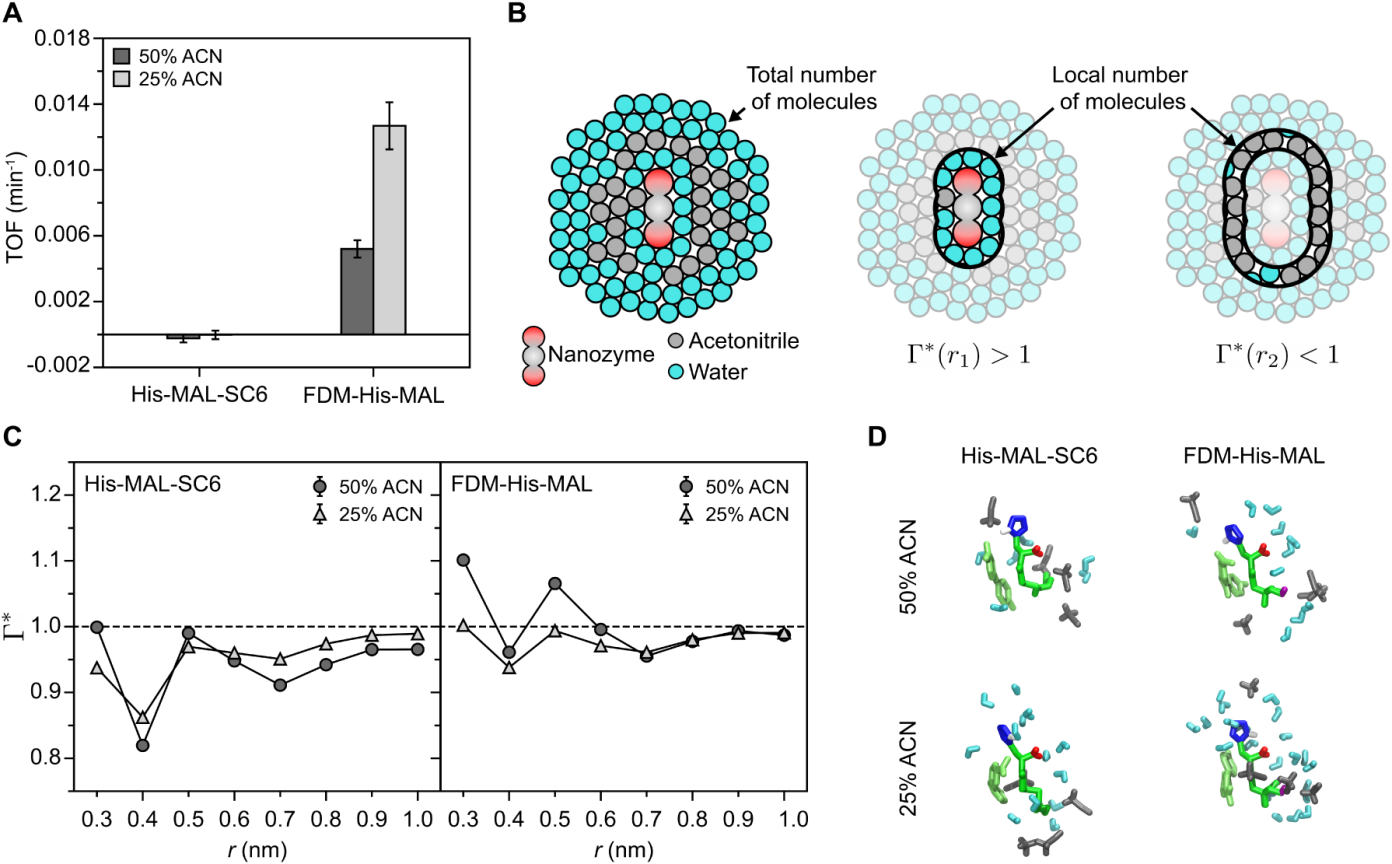
Solvent effects on the highest- and lowest-activity nanozymes. (A) Catalytic activity (TOFs) for model ester hydrolysis at 50 °C in 50% and 25% acetonitrile by volume with [4NP4HB]_i_ of 0.5 mM (cat/feed = 0.5 mol/mol). Error was computed as the 95% confidence interval from nonlinear fitting. (B) Schematic illustrating how the preferential hydration is calculated at two representative distances r_1_ and r_2_. (C) Preferential hydration profiles, Γ* calculated at radii r from each nanozyme atom. Error was computed as the standard error between six replicates (smaller than the markers). (D) Representative simulation snapshots for r = 0.4 nm.

For His-MAL-SC6, at both acetonitrile concentrations, Γ* indicates a sharp depletion of water at 0.4 nm (representative snapshots in Figure 6D). This depletion behavior indicates the formation of a microheterogeneous solvent environment, which is well-known in aqueous acetonitrile mixtures.^52^ For FDM-His-MAL, values of Γ* are generally larger than for His-MAL-SC6 due to the increased hydrophilicity of the nanozyme, but there is still a similar depletion of water at 0.4 nm in 50% v/v acetonitrile. Decreasing from 50% to 25% acetonitrile decreases the enrichment of water locally, rather than increasing it (while increasing the local concentration of water molecules, as shown in Table S5), indicating that water enrichment alone does not explain the increased activity in 25% acetonitrile. However, the Γ* profile is flatter with values close to unity, which indicates a more homogeneous local solvent environment since neither water nor acetonitrile is preferentially excluded. The observation of a more homogeneous solvent environment promoting higher activity is consistent with previous studies involving water-organic solvent mixtures.^44,53–56^ This behavior was likewise attributed to the diminished microheterogeneity of acetonitrile at dilute concentrations in which solvent clusters are not formed, which allows acetonitrile molecules to access water cavities and increase the homogeneity of the microenvironment.^57–60^ We thus expect that this homogeneous environment ensures access of the nanozyme to water (to promote hydrolysis) and the substrate to acetonitrile (to stabilize the substrate) in the local chemical environment, which could reduce solvent reorganization energies to improve activity as summarized in Figure 1E.^61,62^

### 2.4 Comparison to a Commercial Ester Hydrolysis Enzyme

We assessed the performance of FDM-His-MAL compared to an enzyme, α-chymotrypsin, as shown in Table 2 and Figure S21. α-Chymotrypsin is one of the most extensively studied serine hydrolase for small ester hydrolysis, with detailed investigations into its catalytic mechanism and stability.^33,34^ This enzyme acts as an efficient catalyst through a catalytic triad that operates via a charge-relay system, similar to the mechanism observed in PETase.^23,63^ The acetonitrile composition was reduced to 5% v/v in water to ensure sufficient enzyme activity. The 4NP4HB feed concentration was reduced to one-tenth the values used in the 50% v/v acetonitrile experiments due to the low solubility of 4NP4HB in water. At ambient temperature, FDM-His-MAL showed comparable activity to α-chymotrypsin in the 5% v/v acetonitrile system. Increasing the acetonitrile content to 50% resulted in a decrease in k_cat_ for both catalysts. However, the k_cat_ of FDM-His-MAL remained ∼4-fold higher than that of α-chymotrypsin. We note that because the experiment at 50% acetonitrile was conducted at far higher substrate concentration, the TOF increased despite this decrease in k_cat_ as shown in Figure 7. At a higher temperature of 50 °C and 5% v/v acetonitrile, the activity of FDM-His-MAL was twice that of α-chymotrypsin. Elevated reaction temperatures and increased organic solvent content decreased the activity of α-chymotrypsin, while enhancing the TOF of FDM-His-MAL.

**Table 2:** Summary of Intrinsic Activity of α-Chymotrypsin and FDM-His-MAL for 4NP4HB Hydrolysis at Cat/Feed = 1:10 for 6 h

Catalyst	Temperature	ACN (% v/v)	[4NP4HB]_i_ (mM)	[cat] (mM)	k_cat_ (min^–1^·M^–1^)	TOF (min^–1^)
	RT^a^	5	0.096	0.010	34.1	0.0033
α-Chymotrypsin	RT^a^	50	0.937	0.098	0.9	0.0008
	50 °C	5^b^	0.097	0.010	28.1	0.0027
	RT^a^	5	0.098	0.010	30.8	0.0030
FDM-His-MAL	RT^a^	50	0.952	0.104	4.0	0.0039
	50 °C	5^b^	0.104	0.010	59.8	0.0062

a For the runs conducted at room temperature, background hydrolysis activity was negligible (k_app,blank_ and k_piperidine_ as zero).

b For the runs conducted at 50 °C and 5% v/v ACN, piperidine activity was negligible (k_piperidine_ as zero).

**Figure 7:**
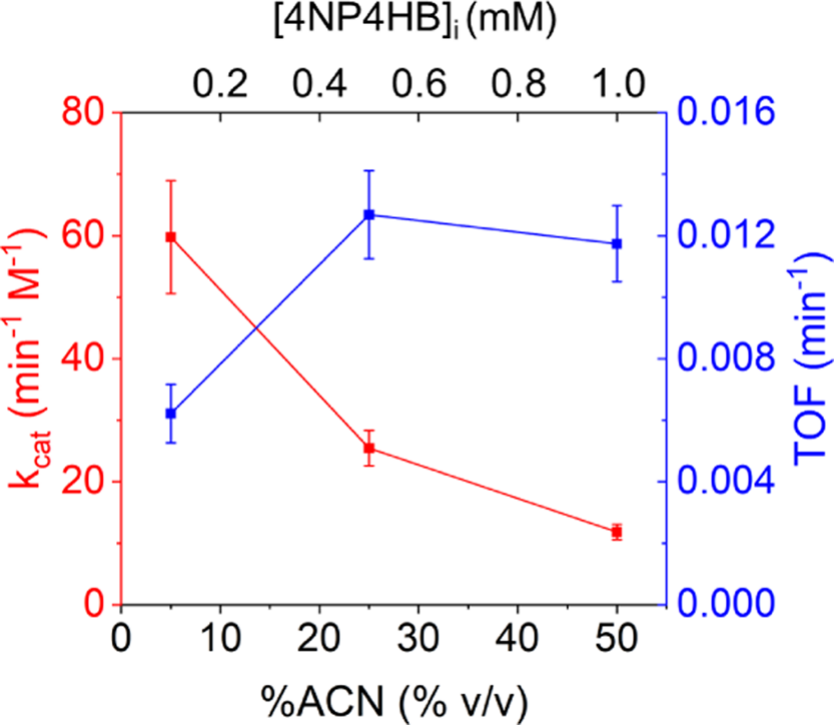
Intrinsic catalytic activity (k_cat_ and TOF) of nanozyme FDM-His-MAL and the corresponding initial substrate concentrations ([4NP4HB]_i_) near the solubility limit of 4NP4HB for model ester hydrolysis at 50 °C in various composition of acetonitrile and borate buffer (pH 8.4) solvent system. Error was computed as the 95% confidence interval from nonlinear fitting.

## 3 Conclusions

We have developed a new synthetic approach for the creation of organic molecular nanozymes capable of catalyzing ester hydrolysis in high organic solvent mixtures and sustaining activity at elevated temperatures. By incorporating functional groups found in enzyme catalytic triads, the most active nanozyme achieves catalytic activities comparable to or higher than a commercial enzyme. Compared to enzymes, key benefits of the nanozyme catalysts include increased activity at temperatures that cause enzyme deactivation and increased activity in high organic solvent concentrations that enable high substrate loading. A method of heterogenization without structural modification of the most active nanozyme, utilizing a dithiol SAM, was also developed to enable efficient removal of the immobilized nanozyme from the reaction mixture. Further investigation into the recyclability of the immobilized nanozyme will be conducted in future work. Analysis of nanozymes using MD simulations indicates that activity trends relate to the spatial distribution of functional groups as well as solvent molecules that collectively define the local chemical environment, leading to the identification of descriptors of nanozyme hydrophilicity and solvent microdomains. While demonstrated for model ester hydrolysis, we expect these principles and molecular-scale insights will generalize to the future design, optimization, and mechanistic analysis of new nanozymes as a class of bioinspired catalysts. Our molecular design was initially motivated by the serine-hydrolase active site (i.e., its catalytic triad), but our data suggest the presence of additional ester hydrolysis pathways, particularly ones where solvent effects play a major role. Therefore, in future work, we will build on these findings to explore other solvents that can readily control the local solvation of species, and further develop our heterogenization approaches. Future computational studies will focus on applying the MD-derived insight presented in this work to guide ab initio calculations aimed at elucidating potential mechanistic pathways that our nanozymes follow for ester hydrolysis.

## 4 Materials and Methods

### 4.1 Synthesis of *N* ^τ^-Tosylhistidine (**1**) by Selective Removal of Boc Protection

7.3 g (18 mmol) of Boc-His­(Tos)–OH (*N* ^α^-(*tert*-butoxycarbonyl)-*N* ^τ^-tosylhistidine, Chem-Impex, 99.69% by HPLC) was added to 180 mL of ethyl acetate (EtOAc, Fisher Scientific, HPLC grade) in a round-bottom flask. The mixture was sonicated and stirred until it appeared as homogeneous as possible, forming a cloudy solution. While stirring, 17.3 g (180 mmol) of methanesulfonic acid (MSA, Sigma-Aldrich, ≥99.0%) was slowly added and reacted at room temperature for 18 h. After the addition of MSA, the solution will quickly turn transparent, then become cloudy again, eventually leading to phase separation. Stirring was stopped during the last 1 h of the reaction to allow the EtOAc and MSA phases to separate and the top EtOAc phase was removed. 60 mL of saturated aqueous sodium bicarbonate solution prepared by dissolving sodium bicarbonate (Sigma-Aldrich, ACS reagent, ≥99.7%) in Mili-Q water was added dropwise with stirring to precipitate out **1**. The precipitated product was collected and dried by vacuum filtration. The yield of **1** was approximately 70% with a small amount of sodium methanesulfonic acid as the impurity. The moisture content of **1** was quantified by ^1^H NMR with dimethylformamide as the internal standard.

Compound **1a**: ^1^H NMR (400 MHz, DMSO-d_6_) δ 8.96 (d, *J* = 1.3 Hz, 1H), 7.48 (d, *J* = 8.1 Hz, 2H), 7.45 (d, *J* = 1.3 Hz, 1H), 7.14 (d, *J* = 7.8 Hz, 2H), 3.24 (qd, *J* = 15.5, 7.1 Hz, 2H), 2.27 (s, 3H).

### 4.2 Synthesis of Protected Histidine-Maleimide Complex (**2a**) by Substitution Reaction^35,36^

1.9 g (6.2 mmol) of **1** and 0.8 g (7.2 mmol) of sodium carbonate (Sigma-Aldrich, ReagentPlus, ≥99.5%) were dissolved in 18 mL of water and 18 mL of tetrahydrofuran (THF, Sigma-Aldrich, ACS reagent, ≥99.0%) contains 250 ppm BHT as inhibitor) in a round-bottom flask. While stirring, 1.1 g (7.2 mmol) of *N*-(methoxycarbonyl)­maleimide (Ambeed, 98%) was slowly added and reacted at room temperature for 4 h. During the first 30 min of the reaction, the pH was monitored, and additional sodium carbonate was added if necessary to maintain the pH at 8–9. After the reaction, aqueous 2 M HCl (Supelco, Titripur) was added to acidify the solution to a pH 2–3. About 0.5 g of citric acid monohydrate (Sigma-Aldrich, ACS reagent, ≥99.0%) was added to the solution and the aqueous solution was adequately extracted with dichloromethane (DCM, Sigma-Aldrich, ≥99.8% suitable for HPLC) for four times. The organic layer was dried over sodium sulfate (Sigma-Aldrich, ACS reagent, ≥99.0% anhydrous) and filtered by vacuum filtration. The resulting product solution was then concentrated to approximately 20 mL with a rotary evaporator at 30 °C. The estimated yield of **2a** was 42%. Due to the low stability of concentrated **2a**, the product solution was directly used in the subsequent step without further purification.

Compound **2a**: ^1^H NMR (500 MHz, DMSO-d_6_) δ 8.10 (d, *J* = 1.4 Hz, 1H), 7.84 (d, *J* = 8.5 Hz, 2H), 7.44 (m, 2H), 7.29 (d, *J* = 1.4 Hz, 1H), 4.80 (dd, *J* = 9.8, 6.1 Hz, 1H), 3.23–3.17 (m, 2H), 2.41 (s, 3H).

### 4.3 Synthesis of FDM-His-MAL and FUR-His-MAL by Diels–Alder reaction,^37^ Selective Hydrogenation and Tosyl Deprotection^64^

For the synthesis of FDM-His-MAL, 0.6 g (4.6 mmol) of 2,5-furandimethanol (FDM, Wuhan Golden Wing Industry, 98%), or for FUR-His-MAL, 1.0 mL (13.8 mmol) of furan (Sigma-Aldrich, ≥99%), was added to 1.0 g (2.6 mmol) of **2a** in about 20 mL of a DCM/THF cosolvent. The reaction mixture was stirred at room temperature for 18 h. Afterward, DCM was removed under reduced pressure, and the mixture was concentrated to approximately 8 mL using a rotary evaporator at 35 °C. The reaction was then continued at room temperature for an additional 6 h. For the synthesis of FUR-His-MAL, an extra 0.2 mL of furan was added after the rotary evaporation step. The conversion of **2a** was nearly full yielding the Diels–Alder adducts **3a** or **3b**.

Without purification, the selective hydrogenation of the Diels–Alder adduct’s ring double bond was performed to prevent retro-Diels–Alder reaction. To the solution of **3a** or **3b** in THF, 8 mL of water, a trace amount of copper­(II) acetate monohydrate (Sigma-Aldrich, ACS reagent, ≥98%), and 1.0 g of hydrazine hydrate (20.0 mmol, Sigma-Aldrich, reagent grade, 50–60% hydrazine) were added. The reaction mixture was stirred at room temperature under a continuous flow of air for 18 h. After completion, aqueous 2.5 M sulfuric acid (Supelco, Titripur) was added dropwise until the pH reached 2–3, and the mixture was sonicated for 1 h to precipitate excess hydrazine as hydrazine sulfate. The precipitate was removed by 2.2 μm syringe filter. Calcium carbonate (Sigma-Aldrich, ACS reagent, 99.0% powder) was added to the filtrate and sonicated for 3 h until the pH approached close to neutrality. The resulting precipitated calcium sulfate was removed by 2.2 μm syringe filter. The solution was concentrated using a rotary evaporator at 40 °C. The conversion of **3a** or **3b** was close to 100% yielding **4a** or **4b**.

Subsequently, 5.0 mL of ethanol (EtOH, Sigma-Aldrich, 200 proof, ACS reagent, ≥99.5%) and 1.0 g (11.7 mmol) of piperidine (Sigma-Aldrich, ReagentPlus, 99%) were added to the solution containing about 2.6 mmol of **4a** or **4b**. Water was added as needed to fully dissolve the reactants. The reaction mixture was stirred at room temperature for 24 h. The crude product solution was concentrated by a rotary evaporator at 45 °C and purified by flash column chromatography.

Chromatography separation was performed on a Büchi Pure C-815 flash chromatography system equipped with a Büchi FlashPure EcoFlex C_18_ 120 g reverse-phase column, with 1.5 g of crude product per injection, using a water (Mili-Q water)/acetonitrile (Fisher Scientific, HPLC grade) mobile phase. Elution was carried out with a gradient from 0% to 5% acetonitrile over 7 column volumes, followed by a rapid increase from 5% to 100% acetonitrile over 0.5 column volumes, and then an isocratic elution at 100% acetonitrile for 3 column volumes. The column was washed with pure water and acetonitrile between runs. The purified FDM-His-MAL contained trace amounts of FDM and piperidine as impurities. Similarly, the purified FUR-His-MAL contained small amounts of *p*-toluenesulfonic acid and piperidine, and exhibited both endo and exo conformers. The actual weight percent was quantified using ^1^H NMR with either dimethylformamide (Sigma-Aldrich, anhydrous, 99.8%) or acetic acid (Sigma-Aldrich, ReagentPlus, ≥99%) as the internal standard.

Compound FDM-His-MAL: ^1^H NMR (500 MHz, D_2_O) δ 7.68 (d, *J* = 1.2 Hz, 1H), 6.97 (s, 1H), 4.83 (s, 1H), 3.86 (s, 4H), 3.50 (s, 2H), 3.13–2.89 (m, 2H), 1.75–1.50 (m, 4H).

Compound FUR-His-MAL: ^13^C qNMR (500 MHz, D_2_O) δ 177.92 (0.5C), 176.50 (1C), 172.59 (1.5C), 134.06 (1C), 127.53 (1C), 117.76 (1C), 79.32 (0.5C), 77.97 (1.5C), 53.63 (1.5C), 49.44 (1C), 48.19 (0.5C), 27.96 (0.5C), 25.82 (1.5C), 25.68 (1C).

### 4.4 Synthesis of His-MAL-S–OH, His-MAL-S-PH, and His-MAL-SC6 by Thiol–Michael Addition and Tosyl Deprotection^64^

Triethylamine (TEA, Sigma-Aldrich, ≥99.5%) was added to 1.0 g (2.6 mmol) of **2a** in about 20 mL of a DCM/THF cosolvent until the pH reached 8–9. Ar gas (Airgas) was bubbled through the solution for 20 min to remove dissolved oxygen from the solution and the reaction vessel. 1.0 g (12.8 mmol) of 2-mercaptoethanol (Thermo Scientific, Electrophoresis grade, ≥98%) for the synthesis of His-MAL-S–OH, 1.0 g (7.9 mmol) of 4-mercaptophenol (Sigma-Aldrich, 97%) for His-MAL-S-PH, or 1.0 g (8.5 mmol) 1-hexanethiol (Sigma-Aldrich, 95%) for His-MAL-SC6 was added and the reaction vessel was tightly sealed. The reaction mixture was stirred at room temperature for 18 h. After the reaction, the solution was concentrated using a rotary evaporator at 35 °C. The conversion of **2a** was nearly full yielding **3c**, **3d**, or **3e**.

Subsequently, 5.0 mL of ethanol (EtOH) and 1.0 g (11.7 mmol) of piperidine was added to the solution containing about 2.6 mmol of **3c**, **3d**, or **3e**. Water was added as needed to fully dissolve the reactants. The reaction mixture was stirred at room temperature for 24 h. The crude product solution was concentrated by a rotary evaporator at 45 °C and purified by flash column chromatography.

Chromatographic separation was performed using the same procedure as for the purification of FDM-His-MAL and FUR-His-MAL. The purified His-MAL-S–OH, His-MAL-S-PH and His-MAL-SC6 contained a trace amount of piperidine as the impurity. The actual weight percentages were quantified using ^1^H NMR by either dimethylformamide or acetic acid as the internal standard.

Compound His-MAL-S–OH: ^1^H NMR (500 MHz, D_2_O) δ 7.82 (d, *J* = 1.2 Hz, 1H), 6.94 (t, *J* = 1.6 Hz, 1H), 4.85 (d, *J* = 10.9 Hz, 13H), 3.77 (t, *J* = 6.2 Hz, 2H), 3.72 (t, *J* = 6.4 Hz, 1H), 2.86–2.82 (m, 2H), 2.67–2.59 (m, 2H), 2.41 (d, *J* = 7.5 Hz, 2H).

Compound His-MAL-S-PH: ^1^H NMR (500 MHz, D_2_O) δ 7.91 (d, *J* = 1.3 Hz, 1H), 6.91–6.77 (m, 4.5H), 6.43 (s, 0.5H), 4.72 (ddd, *J* = 11.0, 4.7, 2.2 Hz, 1H), 3.75 (s, 1H), 3.36–3.20 (m, 2H), 2.66 (dd, *J* = 27.0, 19.0 Hz, 2H).

Compound His-MAL-SC6: ^1^H NMR (500 MHz, D_2_O) δ 7.70 (dd, *J* = 4.1, 1.2 Hz, 1H), 6.88 (d, *J* = 9.4 Hz, 1H), 4.86 (m, 1H), 3.63 (t, *J* = 7.6 Hz, 1H), 2.90 (ddd, *J* = 107.1, 16.3, 7.8 Hz, 2H), 2.70–2.63 (m, 2H), 2.60–2.37 (m, 2H), 1.63–1.20 (m, 8H), 0.89 (t, *J* = 3.7 Hz, 3H).

### 4.5 Synthesis of FDM-His-S-MAL from Urocanic Acid by Both Diels–Alder Reaction and Thiol–Michael Addition

Urocanic acid (Thermo Scientific, 98%) was dissolved in MeOH, and sulfuric acid (10 mol %) was added. The reaction mixture was stirred at room temperature overnight. After completion, MeOH was removed under reduced pressure. The residue was washed with NaHCO_3_ aqueous solution to neutralize and remove sulfuric acid, affording complete conversion of urocanic acid to **1b**.

0.675 g of cystamine dihydrochloride (Chem-Impex, ≥98%) was dissolved in 20 mL of saturated NaHCO_3_ aqueous solution with ice bath. While stirring, 0.97 g *N*-(methoxycarbonyl)­maleimide was slowly added. An additional 10 mL of saturated NaHCO_3_ solution and 30 mL of THF were added, and the reaction mixture was allowed to warm to room temperature and stirred for 3 h. During the first 30 min of the reaction, the pH was monitored, and additional sodium carbonate was added if necessary to maintain the pH at 8–9. After completion, the mixture was acidified to pH 2–3 with 2 M aqueous HCl. The aqueous solution was adequately extracted with EtOAc for three times. The organic layer was dried over sodium sulfate and filtered by vacuum filtration. The yield of **2c** was approximately 60%. The resulting EtOAc solution containing compound **2c** was used directly in the subsequent step without further purification.^65^ 0.78 g of FDM was added to the EtOAc solution of **2c**. The reaction mixture was stirred at room temperature overnight. After concentration and filtration, an off-white precipitate of compound **3g** was collected. NMR analysis confirmed full conversion to **3g**.

0.79 g of **3g** was dissolved in 20 mL ACN and 15 mL MeOH. 0.4 mL of hydrazine hydrate and 3 mg of CuAc2·H2O were added to the system with agitation and bubbling air. After 3 h, the solvent was removed under reduced pressure, yielding compound **4c** in full conversion. **4c** was dissolved in a mixture of ACN and MeOH. Excess 1,4-butanedithiol and a catalytic amount of base were added. The reaction vial was sealed and stirred overnight. Excess 1,4-butanedithiol was removed by extraction with ethyl ether, affording the reduced **5**. The reduced **5** was dissolved in MeOH together with 1 equiv of **1b**. The solution was purged with nitrogen before adding catalytic amount of NaOH. The system was then sealed and stirred overnight. NMR suggested full conversion of **1b** to FDM-His-S-MAL.

Compound **1b**: ^1^H NMR (400 MHz, MeOH-4_d_) δ 8.37 (d, J = 19.2 Hz, 1H), 7.67 (d, J = 5.7 Hz, 1H), 7.60 (d, J = 16.0 Hz, 1H), 6.51 (dd, J = 16.0, 2.3 Hz, 1H), 3.78 (s, 3H).

Compound **2c**: ^1^H NMR (400 MHz, DMSO-d_6_) δ 6.91 (s, 4H), 3.66 (t, *J* = 6.4 Hz, 4H), 2.85 (t, *J* = 6.4 Hz, 4H).

Compound **4c**: ^1^H NMR (400 MHz, DMSO-d_6_) δ 3.67 (s, 8H), 3.64 (d, *J* = 6.5 Hz, 4H), 2.92 (q, *J* = 5.6 Hz, 4H), 1.75–1.39 (m, 8H).

Compound **5**: ^1^H NMR (500 MHz, DMSO-d_6_) δ 3.67 (s, 4H), 3.53–3.47 (m, 2H), 3.17 (s, 2H), 2.01–1.95 (m, 2H), 1.67–1.29 (m, 4H).

Compound FDM-His-S-MAL: ^1^H NMR (500 MHz, DMSO-d_6_) δ 7.58 (d, *J* = 11.4 Hz, 1H), 6.97 (d, *J* = 15.8 Hz, 1H), 3.65 (s, 4H), 3.54 (m, 2H), 2.85–2.68 (m, 5H), 1.39–1.29 (m, 4H).

### 4.6 Synthesis of Glutamic Acid-Maleimide Complex (**2b**) by Substitution Reaction^35,36^

0.6 g (4.0 mmol) of l-glutamic acid (Sigma-Aldrich, ReagentPlus, ≥99%) by HPLC) was dissolved in 18 mL of saturated aqueous sodium bicarbonate solution and 18 mL of THF in a round-bottom flask and cooled to 0 °C in an ice bath. While stirring, 0.6 g (4.0 mmol of *N*-(methoxycarbonyl)­maleimide was slowly added and the reaction was carried out from 0 °C to room temperature over 4 h. After 10 min of reaction, an additional 18 mL of saturated aqueous sodium bicarbonate solution and 18 mL of THF were added. During the first 30 min of the reaction, the pH was monitored, and an additional sodium bicarbonate solution was added if necessary to maintain the pH at 8–9. After the reaction, aqueous 2 M HCl was added to acidify the solution to a pH 2–3. The aqueous solution was adequately extracted with EtOAc for four times. The organic layer was dried over sodium sulfate and filtered by vacuum filtration. The resulting product solution was then dried by a rotary evaporator at 30 °C. The estimated yield of **2b** is 49%.

Compound **2b**: ^13^C qNMR (500 MHz, DMSO-d_6_) δ 173.82 (1C), 170.82 (2C), 170.71 (1C), 134.98 (2C), 51.35 (1C), 30.69 (1C), 24.27 (1C).

### 4.7 Synthesis of FDM-GA-MAL by Diels–Alder Reaction and Selective Hydrogenation^37^

0.7 g (2.0 mmol) of **2b** was dissolved in 5 mL of THF. 0.6 g (4.6 mmol) of FDM was added and the reaction mixture was stirred at room temperature for 24 h. The conversion of **2b** was nearly full yielding the Diels–Alder adduct, **3f**.

Without purification, the selective hydrogenation of the Diels–Alder adduct’s ring double bond was performed to prevent retro-Diels–Alder reaction. To the solution of **3f** in THF, 5 mL of water, a trace amount of copper­(II) acetate monohydrate, and 1.0 g of hydrazine hydrate (20.0 mmol) were added. The reaction mixture was stirred at room temperature under a continuous flow of air for 18 h. After completion, aqueous 2.5 M sulfuric acid was added dropwise until the pH reached 1–2, and the mixture was sonicated for 1 h to precipitate excess hydrazine as hydrazine sulfate. The precipitate was removed by 2.2 μm syringe filter. Calcium carbonate was added to the filtrate and sonicated for 3 h until the pH approached close to neutrality. The resulting precipitated calcium sulfate was removed by 2.2 μm syringe filter. The solvent was evaporated using a rotary evaporator at 45 °C. The conversion of **3f** is approximately 100% yielding FDM-GA-MAL.

The crude product was purified via selective precipitation. It was dissolved in the minimum volume of water, followed by the gradual addition of THF until a viscous precipitate formed. The supernatant THF phase was removed, and the precipitated product was washed with diethyl ether (Sigma-Aldrich, anhydrous, ACS reagent, ≥99.0% contains BHT as inhibitor). The purified FDM-GA-MAL was then dried using a rotary evaporator at 45 °C. The purified FDM-GA-MAL contained trace amount of FDM as the impurity. The actual weight percent was quantified using ^1^H NMR by either dimethylformamide or acetic acid as the internal standard.

Compound FDM-GA-MAL: ^13^C qNMR (500 MHz, DMSO-d_6_) δ 178.59 (1C), 177.16, 176.99 (2C), 172.93 (1C), 89.44, 89.27 (2C), 61.52, 61.48 (2C), 55.51 (1C), 51.22, 51.07 (2C), 34.82 (1C), 27.89, 27.73 (2C), 25.11 (1C).

### 4.8 NMR Analysis

^1^H, ^13^C quantitative, and 2D HC HMBC NMR spectra were acquired using either a Bruker Avance-500 spectrometer equipped with a DCH cryoprobe or a Bruker Avance-400 spectrometer equipped with a BBFO probe. Spectral analysis was performed using MestReNova software. Deuterated solvents (DMSO-d_6_ or D_2_O) were used as internal standards for chemical shift referencing.

### 4.9 Preparation of a Self-Assembled Monolayer (SAM) of 1,6-Hexanedithiol on Pd/SiO_2_ ^39,40^

One g of 5 wt % Pd/SiO_2_ (Strem Chemicals, reduced and dry, powder) was reduced under 100 sccm flow of hydrogen (Airgas, Ultra-High Purity) at 250 °C for 1 h with a temperature ramp rate of 2 °C·min^–1^. In a round-bottom flask containing 90 mL of EtOH, Ar gas was bubbled through the solution for 20 min to remove dissolved oxygen from the solution and the reaction vessel. 66 μL of 1,6-hexanedithiol (TCI, ≥97.0%) was added and the flask was tightly sealed. The reduced Pd/SiO_2_ was transferred into the round-bottom flask inside a glovebox and the mixture was stirred under an inert atmosphere at room temperature for 16 h. The reacted solids were collected by centrifugation and washed four times with approximately 50 mL of EtOH each time using vortex mixing and centrifugation. During the final wash, the solids were soaked in EtOH for more than 2 h. After washing, the Pd/SiO_2_ with a dithiol SAM was dried in a vacuum oven at 60 °C for 16 h followed by drying at room temperature for 24 h.

### 4.10 Functionalization of Dithiol SAM on Pd/SiO_2_ By Thiol–Ene Reaction

A solution of 1.3 mmol of compound **3a** in approximately 8 mL of THF was prepared in a round-bottom flask. To this solution, 20 mL of water was added, resulting in phase separation. Argon gas was bubbled through the mixture for 20 min to remove dissolved oxygen from both the solution and the reaction vessel. One g of 1,6-hexanedithiol SAM on Pd/SiO_2_ was added to the solution, and the flask was tightly sealed. The reaction mixture was stirred at 80 °C in an oil bath and reacted for 22 h. To prevent overpressurization and air ingress, a plastic tube connected to a water condenser was attached to the flask. After the reaction, the solids were collected by centrifugation and washed with approximately 50 mL of EtOH once using vortex mixing and centrifugation. The washed solids were transferred to a round-bottom flask, followed by the addition of 10 mL of ethanol and 0.5 g (5.6 mmol) of piperidine. The reaction mixture was stirred at room temperature for 24 h. The resulting FDM-His-MAL functionalized 1,6-hexanedithiol SAM on Pd/SiO_2_ were collected by centrifugation, then washed and dried using the same procedure as described for SAM preparation.

### 4.11 Characterization of Pd/SiO_2_, 1,6-Hexanedithiol SAM on Pd/SiO_2_, and FDM-His-MAL Functionalized Dithiol SAM on Pd/SiO_2_

Metal dispersion of the catalyst was determined via static carbon monoxide (CO) chemisorption in a Micromeritics 3-Flex instrument. To accomplish this, 50 mg of catalyst was loaded into a quartz flow-through sample tube and was degassed at 383 K (10 K·min^–1^) for 0.5 h prior to in situ reduction under flowing H_2_ (Airgas, Ultra-High Purity) at 673 K (10 K·min^–1^) with a 30 min hold. The sample was then evacuated before cooling to analysis temperature (308 K). Total CO uptake was assessed via an adsorption isotherm collected between 0 and 10 Torr (increments of 0.5–1.0 Torr). Weakly bound (physisorbed) CO was removed through evacuation of the sample, and the reversible CO uptake was assessed via a second adsorption isotherm. The first and second isotherms were used to determine the irreversible CO uptake by linearly extrapolating the difference of each of the points to the limit of zero pressure. Stoichiometry of the CO adsorption was assumed to be 1.5.

Inductively coupled plasma optical emission spectroscopy (ICP-OES) was used to validate elemental composition of Pd and S. Briefly, 50 mg of catalyst mass was loaded into polyethylene bottles for dissolution. Aqua regia was prepared by first adding 3.5 g HCl (Sigma-Aldrich, ACS reagent, 37 wt.%) then 2 g HNO_3_ (Sigma-Aldrich, Reagent grade, 70 wt.%) sequentially. Following this, 2.3 g HF (Fisher Chemical, TraceMetal grade, 48 wt.%) was added, and samples were allowed to sit in the acid solution for ∼24 h. After this, 50 g Milli-Q water was added to dilute the acid to <2 wt.% HF. These diluted samples were analyzed using an Agilent 5110 VDV Spectrometer. For calibrations, 1000 mg/L ICP standards (Fluka) were diluted with Milli-Q water to various concentrations.

CHNS elemental analysis was performed using an Elementar Vario EL Cube CHNS elemental analyzer operating with argon as the carrier gas to determine the elemental composition of S and N. For each analysis, 15–20 mg of catalysts were loaded into sample capsules. C, H, and N were detected using a thermal conductivity detector (TCD), while S was measured using an infrared (IR) detector. Three analyses were conducted per catalyst, and the average value was reported.

### 4.12 Reaction Kinetics Analysis Experiments of 4-Nitrophenyl 4-Hydroxybenzoate (4NP4HB) Hydrolysis

A 2 mM stock solution of substrate was prepared by dissolving 10 mg of 4NP4HB (Ambeed, 97.00%) in 20 mL of acetonitrile (ACN, Fisher Scientific, HPLC grade). A 5 mM solution of catalyst was freshly prepared by dissolving the corresponding amount of nanozyme in 5 mL of borate buffer at pH ∼ 8.4 (0.1 M boric acid (Sigma-Aldrich, BioReagent, ≥99.5%), 0.075 M NaCl (Sigma-Aldrich, ReagentPlus, ≥99.5%), and 0.025 M sodium tetraborate decahydrate (Sigma-Aldrich, ACS reagent, ≥99.5%) or by dissolving control compounds in 20 mL of borate buffer at pH ∼ 8.4. A 5 mL reaction feed (0.5 mM catalyst and 1.0 mM substrate in 50% v/v ACN cosolvent) was prepared by mixing 0.5 mL of catalyst solution, 2.0 mL of borate buffer, and 2.5 mL of stock 4NP4HB solution in a capped glass vial reactor. For reactions with different ACN compositions or initial 4NP4HB concentrations, the 5 mL feed solutions were prepared by adjusting the volumes of the catalyst solution, stock 4NP4HB solution, and borate buffer accordingly, or by using stock 4NP4HB solutions of different concentrations. For each feed configuration, a blank experiment conducted in the absence of a catalyst was performed by preparing a reaction mixture using the stock 4NP4HB solution and borate buffer to account for the background hydrolysis rate. After mixing, the reactor was heated in an oil bath at 50 °C and stirred at 600 rpm. Reactions conducted at room temperature were stirred on a magnetic plate without heating. At five distinct reaction times, 0.2 mL aliquots were withdrawn, diluted with 1.8 mL of borate buffer (10 times dilution by borate buffer), filtered through a 0.22 μm polytetrafluoroethylene (PTFE) syringe filter, and analyzed by UV–vis absorbance spectroscopy. The baseline of the UV–vis absorbance was established using a solution containing 0.05 mM catalyst in a cosolvent composed of 5% v/v ACN and 95% v/v borate buffer, in the absence of the 4NP4HB.

For heterogenized nanozyme catalyzed reactions, an 8 mL reaction feed (0.1/0.3 mM catalyst and 1.0 mM substrate in 50% v/v ACN cosolvent) was prepared by adding 4.0 mL of borate buffer, and 4.0 mL of stock 4NP4HB solution in a capped glass vial reactor with the corresponding amount of immobilized nanozyme powder. After mixing, the reactor was heated in an oil bath at 50 °C and stirred at 600 rpm. At five distinct reaction times, the glass vial reactor was centrifuged at 4000 rpm for 5 min. Subsequently, 0.2 mL aliquots were withdrawn, diluted with 1.8 mL of borate buffer, filtered through a 0.22 μm PTFE syringe filter, and analyzed by UV–vis absorbance spectroscopy. The baseline of the UV–vis absorbance was established using a solution containing 0.05 mM catalyst in a cosolvent composed of 5% v/v ACN and 95% v/v borate buffer, in the absence of the 4NP4HB.

For α-chymotrypsin catalyzed reactions, a solution of α-chymotrypsin (Chem-Impex, from bovine pancreas, lyophilized powder) was freshly prepared by dissolving the enzyme in borate buffer shortly before use. For each kinetic data point, a 2 mL reaction feed (catalyst to feed ratio of 0.1 in either 5% or 50% v/v ACN cosolvent) was prepared by combining α-chymotrypsin solution and stock 4NP4HB solution in a capped glass vial reactor. Reactions were conducted either at room temperature or at 50 °C in an oil bath. At five distinct reaction times, the 2 mL reaction solutions were filtered through a 0.2 μm surfactant-free cellulose acetate (SFCA) syringe filter equipped with a glass fiber prefilter to fully remove enzymes, and analyzed by UV–vis absorbance spectroscopy. For each sample, absorbance at 400 nm was recorded with borate buffer at pH ∼ 8.4 as the baseline.

The conversion of 4NP4HB, the yield of 4NP, and the carbon balance at a specific reaction time *t* for the hydrolysis of 4NP4HB into 4NP and 4HB were calculated using eqs 5–7.

5 $$\%\;\mathrm{Conversion}=\frac{{[4\mathrm{NP}4HB]}_{i}-{[4NP4HB]}_{t}}{{[4NP4HB]}_{i}}\times 100\%$$

6 $$\%\;\mathrm{Yield}=\frac{{[4NP]}_{t}}{{[4NP4HB]}_{i}}\times 100\%$$

7 $$\%\;\mathrm{Carbon balance}=\frac{13{[4NP4HB]}_{t}+6{[4NP]}_{t}+7{[4HB]}_{t}}{13{[4NP4HB]}_{i}}\times 100\%$$

### 4.13 UV–Vis Analysis

The UV–vis measurements were performed using a Thermo Scientific Evolution 300 UV–vis Spectrophotometer with a 10.0 mm path length quartz cuvette. For each sample, absorbance at 250, 320, and 400 nm was recorded, and full absorbance spectra were scanned from 240 to 600 nm. The calibration curves for concentration calculation are developed using commercial 4NP4HB, 4-hydroxybenzoic acid (Sigma-Aldrich, ReagentPlus, ≥99%), and 4-nitrophenol (Sigma-Aldrich, ReagentPlus, ≥ 99%).

The absorbances measured by the UV–vis spectrophotometer at 250, 320, and 400 nm, corresponding to the maximum absorbance wavelengths of 4HB, 4NP4HB, and 4NP, respectively, were used to calculate the concentrations. The baseline of the UV–vis absorbance was established using a solution containing the catalyst in a cosolvent composed of ACN and borate buffer at the same ratio as the kinetic sample solvent, but in the absence of 4NP4HB, to eliminate the absorbance contribution from the catalyst. Equations 8 and 9 were solved to determine the concentrations of 4NP4HB and 4NP, and eq 10 was then used to calculate the concentration of 4HB, in accordance with the Beer–Lambert Law.

8 $$AbS_{400}=C_{4NP,400}\left[4NP\right]+C_{4NP4HB,400}\left[4NP4HB\right]$$

9 $$AbS_{320}=C_{4NP,320}\left[4NP\right]+C_{4NP4HB,320}\left[4NP4HB\right]$$

10 $$AbS_{250}=C_{4NP,250}\left[4NP\right]+C_{4NP4HB,250}\left[4NP4HB\right]+C_{4HB,250}\left[4HB\right]$$

Abs represents the absorbance at a specific wavelength, and C denotes the calibration constant of the compound at that wavelength.

For the hydrolysis of 4NP4HB catalyzed by the enzyme α-chymotrypsin, the enzyme interfered with UV–vis absorbance, and its denatured form caused precipitation. Therefore, all kinetic samples were analyzed by UV–vis spectroscopy after enzyme removal, and only the measured concentrations of 4NP (400 nm) were used for reaction kinetics analysis. The concentration of 4NP4HB at specific reaction time points was estimated by subtracting the measured 4NP concentration from the initial 4NP4HB concentration.

### 4.14 Nanozyme Stability Assessment in Reaction Solvent

A 5 mM solution of catalyst was freshly prepared by dissolving the corresponding amount of nanozyme in 5 mL of borate buffer at pH ∼ 8.4. A 5 mL solution (0.5 mM catalyst in 50% v/v ACN cosolvent) was prepared by mixing 0.5 mL of catalyst solution, 2.0 mL of borate buffer, and 2.5 mL of ACN in a capped glass vial reactor. After mixing, the reactor was heated in an oil bath at 50 °C and stirred at 600 rpm for 16 to 18 h. Before and after the heating, 0.2 mL aliquots were withdrawn, diluted with 1.8 mL of borate buffer, filtered through a 0.22 μm PTFE syringe filter, and analyzed by UV–vis absorbance spectroscopy. UV–vis analysis was performed following the same procedure used for the homogeneous nanozyme samples with borate buffer at pH ∼ 8.4 as the baseline.

### 4.15 Simulation Protocols and Parameters

All-atom molecular dynamics simulations were performed using a leapfrog integrator with a time step of 2 fs. The temperature was maintained at 25 °C using a velocity-rescale thermostat^66^ with a coupling constant of 1.0 ps. The pressure was maintained at 1.0 bar with the compressibility set to 4.5 × 10^–5^ bar^–1^ using an isotropic C-rescale barostat with a coupling constant of 2.0 ps. Neighbor lists were constructed every 20 steps using the Verlet scheme and a cutoff of 1.2 nm. Van der Waals interactions were modeled using the cutoff scheme with the force switched to zero from 1.0 to 1.2 nm. Electrostatic interactions were modeled using the smooth Particle Mesh Ewald method with a short-range cutoff of 1.2 nm, Fourier spacing of 0.12 nm, and fourth-order interpolation. Hydrogen bonds were constrained using the LINCS algorithm. All interactions were described using the CHARMM36/CGenFF force field and the TIP3P water model.^67^ All simulations were performed with GROMACS, version 2021.5.^46^

Initial structures for the nanozymes and cosolvents were generated in Avogadro^68^ and subsequently used to obtain topology files from CGenFF.^69–71^ We performed independent simulations of water and acetonitrile at 25 °C to estimate their density in Figure S14 (1.008 g·cm^–3^ and 0.770 g·cm^–3^, respectively). The number of molecules were calculated with this density following experimental methods where mixtures were prepared at room temperature and reactions were performed at 50 °C. Each nanozyme was initially solvated in either a 50% or 25% v/v aqueous acetonitrile mixture. The number of molecules for each solvent was calculated for a (5.0 nm)^3^ box. One molecule of the nanozyme was solvated with one molecule of 4NP4HB and the cosolvents in a (6.2 nm)^3^ box to avoid steric clashes during system preparation. After equilibration, the box size fluctuated around (5.0 nm)^3^. The system was neutralized with Na^+^ counterions and 125 mM NaCl was added by replacing water molecules. This salt concentration was selected to match the borate buffer that was used experimentally to achieve a pH of 8.4. Energy minimization was performed for 5,000 steps or until the maximum force converged to 1,000 kJ·mol^–1^·nm^–1^. The system was equilibrated in the NVT ensemble for 100 ps and then in the NPT ensemble for 10 ns. Production simulations were performed in the NPT ensemble for 10 ns.

To study how 4NP4HB induces conformational changes to the nanozymes and how it modulates solvent effects, we slowly evolved the system along the center-of-mass (COM) distance between the nanozyme’s maleimide (NNZ_MAL_) group and 4NP4HB by using steered MD simulations as follows. First, from the production simulation we extracted a configuration with a $COM_{NNZ_{MAL}-4NP4HB}$ distance of 2.1 nm and applied a harmonic potential with a force constant of 50,000 kJ·mol^–1^·nm^–2^ to restrain these two groups at this distance. Next, we performed a 30 ns production simulation sampling every 1 ps. From the sampled configurations we extracted the configuration with the smallest $COM_{NNZ_{MAL}-4NP4HB}$ distance and performed a 2 ns steered MD simulation where we decreased this distance using a harmonic potential with a force constant of 50,000 kJ·mol^–1^·nm^–2^; the effective pull rate was 0.1 nm·ns^–1^. Such a slow pull rate was selected to minimize strong perturbations to the nanozyme’s conformations. Finally, the configuration with the $COM_{NNZ_{MAL}-4NP4HB}$ distance closest to 2.0 nm was extracted to perform a production simulation with the distance restrained. This algorithm was repeated to sample every 0.1 nm until a $COM_{NNZ_{MAL}-4NP4HB}$ distance of 0.2 nm was reached. This distance was chosen as the limit since it reaches the regime for reactive distances and the force applied was not sufficient to maintain numerical stability of the defined $COM_{NNZ_{MAL}-4NP4HB}$ distance (i.e., 0.2 nm is not reached) indicating steric overlap. This methodology is shown schematically in Figure S15. A total of 20 windows with $COM_{NNZ_{MAL}-4NP4HB}$ from 2.1 to 0.2 nm and a spacing of 0.1 nm were sampled. Six replicates were performed for each nanozyme at both 50% and 25% v/v acetonitrile. The number of molecules for each system is summarized in Table S3.

### 4.16 NH_δ_–O Hydrogen Bond Analysis

At each window, we calculated the distance between the nanozyme’s imidazole NH_δ_ and nearest carboxylate O (with the exception of FDM-GA-MAL) using the *gmx distance* tool with a bin width of 0.002 nm. These data generally yielded bimodal probability distribution functions as shown in Figure S16. We proceeded to decompose each distribution into two individual modes, classified as either a “closed” or “open” state. The closed state corresponds to conformations with hydrogen bonding between the imidazole NH_δ_ and nearest carboxylate O, causing these two groups to be near each other as shown in Figure 5B. Since we were primarily interested in conformations where the imidazole NH_δ_ and nearest carboxylate O exhibited hydrogen bonding, we computed the closed state probability at each window in Figure S17 and found that the nanozymes are capable of assuming closed states regardless of the proximity of 4NP4HB. However, when 4NP4HB is nearby (i.e., short NH_δ_–O distances of less than 0.5 nm), these probabilities increase drastically. In the open state conformations, these groups do not hydrogen bond and are far from each other as shown in Figure S18. In some windows, the probability distribution was unimodal in favor of the open states; therefore, for these windows the closed state probability was set to 0% and the corresponding average NH_δ_–O distance from that replicate was excluded from the calculation (Figure 5A). We selected the windows with $COM_{NNZ_{MAL}-4NP4HB}$ distance of 2.0 and 0.3 nm as the simulations where 4NP4HB was far from and near the nanozyme, respectively, for further analyses.

### 4.17 Peak Decomposition for Bimodal Distributions in NH_δ_–O Hydrogen Bond Analysis

The NH_δ_–O distances were binned from 0.0 to 1.0 nm using 0.01 nm-wide bins. These data were used to estimate kernel densities using Gaussian kernels with Silverman’s method to estimate the bandwidth factor, resulting in a smoothed probability distribution function. Relative extrema were calculated with an order of 3 to identify the number of modes. If multiple modes were detected, k-means clustering for two clusters was applied to identify the true mode which would correspond to the local maxima (of counts) within each cluster. Finally, smoothed bimodal distributions were split at the local minimum of the pit between the two modes, yielding the NH_δ_–O distance that separates open and closed states. The closed state probability was then calculated using the original probability distributions by integrating the closed state probability distribution and normalizing by the integral of the full bimodal distribution.

### 4.18 PETase Simulations

Simulations for PETase were performed following a previous study.^31^ To simulate the “near” system, as shown in Figure 5A and B, the initial configuration for the PETase with a hydroxyethyl-capped PET dimer bound at the catalytic triad active site was obtained using the CHAMBER utility of ParmEd 4.3.0.^72^ The system contained 16623 water molecules, 34 chloride counterions, and 27 sodium counterions. Because the system was extracted from an equilibrated QM/MM simulation, the same acylation reaction coordinate as described^31^ was used to restrain the catalytic triad at the active site during a 50 ns classical MD production simulation in the NPT ensemble. The PLUMED^73^ package, version 2.8.0, was used to perform these simulations. To simulate the “far” system, as shown in Figure 5A, the same initial configuration used for the “near” system was used, but the PET dimer was deleted from the system. The equivalent NH_δ_–O distances for the catalytic triad were computed and the probability distributions are shown in Figure S19.

### 4.19 Calculation of the Preferential Hydration

The preferential hydration parameter, Γ*, was computed using eq 11 and quantifies whether water or a cosolvent is enriched at a specific distance from the molecule’s surface. A value greater than 1 indicates that the molecule is preferentially hydrated, whereas a value less than 1 indicates that the molecule is preferentially dehydrated, as shown schematically on Figure S20. Because it is a normalized ratio, changing the solvent composition of the mixture can result in an increase in the absolute local number of water molecules while resulting in a lower Γ* value. This descriptor has been previously used to demonstrate that polyol molecules form a thin layer of water around the contour of protein surfaces.^50,51^ In these studies, the preferential hydration was defined as the normalized ratio of water oxygen atoms and was based on the oxygen atoms of the polyol molecules, we thus adapted it to use the carbon atom of the acetonitrile cyano group instead.

11 $$\Gamma^{*}=\frac{n_{W}^{L}\left(n_{W}^{T}+n_{C}^{T}\right)}{n_{W}^{T}\left(n_{W}^{L}+n_{C}^{L}\right)}$$

We calculated Γ* for our nanozymes by calculating the local number of cosolvent molecules ( $n_{W}^{L}$ for water and $n_{C}^{L}$ for acetonitrile) at a minimal distance r from each nanozyme atom. The total number of cosolvent molecules ( $n_{W}^{T}$ for water and $n_{C}^{T}$ for acetonitrile) is also accounted for to obtain a normalized value. Only the water’s oxygen atom and the acetonitrile’s central carbon atom (cyano group) that are found within a spherical shell of 0.1 nm width centered around each nanozyme atom are counted. These criteria result in 0.1 nm-thick 3D shells shaped to the nanozyme’s contour that allow us to quantify the hydration (or dehydration) at specific distances for nonspherical geometries. Figure S20 illustrates how Γ* is calculated.

In Table S5, we calculated the average number of water and acetonitrile molecules within the 0.3, 0.4, and 0.5 nm shells for systems at 50% and 25% v/v acetonitrile to show that decreasing the acetonitrile content leads to an increase in water molecules in the local environment but decreases the preferential hydration after normalizing by the total number of solvent molecules in the mixture, as shown in Figure 6C and D.

## Supplementary Material





## Data Availability

A Dryad repository with the simulation data and scripts to reproduce the computational methods has been published – DOI https://doi.org/10.5061/dryad.r2280gbrg.

## Notes

Complete reaction pathways and NMR spectra of all nanozymes; CHNS elemental analysis; schematic depiction of dithiol SAM; UV–vis analysis; details of reaction kinetics analysis of nanozymes; solvent density calculations from MD simulations; MD simulation system details; hydrogen bond analysis details; preferential hydration calculation details; tabulated results of solvent effect kinetic study; reaction kinetic analysis for α-Chymotrypsin (PDF)
