# Supplementary material for: Catalytic Triad-Inspired Nanozyme Catalysts for Ester Hydrolysis in Organic Solvent Mixtures
Source: ACS Catal. 2026 Mar 27;16(8):7506–21. doi: 10.1021/acscatal.5c09181 (PMC13097140; doi:10.1021/acscatal.5c09181)
Supplement: Supplementary file 2 [file cs5c09181_si_002.pdf]

Supplementary Information for

Catalytic Triad-Inspired Nanozyme Catalysts for Ester Hydrolysis in Organic Solvent Mixtures

Authors

Hoya Ihara[1], Carlos A. Huang-Zhu[1], Tianwei Yan[1], Matthew D. Edgar[1], Siddarth H. Krishna[1], Reid C. Van Lehn[1,2], James A. Dumesic[1], and George W. Huber[1*]

Affiliations

[1]Department of Chemical and Biological Engineering, University of Wisconsin-Madison; Madison, WI 53706, USA.

[2]Department of Chemistry, University of Wisconsin-Madison; Madison, WI 53706, USA.

[*]Corresponding author. Email: gwhuber@wisc.edu

Table of Contents

Supplementary Figures S1-S21

Supplementary Tables S1-S6

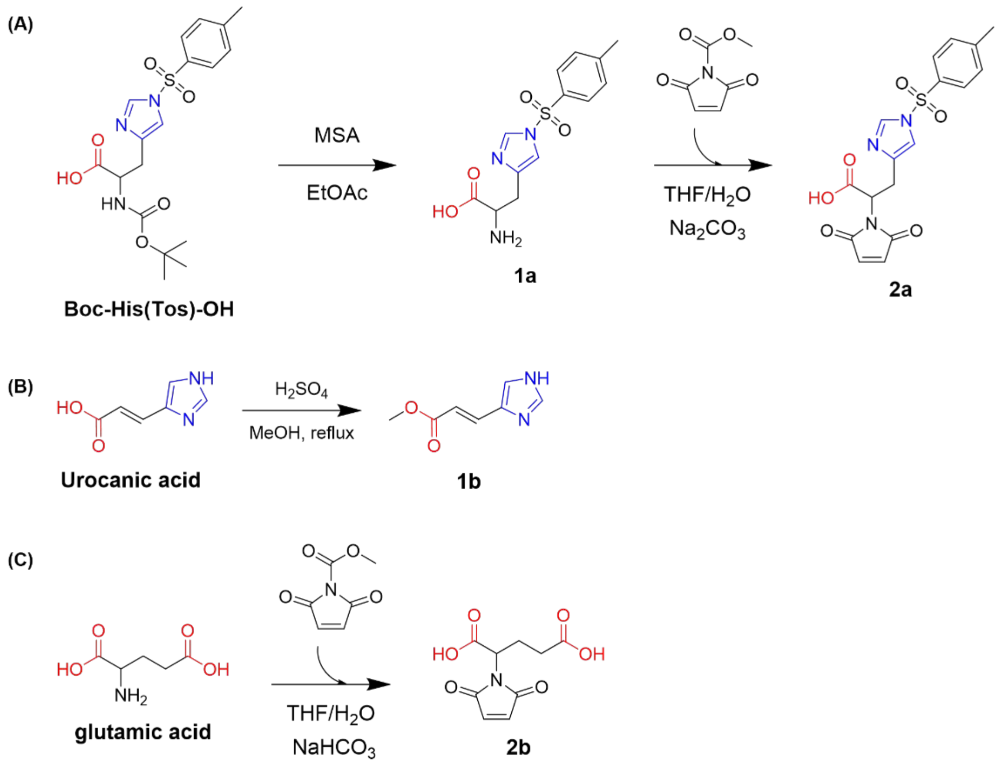

Figure S1. Reaction pathways for the synthesis of (**A**) histidine-appended maleimide with tosyl protection, (**B**) urocanic acid with methyl protection glutamic acid-appended maleimide, and (**C**) glutamic acid-appended maleimide.

(A)

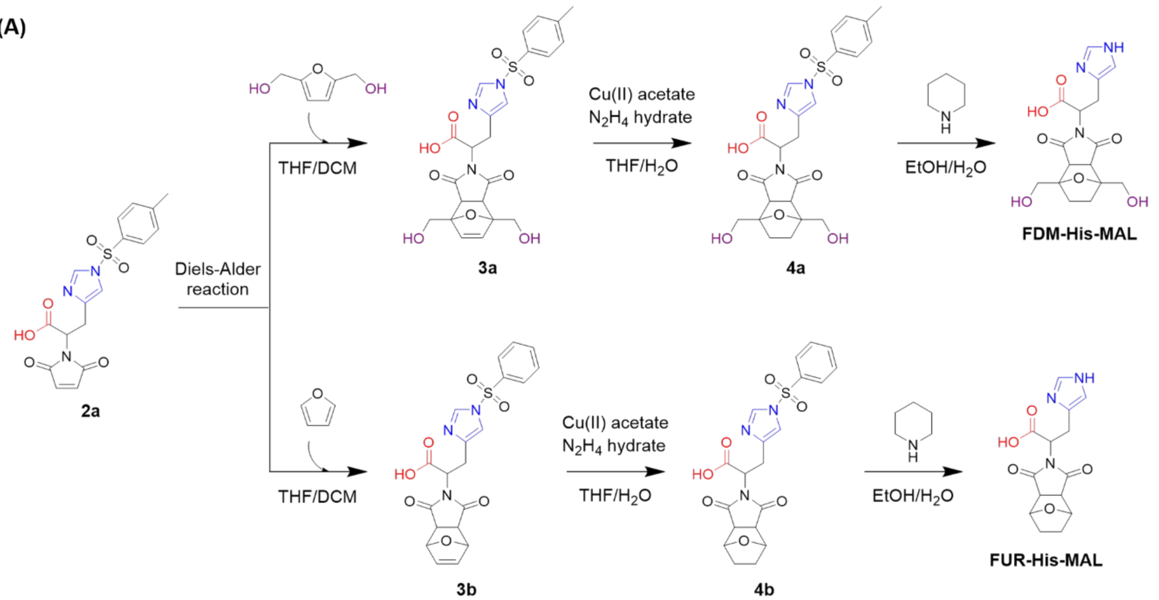
Diels-Alder reaction
THF/DCM
THF/DCM
Cu(II) acetate
N2H4 hydrate
THF/H2O
EtOH/H2O
2a
3a
3b
4a
4b
FDM-His-MAL
FUR-His-MAL

(B)

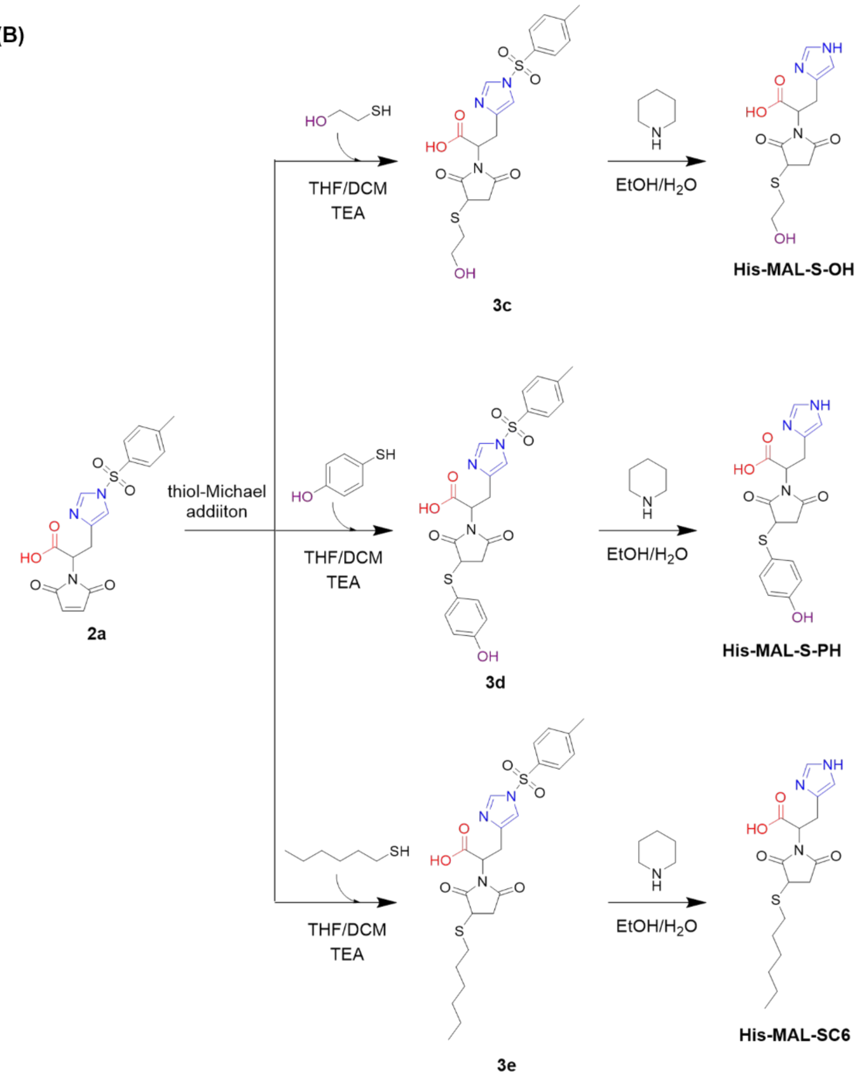
thiol-Michael addiiton
THF/DCM
TEA
EtOH/H2O
2a
3c
3d
3e
His-MAL-S-OH
His-MAL-S-PH
His-MAL-SC6

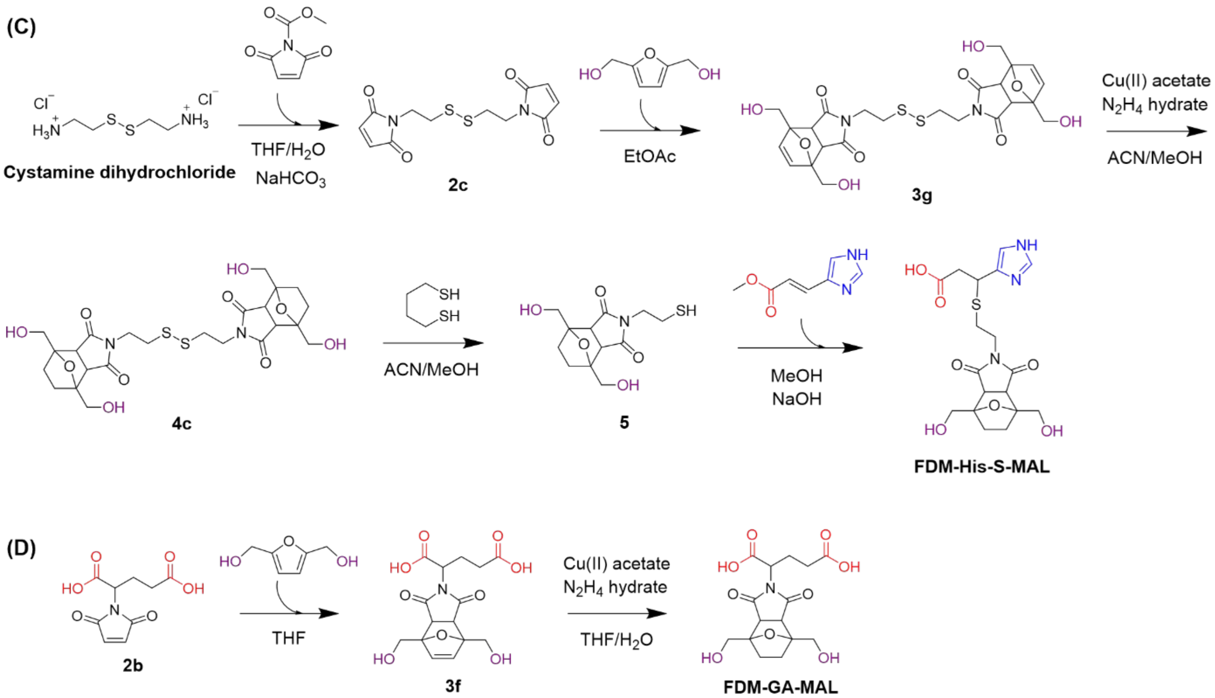

Figure S2. Reaction pathways for the synthesis of (**A**) nanozymes via Diels-Alder reaction using protected histidine-appended maleimide, (**B**) nanozymes via thiol-Michael addition using protected histidine-appended maleimide, (**C**) nanozyme via both Diels-Alder reaction and thiol-Michael addition using urocanic acid, and (**D**) nanozyme via Diels-Alder reaction using glutamic acid-appended maleimide.

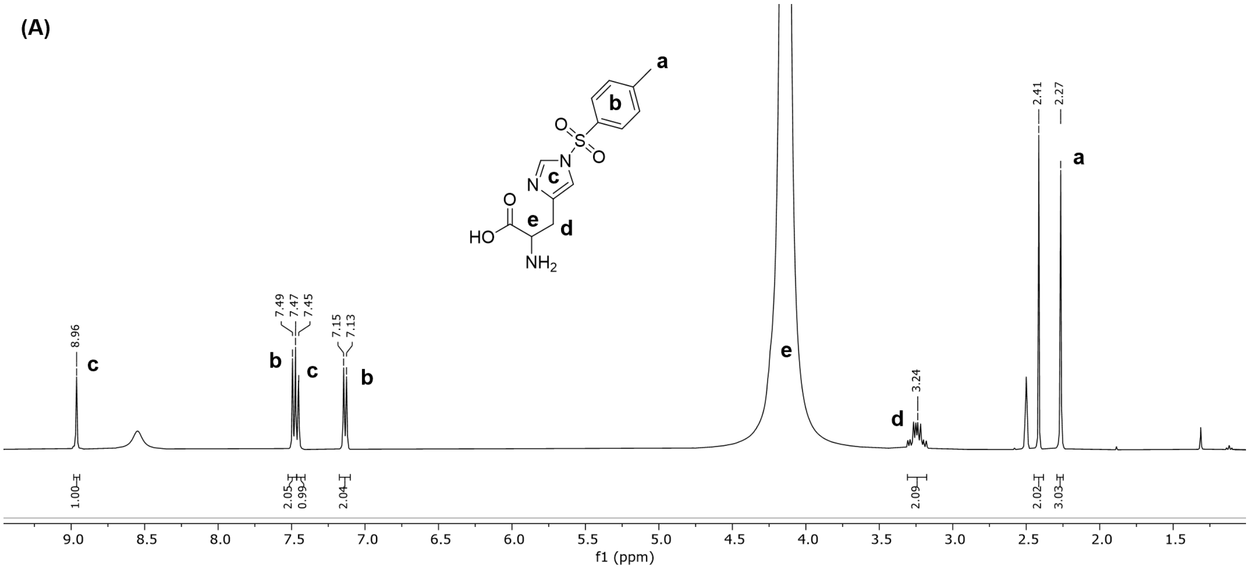

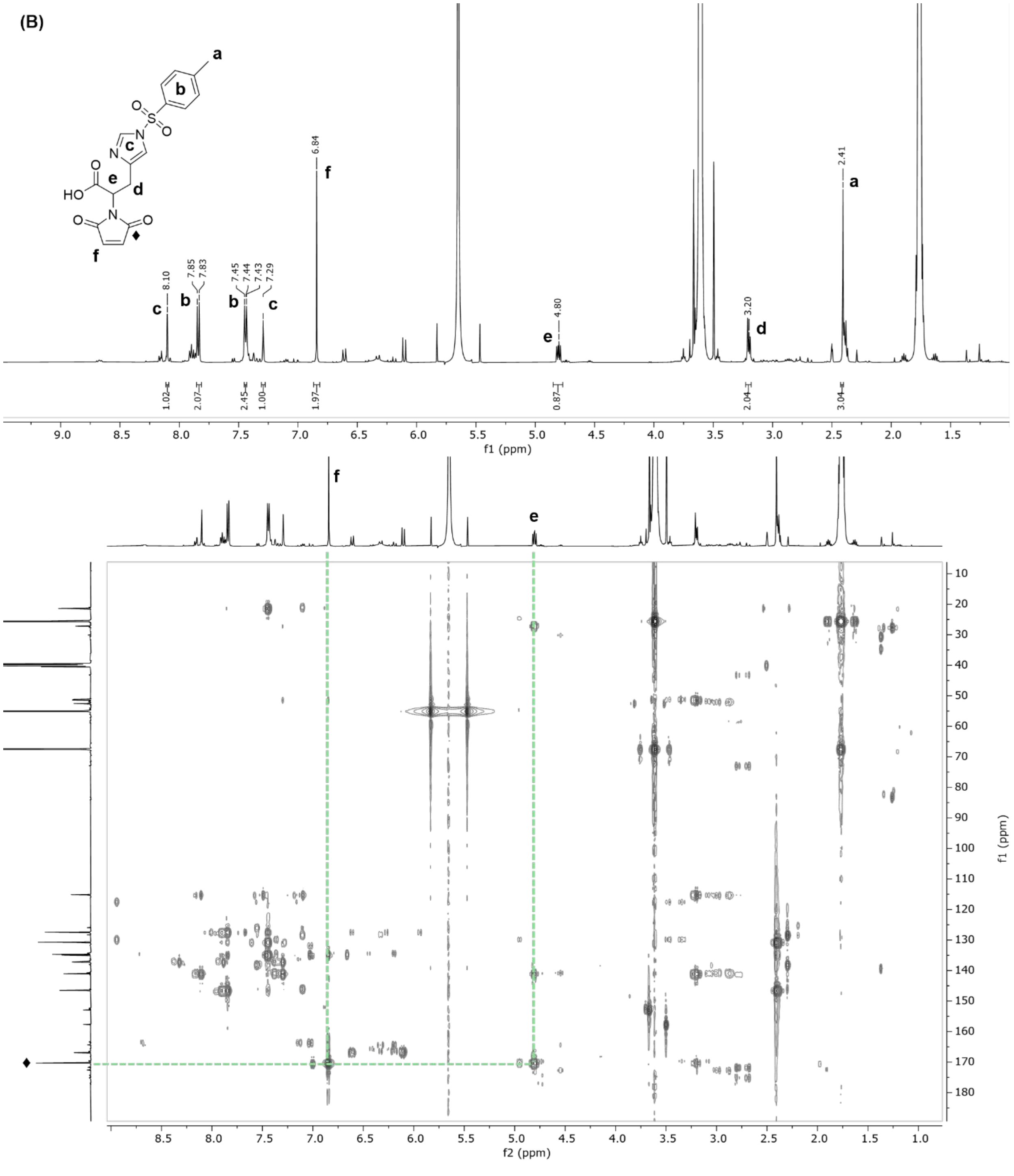
(B)
a
b
c
d
e
f
HO
O
N
S
8.10
7.85
7.83
7.45
7.44
7.43
7.29
6.84
4.80
3.20
2.41
1.02
2.07
2.45
1.00
1.97
0.87
2.04
3.04
f1 (ppm)
f2 (ppm)

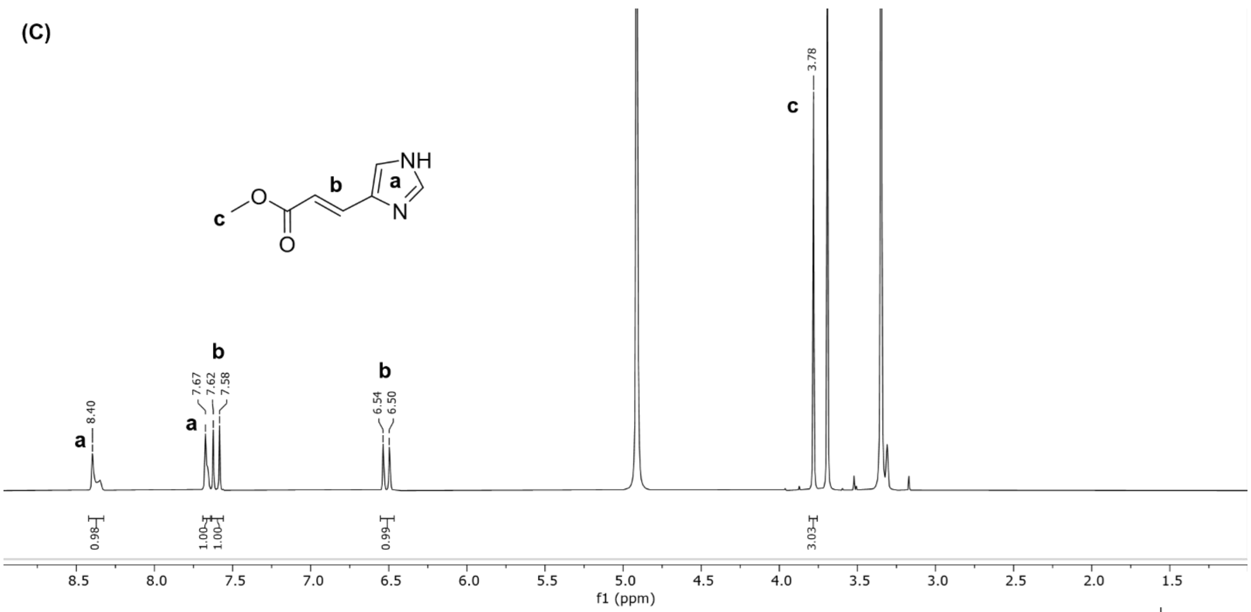
(C)
c
b
a
NH
N
O
O
a
b
a
b
c
8.40
7.67
7.62
7.58
6.54
6.50
3.78
0.98
1.00
1.00
0.99
3.03
8.5 8.0 7.5 7.0 6.5 6.0 5.5 5.0 4.5 4.0 3.5 3.0 2.5 2.0 1.5
f1 (ppm)

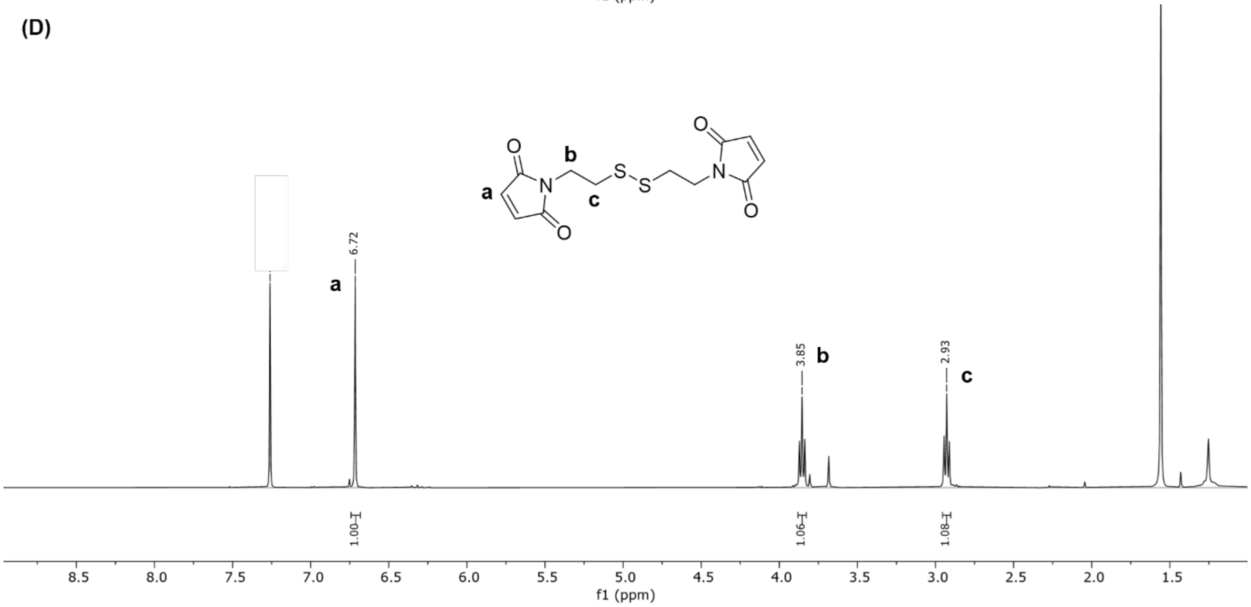
(D)
O
O
N
S
S
N
O
O
a
b
c
a
6.72
3.85
b
2.93
c
1.00
1.06
1.08
8.5 8.0 7.5 7.0 6.5 6.0 5.5 5.0 4.5 4.0 3.5 3.0 2.5 2.0 1.5
f1 (ppm)

(E)

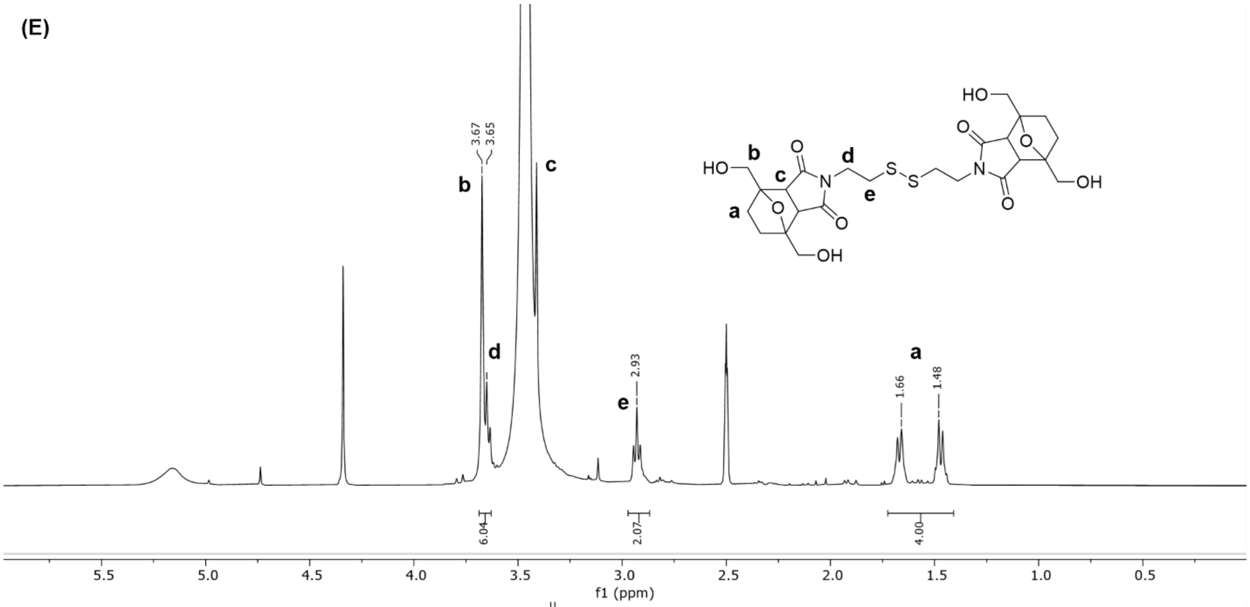
3.67
3.65
b
c
d
e
2.93
a
1.66
1.48
HO
OH
O
N
S
6.04
2.07
4.00
5.5
5.0
4.5
4.0
3.5
3.0
2.5
2.0
1.5
1.0
0.5
f1 (ppm)

(F)

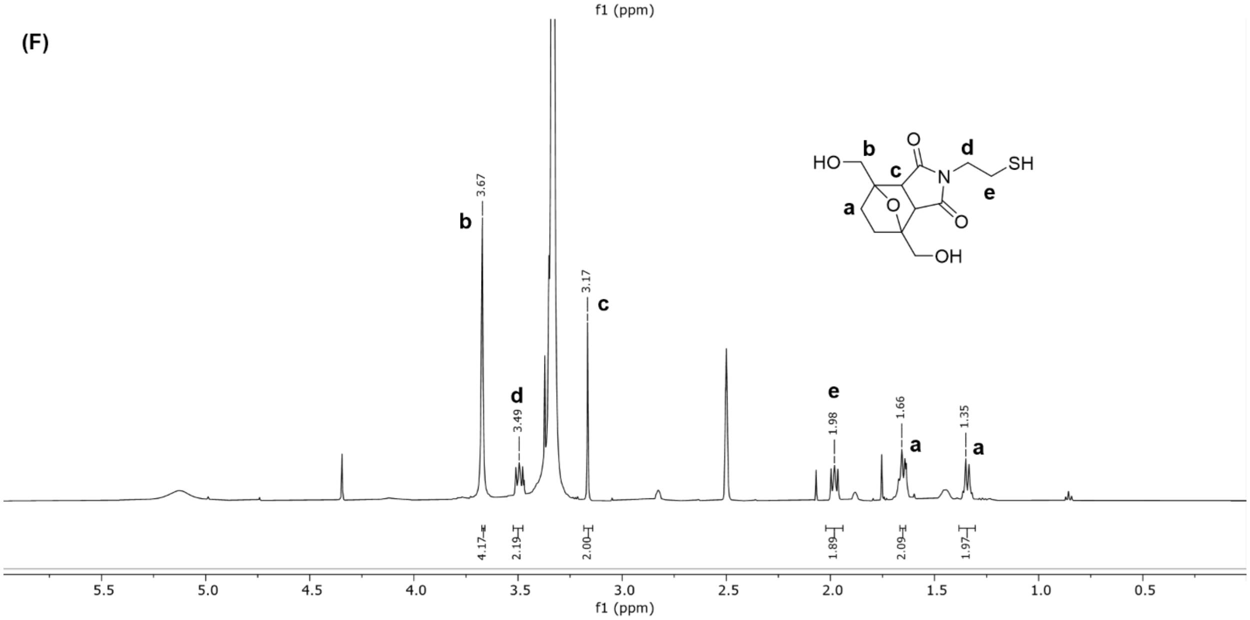
b
3.67
d
3.49
3.17
c
e
1.98
1.66
a
1.35
a
HO
OH
O
N
SH
4.17
2.19
2.00
1.89
2.09
1.97
5.5
5.0
4.5
4.0
3.5
3.0
2.5
2.0
1.5
1.0
0.5
f1 (ppm)

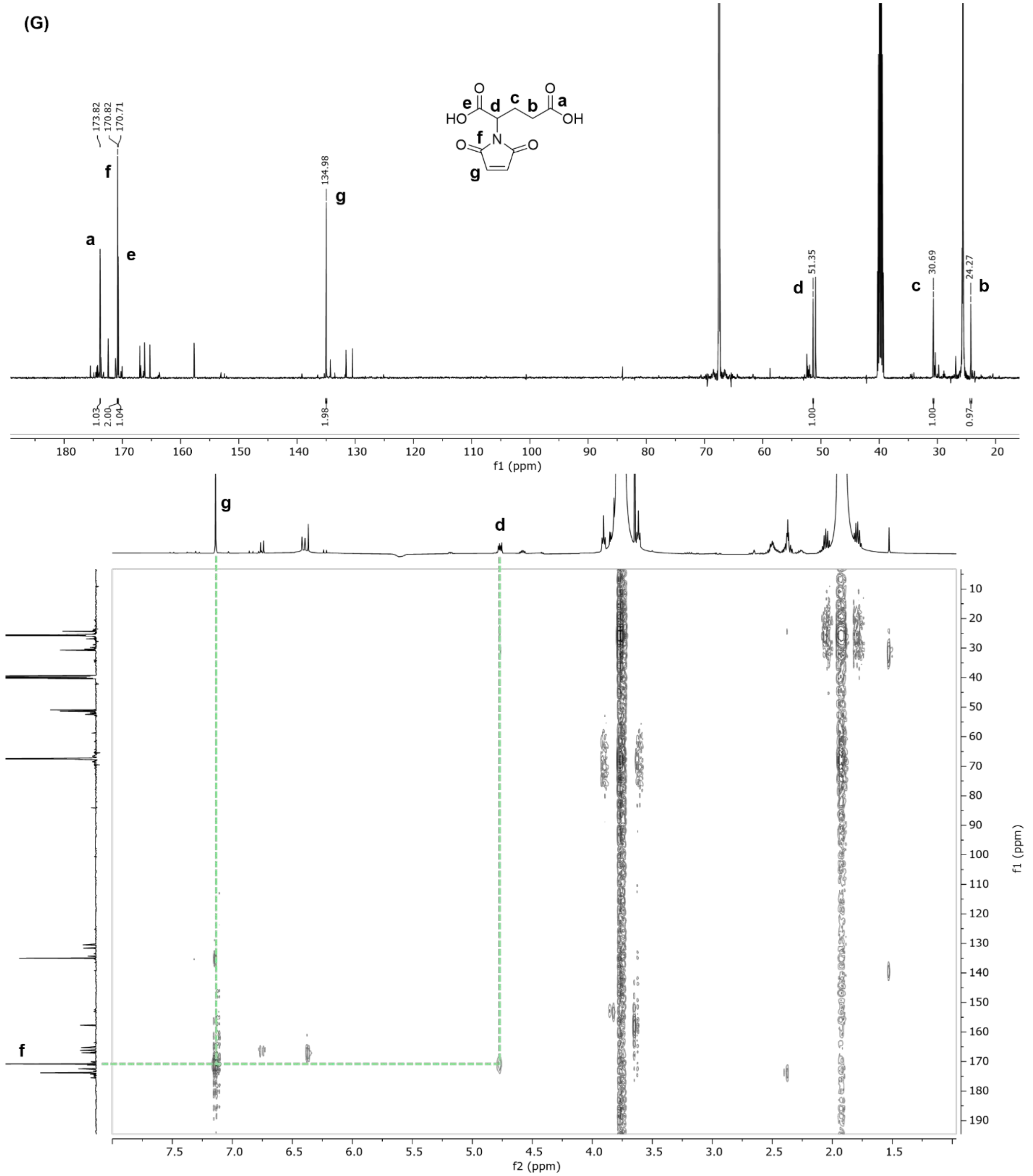

Figure S3. (**A**) ^{1}H NMR spectrum of compound **1a**. (**B**) ^{1}H and 2D HC HMBC NMR spectra of compound **2a**. (**C**) ^{1}H NMR spectrum of compound **1b**. (**D**) ^{1}H NMR spectrum of compound **2c**. (**E**) ^{1}H NMR spectrum of compound **4c**. (**F**) ^{1}H NMR spectrum of compound **5**. (**G**) ^{13}C and 2D HC HMBC NMR spectra of compound **2b**.

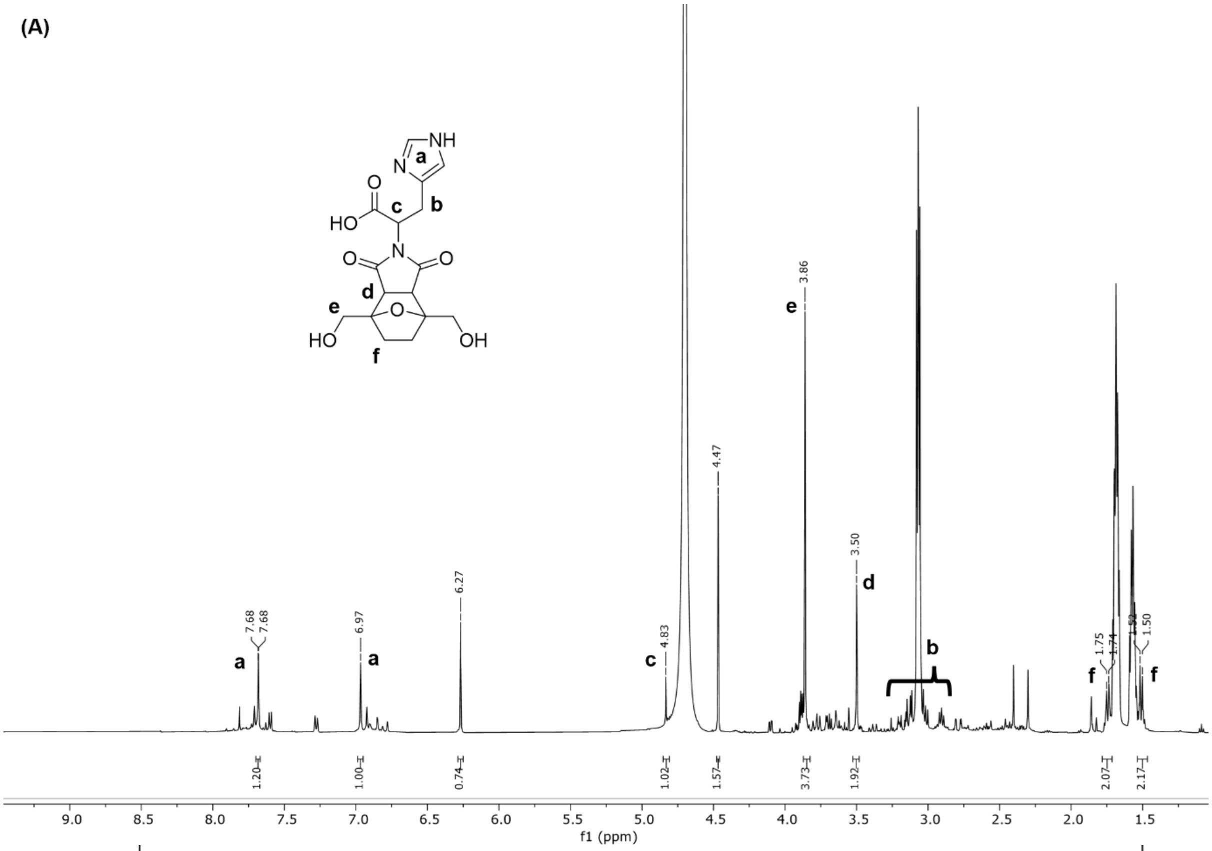
(A)
f1 (ppm)

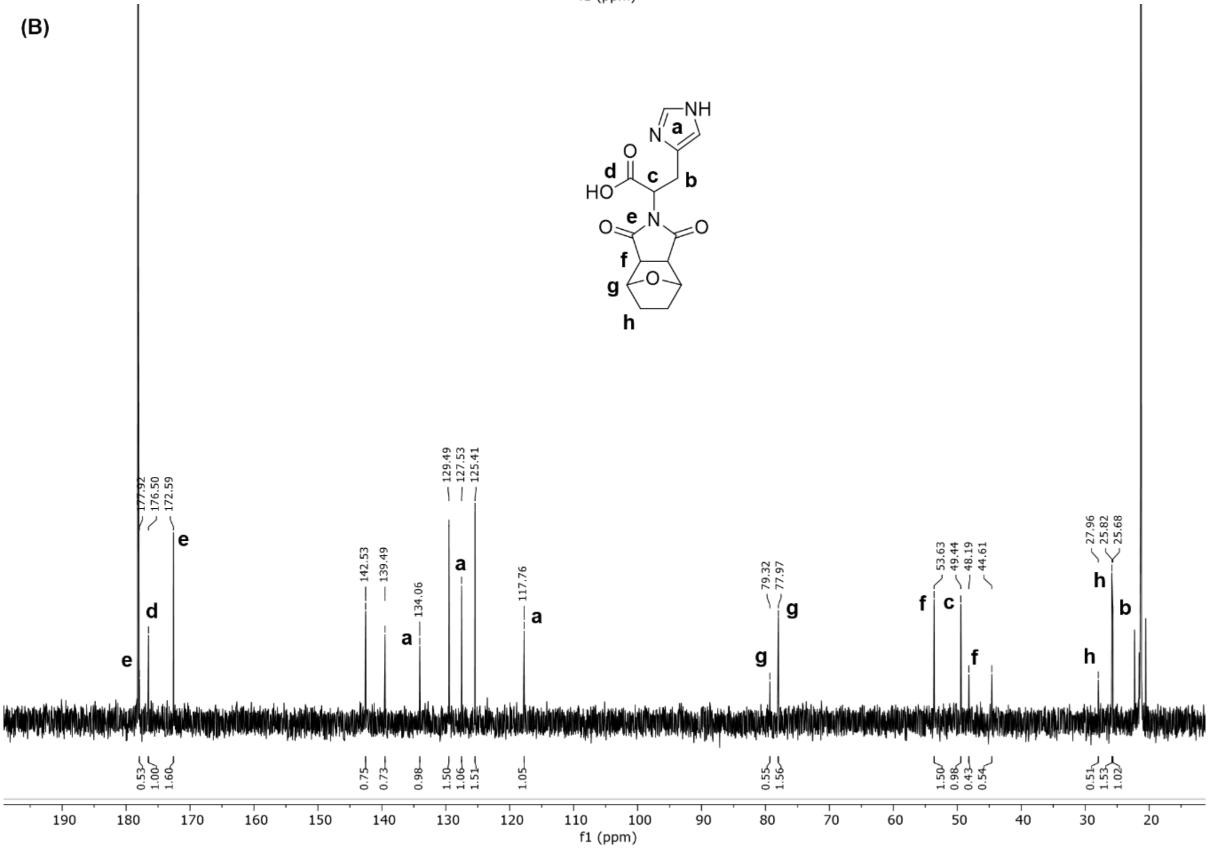
(B)
f1 (ppm)

(C)

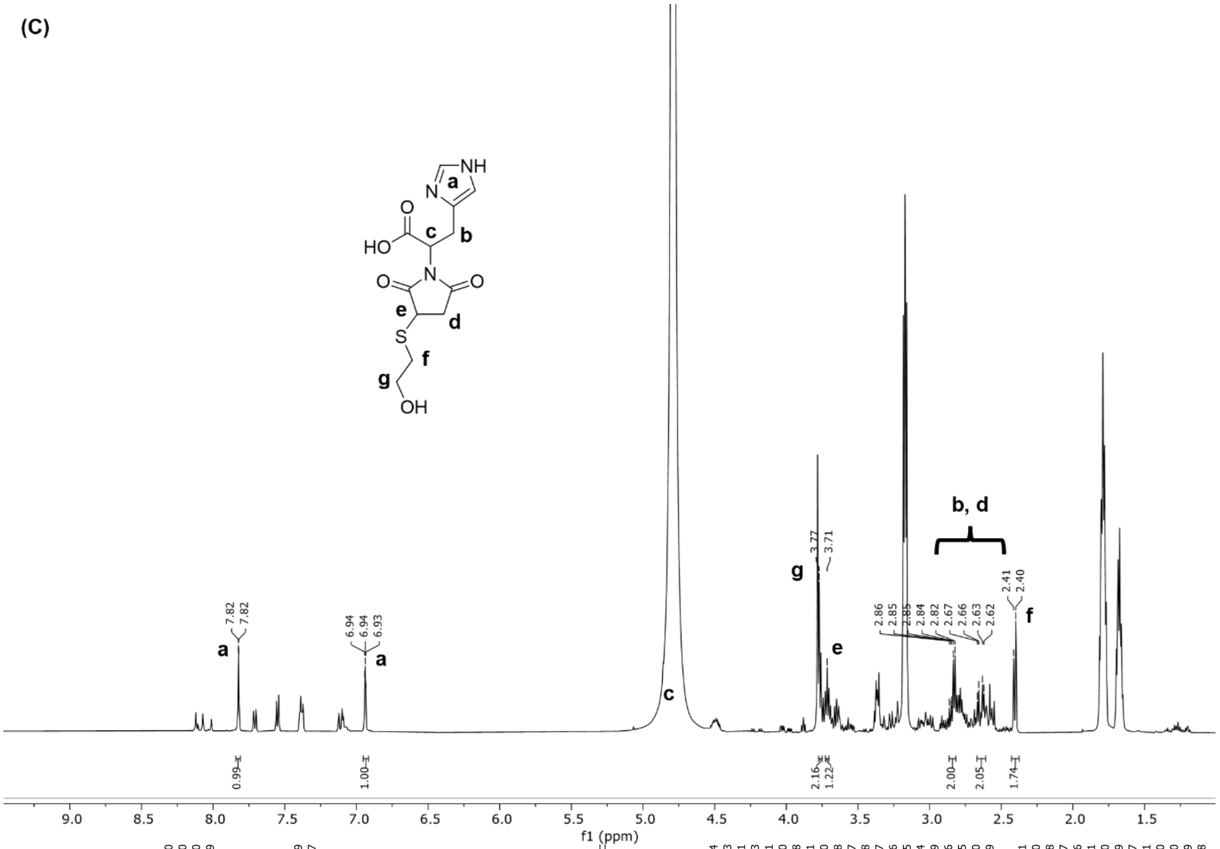

NH
a
N
O
HO
c
b
N
O
O
e
d
S
f
g
OH
b, d
g
3.77
3.71
e
a
7.82
7.82
6.94
6.94
6.93
2.86
2.85
2.85
2.84
2.82
2.67
2.66
2.63
2.62
2.41
2.40
f
c
0.99
1.00
2.16
1.22
2.00
2.05
1.74
f1 (ppm)

(D)

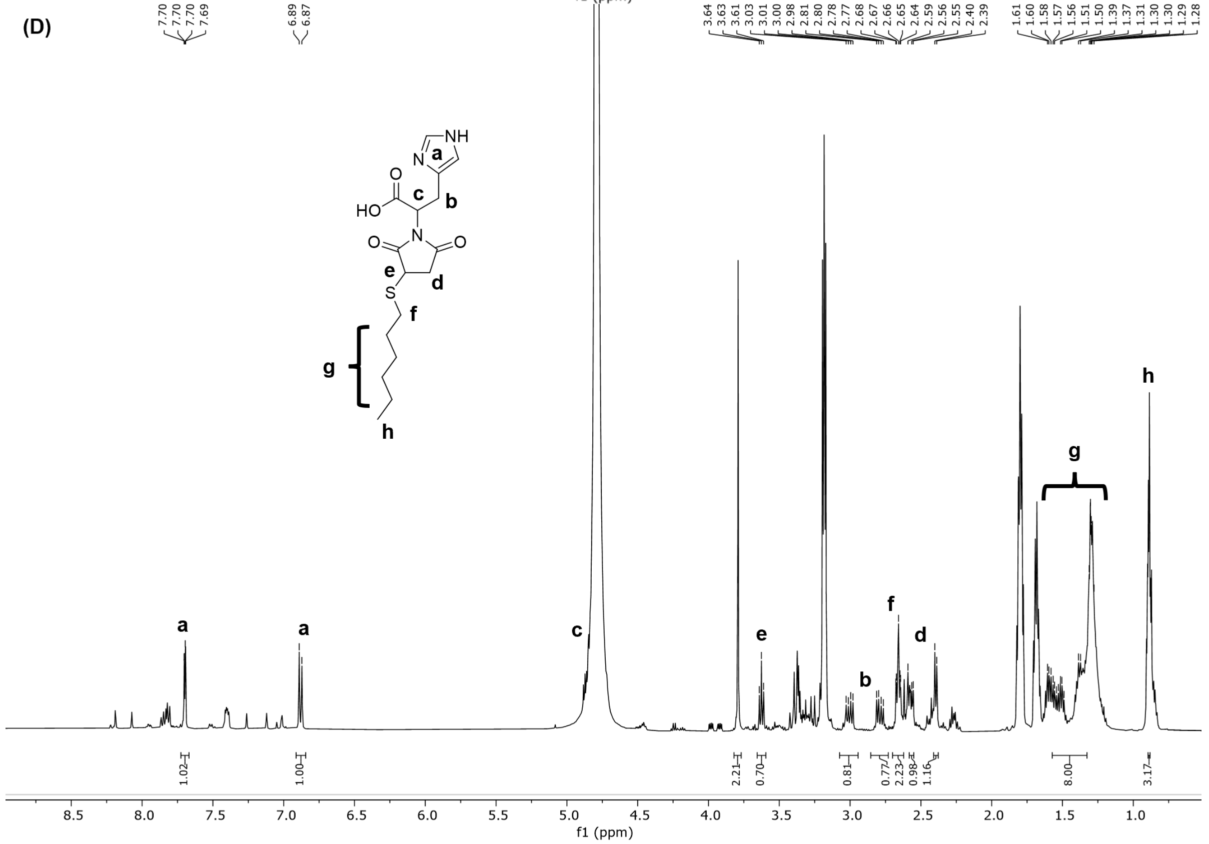

7.70
7.70
7.70
7.69
6.89
6.87
NH
a
N
O
HO
c
b
N
O
O
e
d
S
f
g
h
a
a
c
e
b
f
d
g
h
1.02
1.00
2.21
0.70
0.81
0.77
2.23
0.98
1.16
8.00
3.17
f1 (ppm)

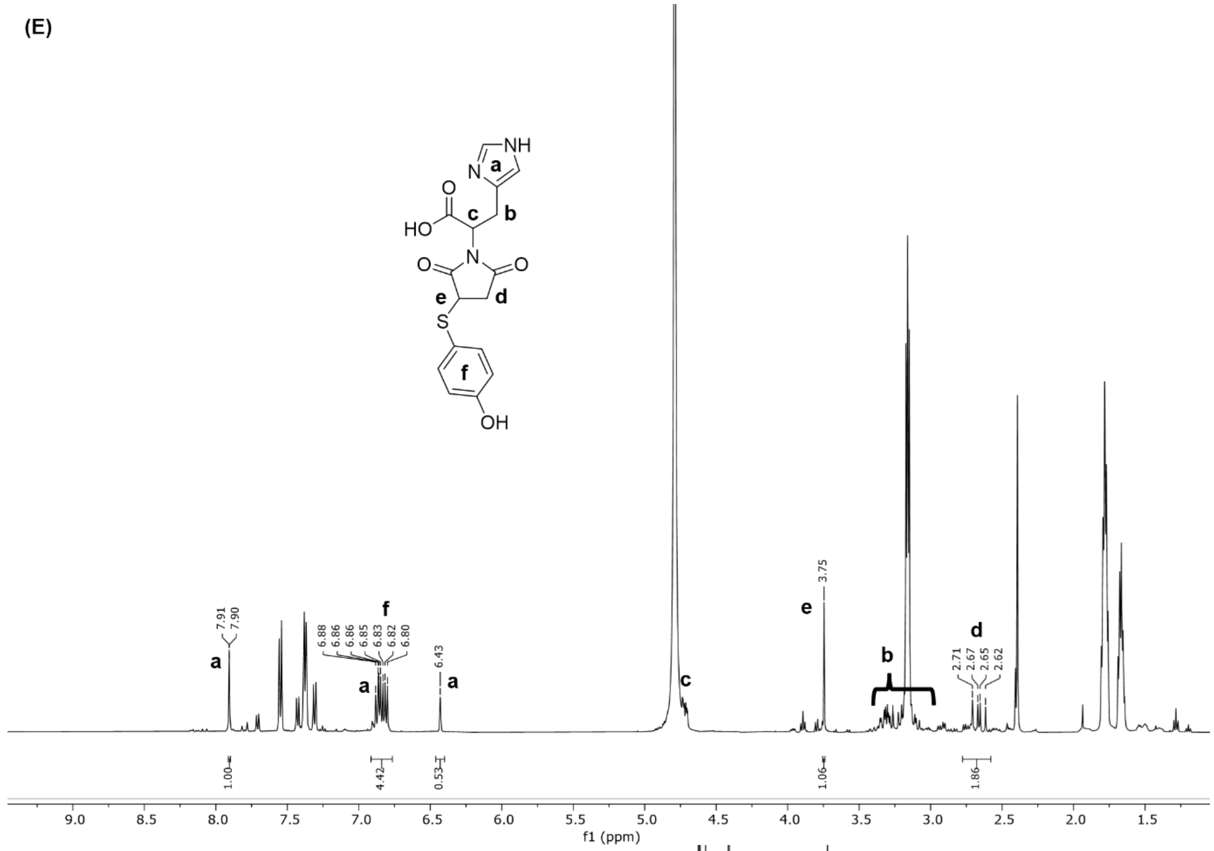
(E)
NH
N
a
O
HO
c
b
N
O
O
e
d
S
f
OH
7.91
7.90
6.88
6.86
6.86
6.85
6.83
6.82
6.80
6.43
3.75
2.71
2.67
2.65
2.62
1.00
4.42
0.53
1.06
1.86
9.0
8.5
8.0
7.5
7.0
6.5
6.0
5.5
5.0
4.5
4.0
3.5
3.0
2.5
2.0
1.5
f1 (ppm)

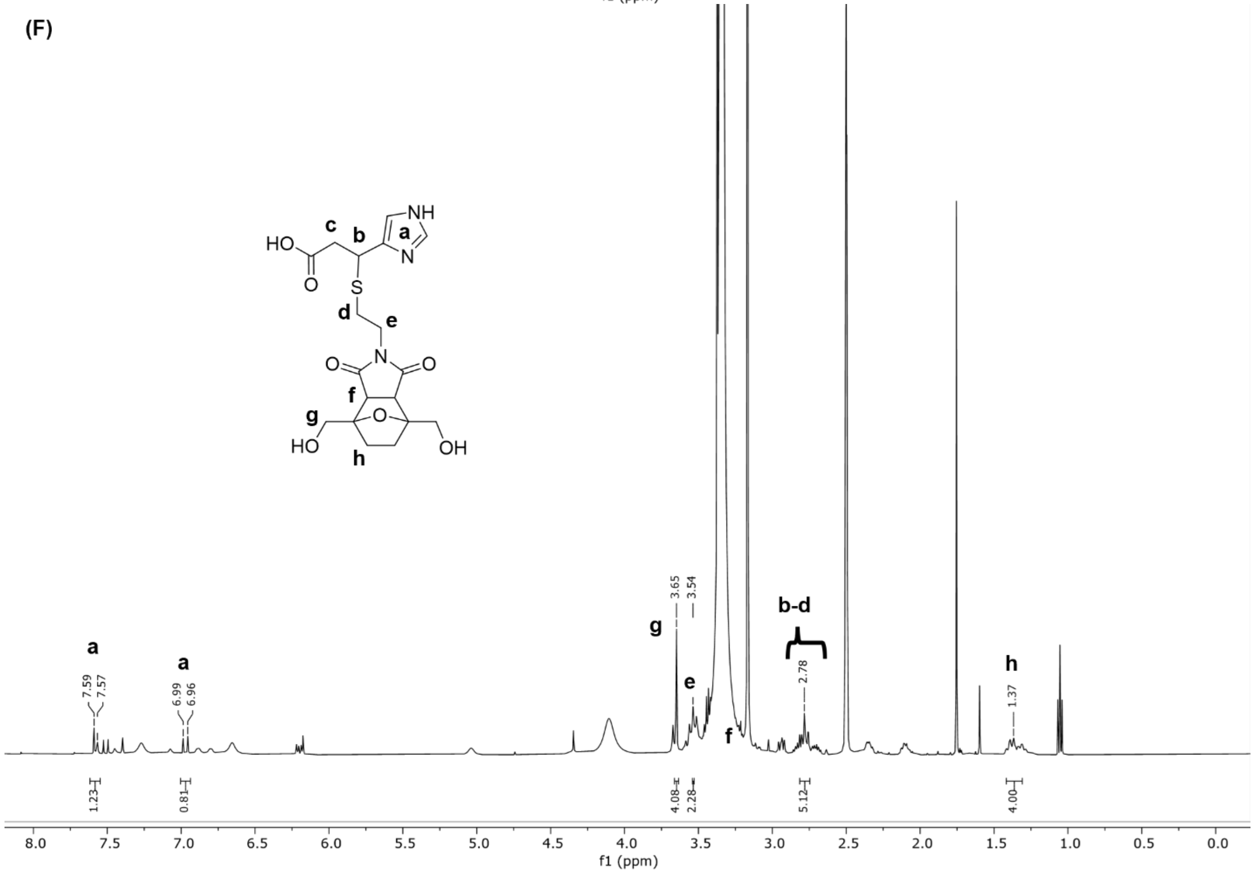
(F)
NH
HO
c
b
a
N
O
S
d
e
O
N
O
f
g
O
HO
OH
h
3.65
3.54
b-d
2.78
7.59
7.57
6.99
6.96
1.37
1.23
0.81
4.08
2.28
5.12
4.00
8.0
7.5
7.0
6.5
6.0
5.5
5.0
4.5
4.0
3.5
3.0
2.5
2.0
1.5
1.0
0.5
0.0
f1 (ppm)

(G)

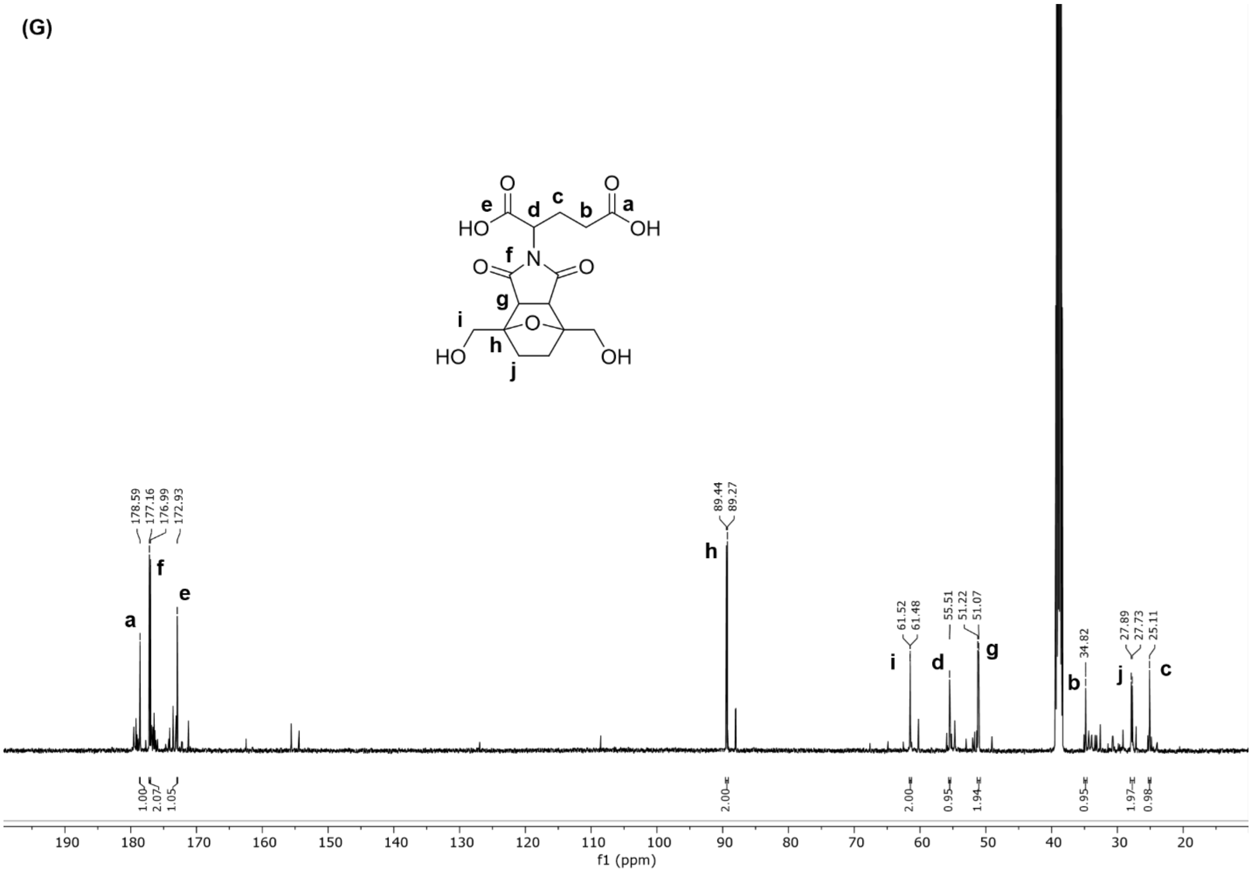

Figure S4. (**A**) ^{1}H NMR spectrum of FDM-His-MAL. (**B**) ^{13}C qNMR spectrum of FUR-His-MAL. (**C**) ^{1}H NMR spectrum of His-MAL-S-OH. (**D**) ^{1}H NMR spectrum of His-MAL-SC6. (**E**) ^{1}H NMR spectrum of His-MAL-S-PH. (**F**) ^{1}H NMR spectrum of FDM-His-S-MAL. (**G**) ^{13}C qNMR spectrum of FDM-GA-MAL.

Table S1. Quantified amounts of nitrogen (N) and sulfur (S) by CHNS elemental analysis on commercial Pd/SiO_2 without any modification, after dithiol SAM preparation, and after functionalization with FDM-His-MAL.

	N (wt%)	S (wt%)	Dithiol (mmol/g)	Imidazole (mmol/g)
Pd/SiO_2	0.000	0.025	0.004	0.000
1,6-hexaneditiol SAM on Pd/SiO_2	0.000	0.044	0.007	0.000
FDM-His-MAL-C6-Pd/SiO_2	0.313	0.062	0.010	0.075

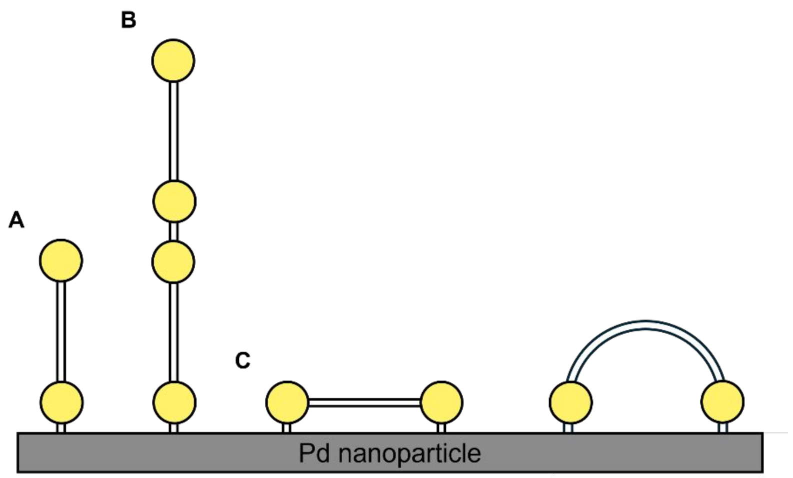

Figure S5. Schematic depctions of possible configurations of 1,6-hexanedithiol SAM; (**A**) ideal standing-up SAM, (**B**) multilayer SAM, in which one thiol end reacts with another via oxidation to form a disulfide bond, and (**C**) lying-down SAM, where both thiol ends adsorb to the metal surface.

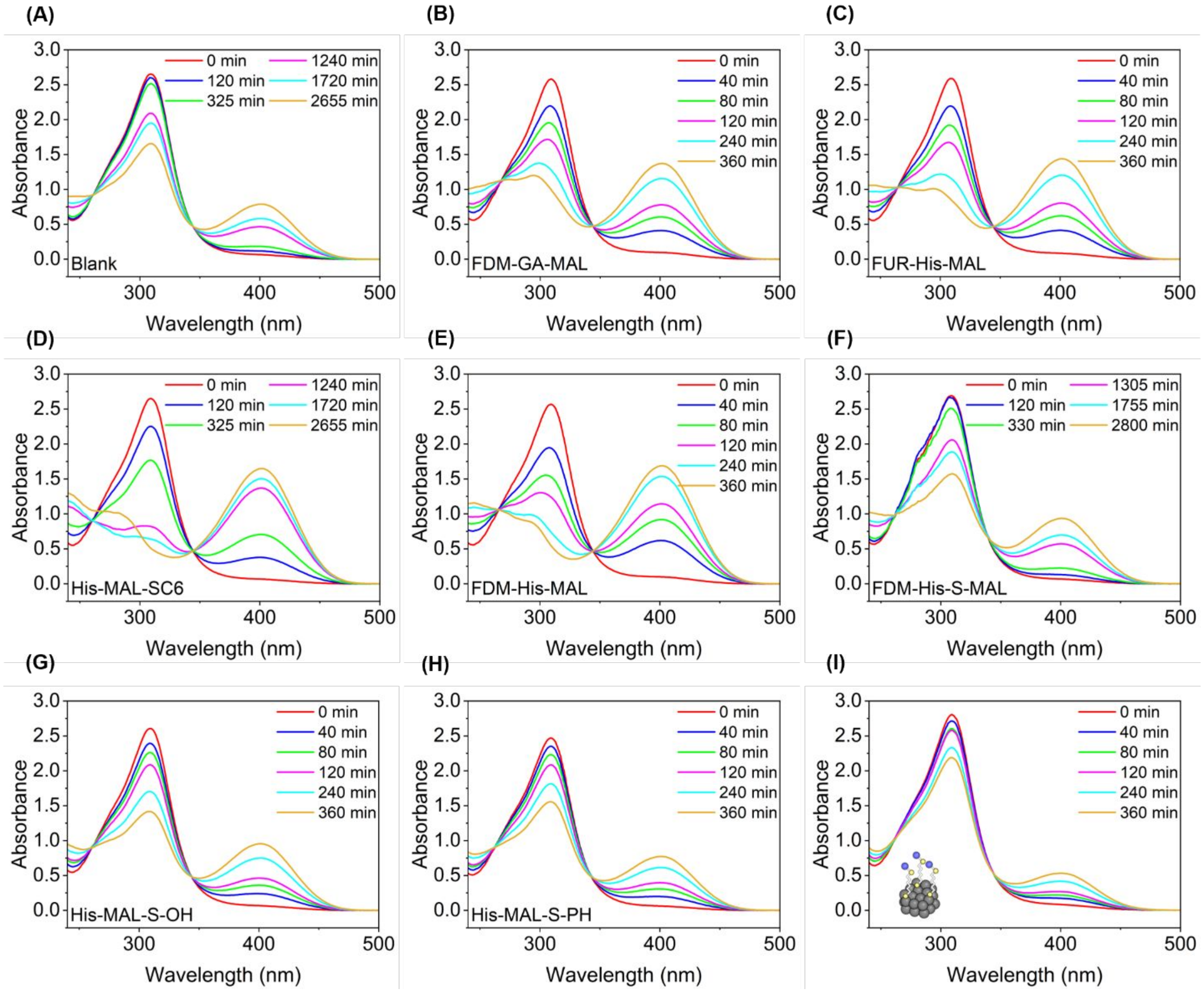

Figure S6. UV-vis absorbance spectrum of kinetic samples for reaction kinetics analysis of model ester hydrolysis at 50℃ in 50% acetonitrile and 50% borate buffer (pH 8.4) by volume with $[4NP4HB]_i$ of 1.0 mM and catalyst/feed of 0.5 catalyzed by (**A**) no catalyst (blank), (**B**) FDM-GA-MAL, (**C**) FUR-His-MAL, (**D**) His-MAL-SC6, (**E**) FDM-His-MAL, (**F**) FDM-His-S-MAL, (**G**) His-MAL-S-OH, and (**H**) His-MAL-S-PH, (**I**) FDM-His-MAL functionalized 1,6-hexanedithiol SAM on Pd/SiO_2 (catalyst/feed of 0.1).

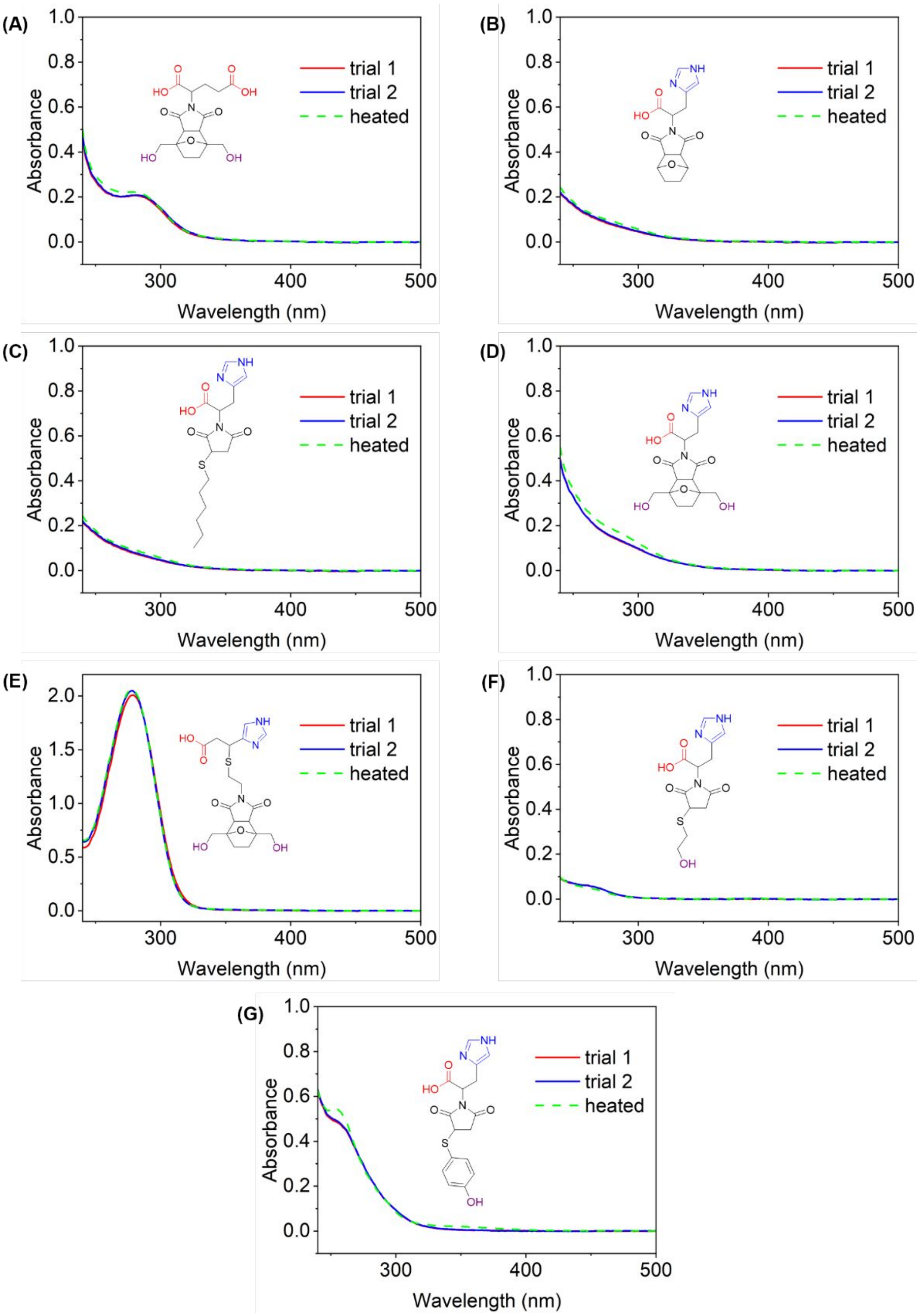

Figure S7. UV-vis absorbance spectrum of catalyst solution without 4NP4HB used as the baseline of UV-vis absorbance measurements for reaction kinetics analysis of model ester hydrolysis at 50℃ in 50% acetonitrile and 50% borate buffer (pH 8.4) by volume; and catalyst solution without 4NP4HB heated at 50°C in 50% acetonitrile and 50% borate buffer (pH 8.4) by volume for 16-18 h; (**A**) FDM-GA-MAL, (**B**) FUR-His-MAL, (**C**) His-MAL-SC6, (**D**) FDM-His-MAL, (**E**) FDM-His-S-MAL, (**F**) His-MAL-S-OH, and (**G**) His-MAL-S-PH.

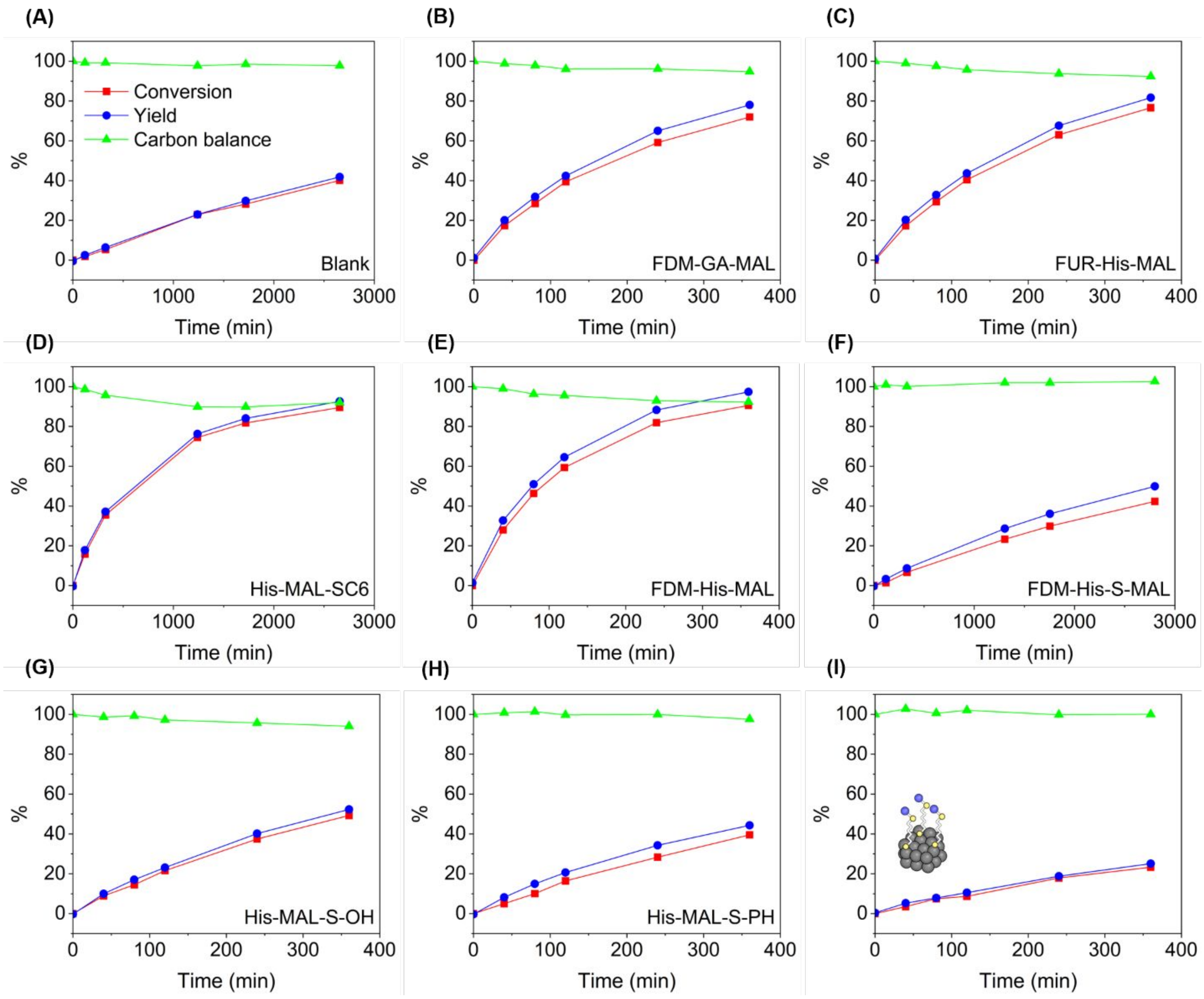

Figure S8. 4NP4HB conversion, 4NP yield and carbon balance of model ester hydrolysis at 50℃ in 50% acetonitrile and 50% borate buffer (pH 8.4) by volume with $[4NP4HB]_i$ of 1.0 mM and catalyst/feed of 0.5 catalyzed by (**A**) no catalyst (blank), (**B**) FDM-GA-MAL, (**C**) FUR-His-MAL, (**D**) His-MAL-SC6, (**E**) FDM-His-MAL, (**F**) FDM-His-S-MAL, (**G**) His-MAL-S-OH, and (**H**) His-MAL-S-PH, (**I**) FDM-His-MAL functionalized 1,6-hexanedithiol SAM on Pd/SiO_2 (catalyst/feed of 0.1).

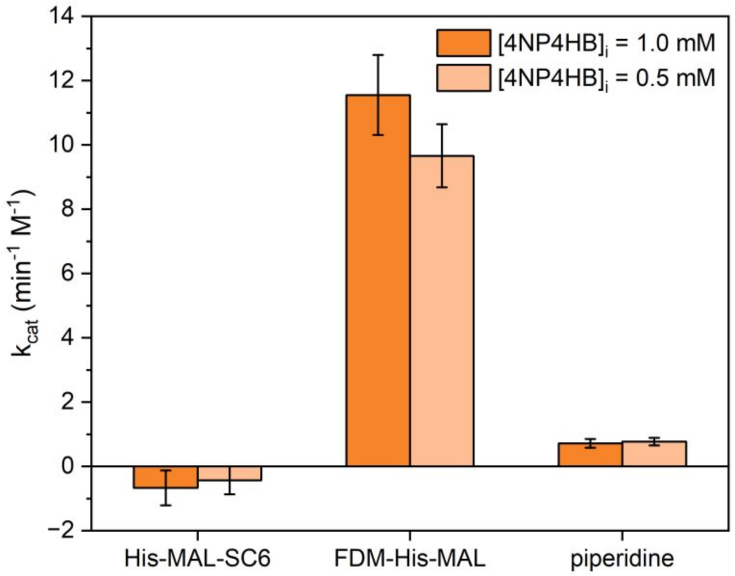

Figure S9. Normalized rate constant (k_{cat}) of His-MAL-SC6, FDM-His-MAL and piperidine for model ester hydrolysis at 50℃ in 50% acetonitrile and 50% borate buffer (pH 8.4) by volume with catalyst/feed of 0.5 at [4NP4HB]$_I$ of 1.0 and 0.5 mM. Error was computed as the 95% confidence interval from nonlinear fitting.

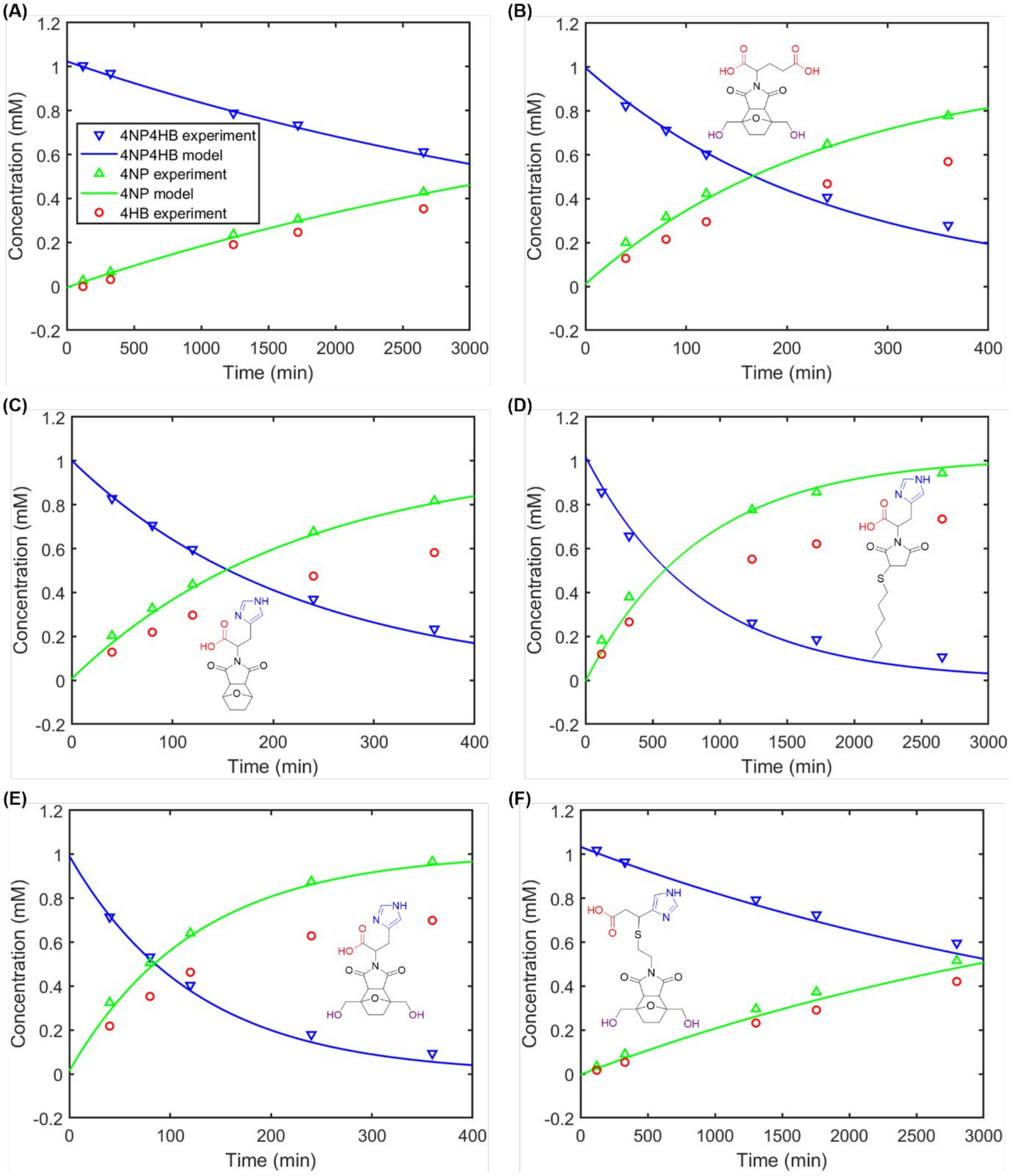
(A)
(B)
(C)
(D)
(E)
(F)
Concentration (mM)
Time (min)
4NP4HB experiment
4NP4HB model
4NP experiment
4NP model
4HB experiment
HO
OH
O
N
NH

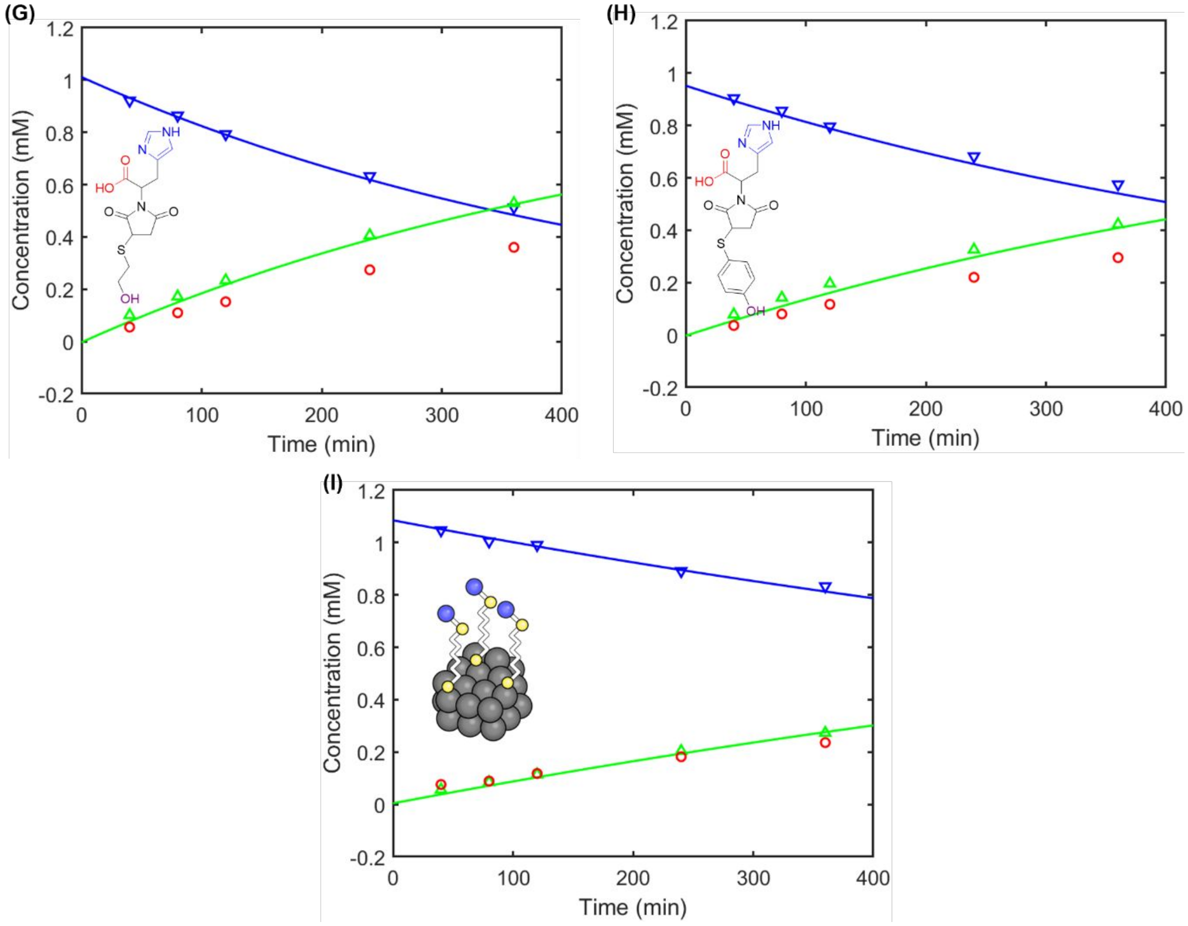

Figure S10. Experimental concentrations and kinetic model concentrations for reaction kinetics analysis of model ester hydrolysis at 50℃ in 50% acetonitrile and 50% borate buffer (pH 8.4) by volume with $[4NP4HB]_i$ of 1.0 mM and catalyst/feed of 0.5 catalyzed by nanozymes; (**A**) no catalyst (blank), (**B**) FDM-GA-MAL, (**C**) FUR-His-MAL, (**D**) His-MAL-SC6, (**E**) FDM-His-MAL, (**F**) FDM-His-S-MAL, (**G**) His-MAL-S-OH, and (**H**) His-MAL-S-PH, (**I**) FDM-His-MAL functionalized 1,6-hexanedithiol SAM on Pd/SiO_2 (catalyst/feed of 0.1).

Table S2. Summary of intrinsic catalytic activity of nanozymes, precursors, and amino acids for 4NP4HB hydrolysis at 50°C in 50% acetonitrile and 50% borate buffer (pH 8.4) by volume with $[4NP4HB]_i$ of 1.0 mM. Error was computed as the 95% confidence interval from nonlinear fitting.

Catalyst	Trial	Catalyst / feed	k_{app} (10^{-3} min^{-1})	k_{cat} ($min^{-1} \cdot M^{-1}$)	TOF (10^{-3} min^{-1})	TON
Blank	1	-	0.20 ± 0.01	-	-	-
	2	-	0.20 ± 0.01	-	-	-
Piperidine	1	0.5	0.51 ± 0.05	0.70 ± 0.12	0.71 ± 0.12	1.89
	2	0.5	0.50 ± 0.06	0.72 ± 0.14	0.75 ± 0.14	2.00
FDM-GA-MAL	1	0.5	4.10 ± 0.29	7.85 ± 0.59	7.83 ± 0.58	2.82
	2	0.5	3.50 ± 0.28	6.55 ± 0.55	6.68 ± 0.56	2.40
FUR-His-MAL	1	0.5	4.45 ± 0.23	5.94 ± 0.63	5.96 ± 0.63	2.14
	2	0.5	4.47 ± 0.28	6.06 ± 0.74	6.16 ± 0.75	2.22
His-MAL-SC6	1	0.5	1.16 ± 0.14	-0.46 ± 0.51	-0.47 ± 0.52	-1.24
	2	0.5	1.09 ± 0.14	-0.67 ± 0.54	-0.71 ± 0.57	-1.87
FDM-His-MAL	1	0.5	8.02 ± 0.52	11.82 ± 1.25	11.74 ± 1.24	4.23
	2	0.5	7.88 ± 0.50	11.55 ± 1.25	11.90 ± 1.28	4.28
	3	0.1	1.54 ± 0.06	9.27 ± 0.92	9.72 ± 0.96	3.50
FDM-His-S-MAL	1	0.5	0.23 ± 0.02	0.08 ± 0.06	0.08 ± 0.06	0.23
	2	0.5	0.19 ± 0.02	0.08 ± 0.06	0.08 ± 0.07	0.23
His-MAL-S-OH	1	0.5	2.05 ± 0.11	0.71 ± 0.56	0.72 ± 0.56	0.26
	2	0.5	2.04 ± 0.10	0.67 ± 0.61	0.70 ± 0.64	0.25
His-MAL-S-PH	1	0.5	1.57 ± 0.15	0.04 ± 0.57	0.04 ± 0.54	0.01
	2	0.5	1.56 ± 0.11	-0.07 ± 0.57	-0.07 ± 0.56	-0.03
FDM-His-MAL-C6-Pd/SiO_2	1	0.1	0.80 ± 0.05	5.03 ± 0.43	5.45 ± 0.47	1.96
	2	0.3	1.57 ± 0.16	4.22 ± 0.48	4.65 ± 0.53	1.68
Imidazole	1	0.5	0.16 ± 0.01	0.01 ± 0.02	0.01 ± 0.02	0.02
Glutamic acid (GA)	1	0.5	0.17 ± 0.01	0.03 ± 0.02	0.04 ± 0.02	0.10
Serine (Ser)	1	0.5	0.18 ± 0.02	0.06 ± 0.04	0.07 ± 0.04	0.18
Maleimide (MAL)	1	0.5	0.18 ± 0.01	0.05 ± 0.03	0.05 ± 0.03	0.15
Furandimethanol (FDM)	1	0.5	0.12 ± 0.01	-0.07 ± 0.03	-0.07 ± 0.03	-0.20
Histidine (His)	1	0.5	0.21 ± 0.02	0.04 ± 0.06	0.04 ± 0.07	0.12
p-toluenesulfonic acid (Tos)	1	0.5	0.17 ± 0.02	-0.04 ± 0.05	-0.04 ± 0.05	-0.11
His + Ser*	1	0.5	0.13 ± 0.01	-0.05 ± 0.02	-0.05 ± 0.02	-0.13

*Mixture of histidine and serine that [cat] = [His] + [Ser].

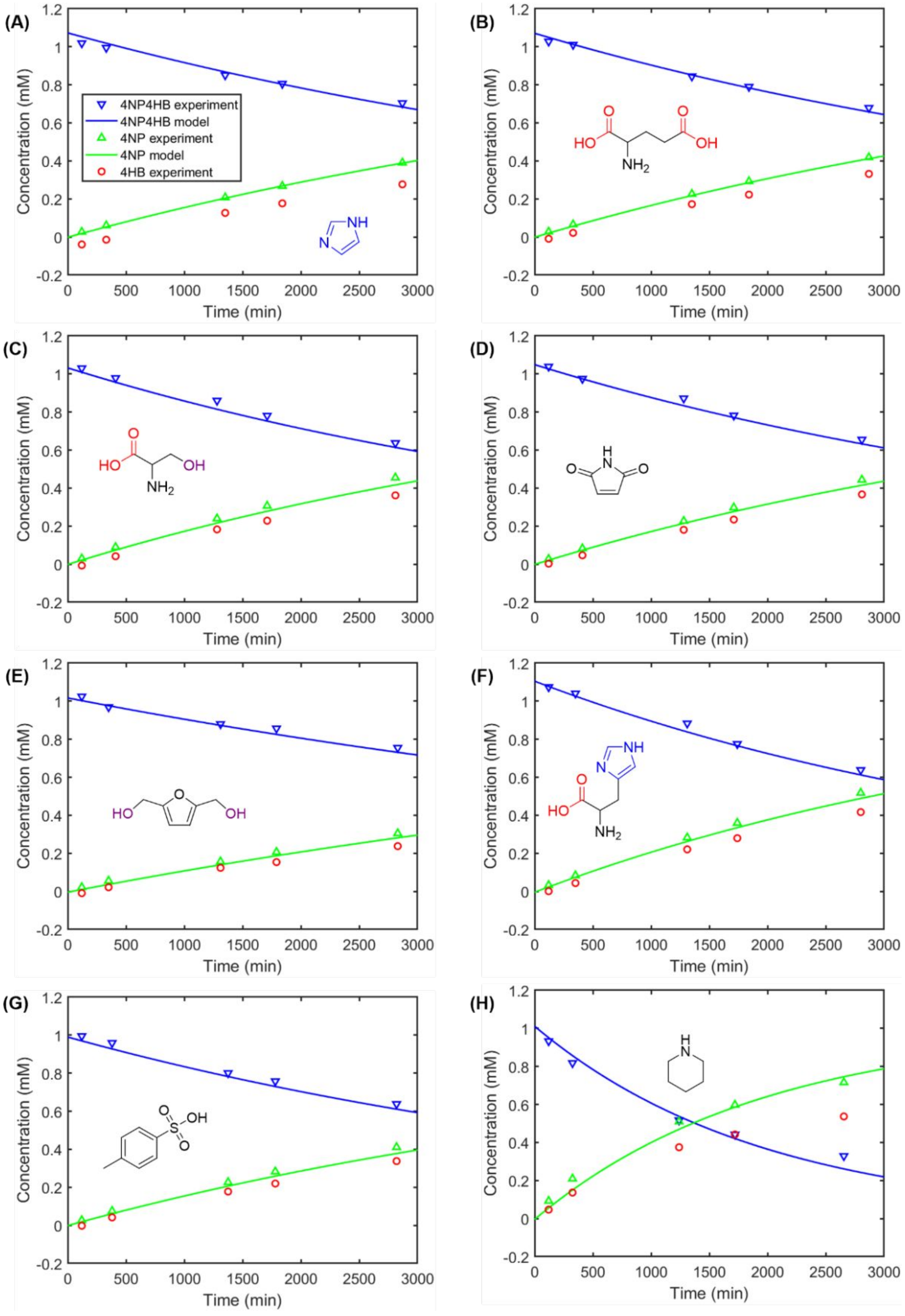

Figure S11. Experimental concentrations and kinetic model concentrations for reaction kinetics analysis of model ester hydrolysis at 50℃ in 50% acetonitrile and 50% borate buffer (pH 8.4) by volume with $[4NP4HB]_i$ of 1.0 mM and catalyst/feed of 0.5 catalyzed by precursors or controls; (**A**) imidazole, (**B**) glutamic acid, (**C**) serine, (**D**) maleimide, (**E**) FDM, (**F**) histidine, (**G**) *p*-toluenesulfonic acid, and (**H**) piperidine.

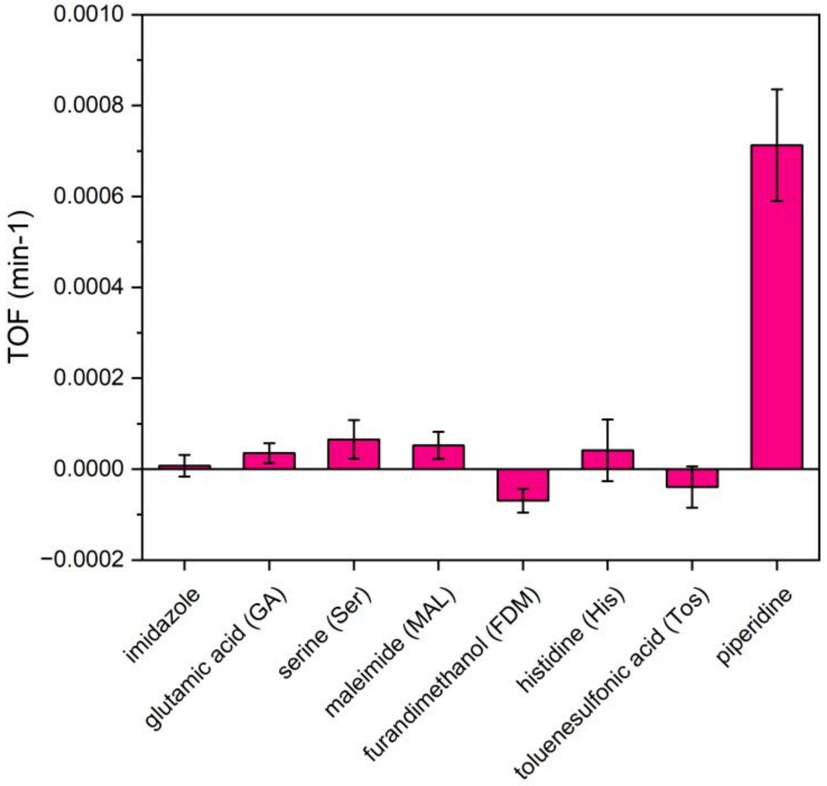

Figure S12. The intrinsic catalytic activity (TOFs) of precursors and controls for model ester hydrolysis at 50℃ in 50% acetonitrile and 50% borate buffer (pH 8.4) by volume with $[4NP4HB]_i$ of 1.0 mM and catalyst/feed of 0.5. Error was computed as the 95% confidence interval from nonlinear fitting.

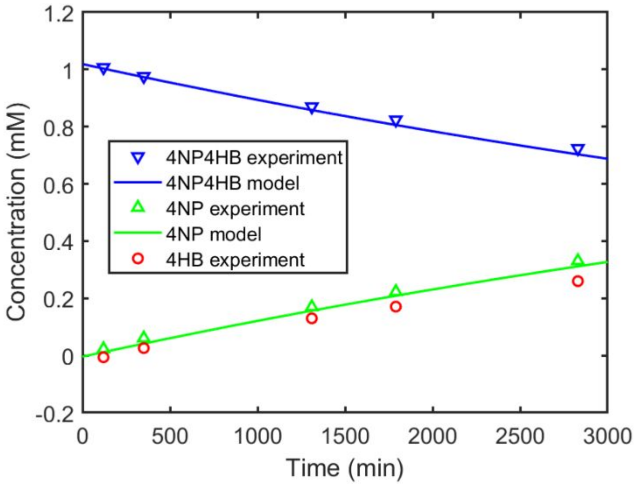

Figure S13. Experimental concentrations and kinetic model concentrations for reaction kinetics analysis of model ester hydrolysis at 50℃ in 50% acetonitrile and 50% borate buffer (pH 8.4) by volume with $[4NP4HB]_i$ of 1.0 mM and catalyst/feed of 0.5 catalyzed by a mixture of amino acids, HIS and SER, with the total catalyst concentration calculated as the sum of their individual concentrations.

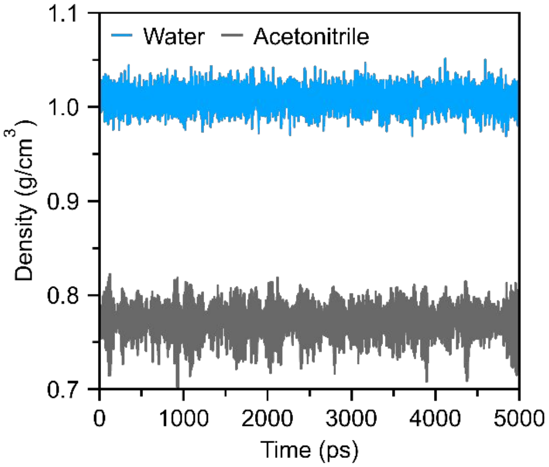

Figure S14. Density calculation for water and acetonitrile at 25°C.

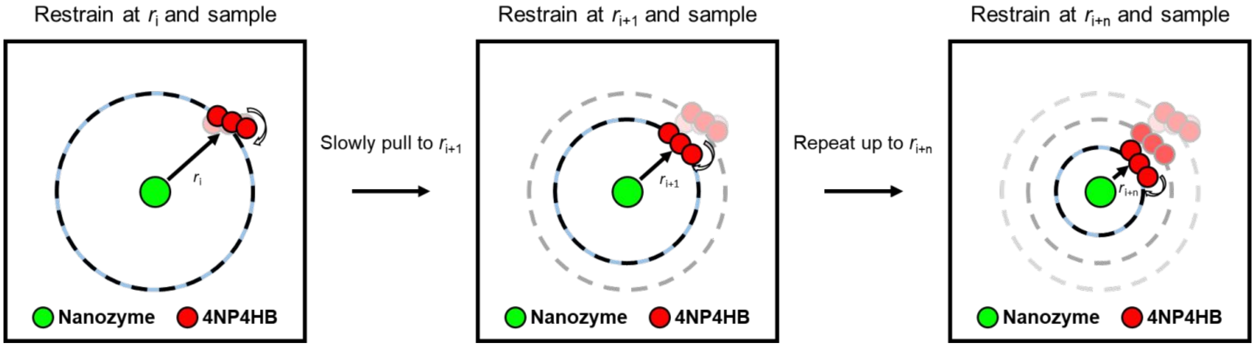

Figure S15. Schematic illustrating the methodology used to pull 4NP4HB near the nanozyme.

Table S3. Number of solvent molecules and counterions in all systems at different acetonitrile (ACN) concentrations. Because FDM-GA-MAL has an additional carboxylate, those systems had one water molecule and one additional sodium counterion.

Molecule	50% v/v ACN	25% v/v ACN
H_2O	2094	3143
ACN	706	353
Na^+	6	8
Cl^-	5	7

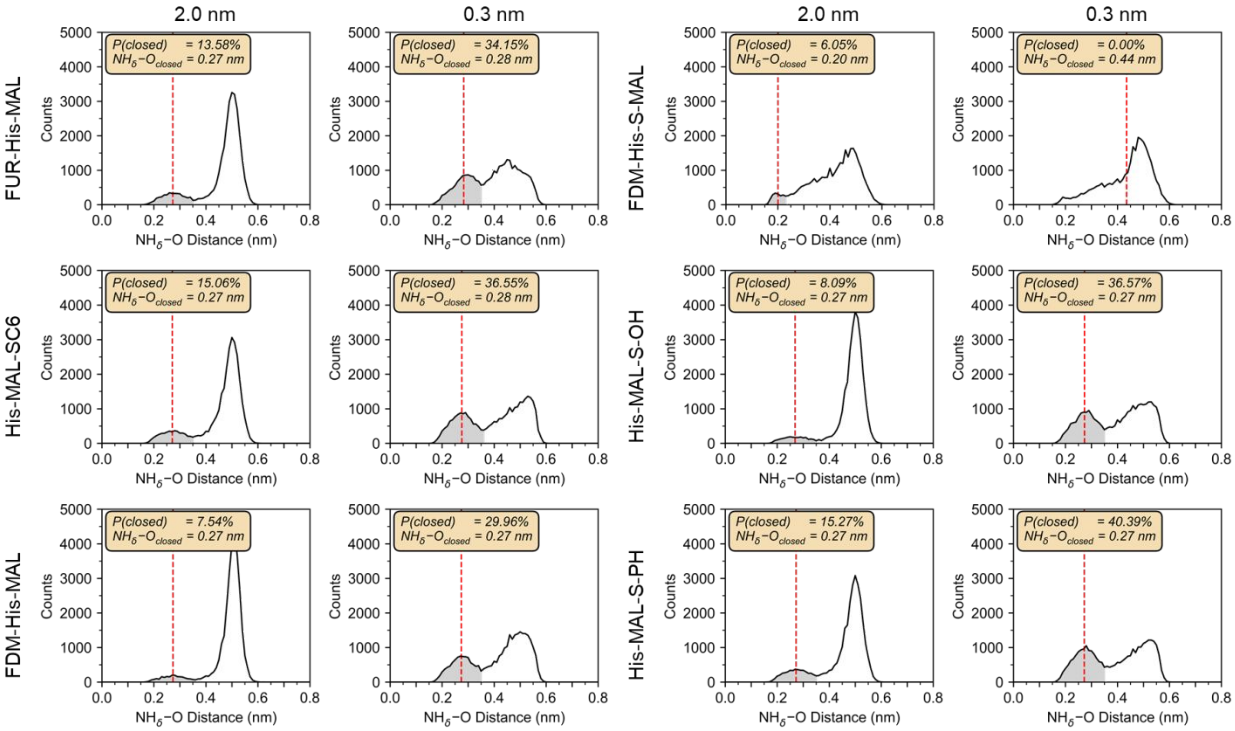

Figure S16. Probability distribution functions of the NH_{δ}–O distance when 4NP4HB is restrained either 2.0 nm or 0.3 nm from the nanozyme. The closed state probability is shaded grey. A red and dashed vertical line denotes the average NH_{δ}–O bond length of the closed state conformations. Data correspond to a single replicate of each nanozyme.

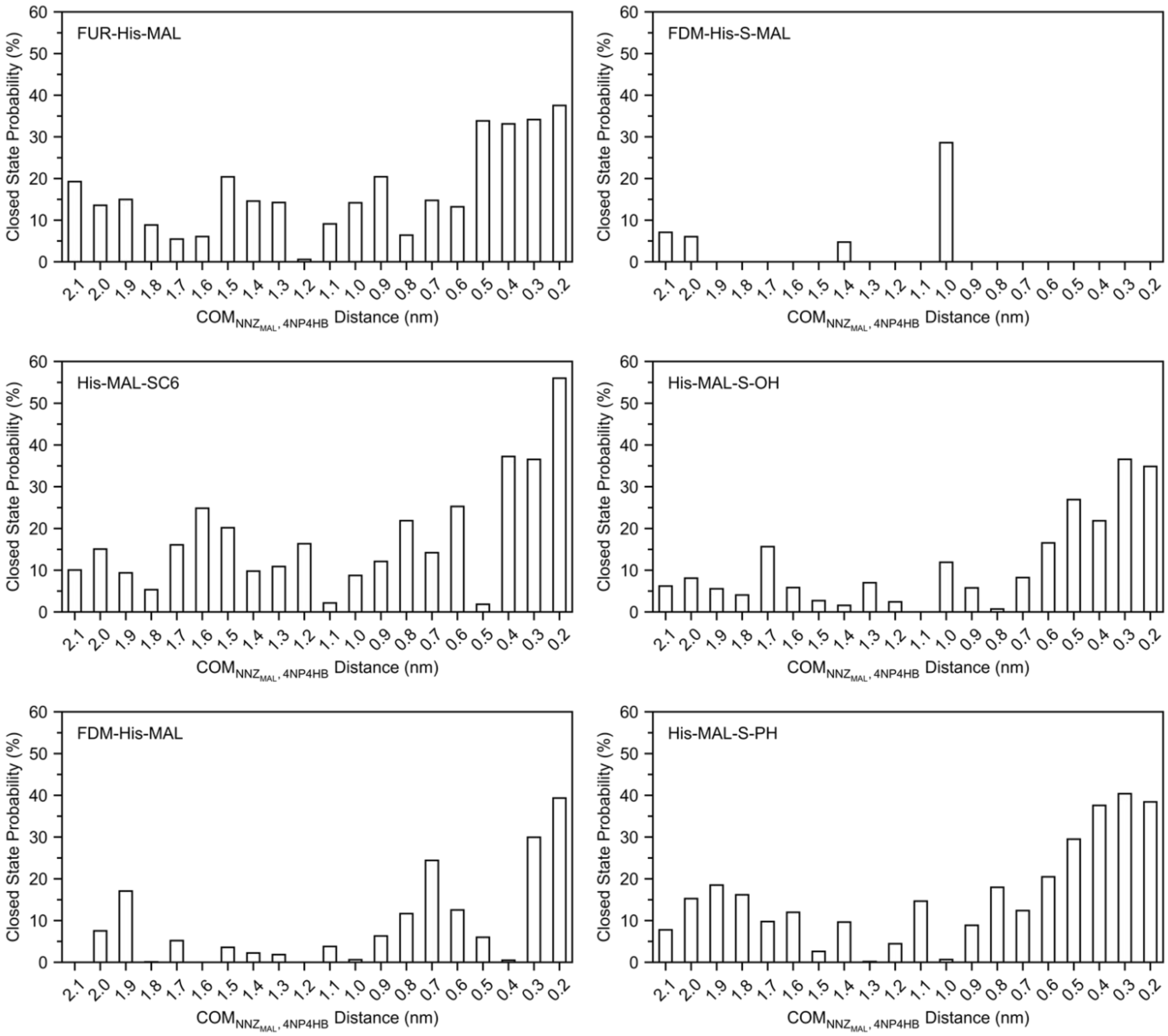

Figure S17. Closed state probability along the center-of-mass (COM) distance between the nanozyme's maleimide (NNZ_{MAL}) group and 4NP4HB. Data correspond to a single replicate of each nanozyme.

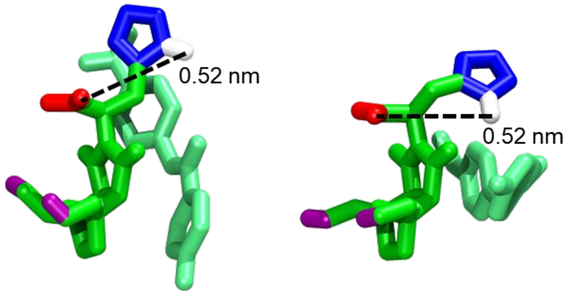

Figure S18. Simulation snapshots showing open state conformations for FDM-His-MAL when 4NP4HB is near the nanozyme. The functional groups on the nanozymes are colored as follows: red for carboxylate, blue for imidazole, and purple for hydroxyl; the rest of the molecule is colored green. 4NP4HB is colored lime. Only the hydrogen of the imidazole's N_δ is shown (colored white) for visual purposes.

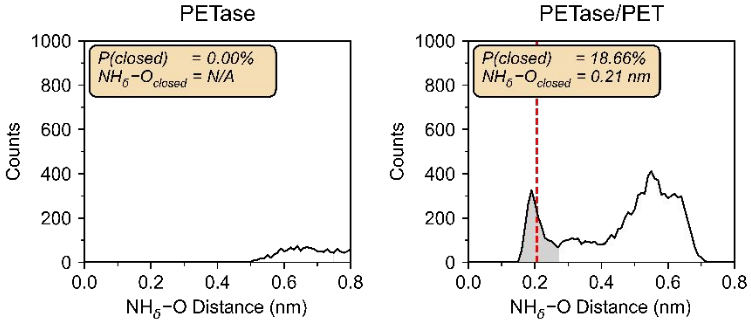

Figure S19. Probability distribution functions for the NH_δ–O bond length when PET is absent or present at the catalytic triad active site. A red and dashed vertical line denotes the average NH_δ–O bond length of the closed state conformations for the PETase/PET simulation.

Table S4. Average fluctuations (σ) in NH_δ–O distances from the closed state probabilities when 4NP4HB is near the nanozyme. Tabulated average NH_δ–O distances (μ) correspond to the data in Fig. 4B from the main text ("Near" labels). The standard deviation for the closed state probabilities of simulations with $COM_{NNZ_{MAL}-4NP4HB}$ of 0.3 nm was calculated for each replicate and averaged. Replicates with unimodal distributions that favored the open states were excluded.

Nanozyme	μ (Å)	σ (Å)
FUR-His-MAL	2.8	0.4
His-MAL-SC6	2.7	0.5
FDM-His-MAL	2.7	0.4
His-MAL-S-OH	2.8	0.5
His-MAL-S-PH	2.7	0.4
PETase/PET	2.1	0.3

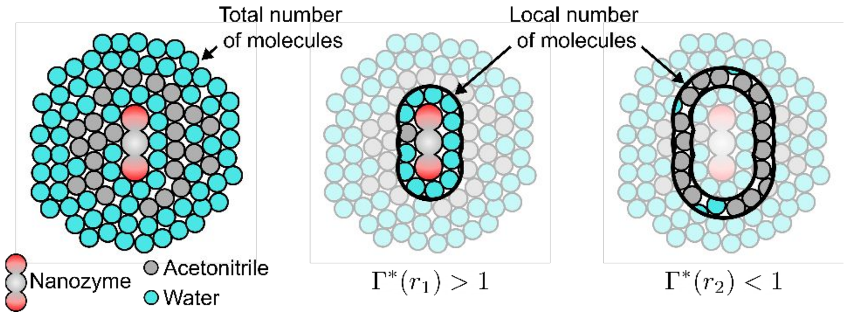

Figure S20. Schematic illustrating how preferential hydration is calculated with respect to the nanozyme's closest atoms. Only cosolvent molecules in a 3D shell at a distance *r* centered on each nanozyme atom are counted.

Table S5. Number of water (H_2O) and acetonitrile (ACN) molecules within the 0.3 nm, 0.4 nm, and 0.5 nm intervals at different concentrations of ACN.

Nanozyme	% v/v ACN	H_2O	ACN
His-MAL-SC6	50	41.17	16.65
	25	57.58	11.07
FDM-His-MAL	50	42.08	11.11
	25	56.01	7.25

Table S6. Summary of intrinsic catalytic activity of FDM-His-MAL, His-MAL-SC6, and piperidine for 4NP4HB hydrolysis at 50℃ in varied composition of acetonitrile and borate buffer (pH 8.4) solvent system. Error was computed as the 95% confidence interval from nonlinear fitting.

Catalyst	ACN (% v/v)	$[4NP4HB]_i$ (mM)	[cat] (mM)	k_{cat} ($min^{-1}\cdot M^{-1}$)	TOF (10^{-3} min^{-1})	TON
FDM-His-MAL	50*	0.993	0.498	11.82 ± 1.25	11.74 ± 1.24	4.23
	50	0.538	0.249	9.66 ± 0.98	5.20 ± 0.53	1.87
	25	0.498	0.249	25.47 ± 2.87	12.68 ± 1.43	4.57
	5	0.104	0.010	59.78 ± 9.19	6.22 ± 0.96	2.24
His-MAL-SC6	50*	1.020	0.495	-0.46 ± 0.51	-0.47 ± 0.52	-1.24
	50	0.549	0.247	-0.44 ± 0.44	-0.24 ± 0.24	-0.67
	25	0.508	0.247	-0.04 ± 0.51	-0.02 ± 0.26	-0.06
Piperidine	50*	1.011	0.435	0.70 ± 0.12	0.71 ± 0.12	1.89
	50	0.542	0.217	0.77 ± 0.12	0.42 ± 0.06	1.15
	25	0.506	0.217	0.49 ± 0.14	0.25 ± 0.07	0.68
	5	0.104	0.009	-3.33 ± 6.98	-0.35 ± 0.73	-0.99

*Results from Trial 1.

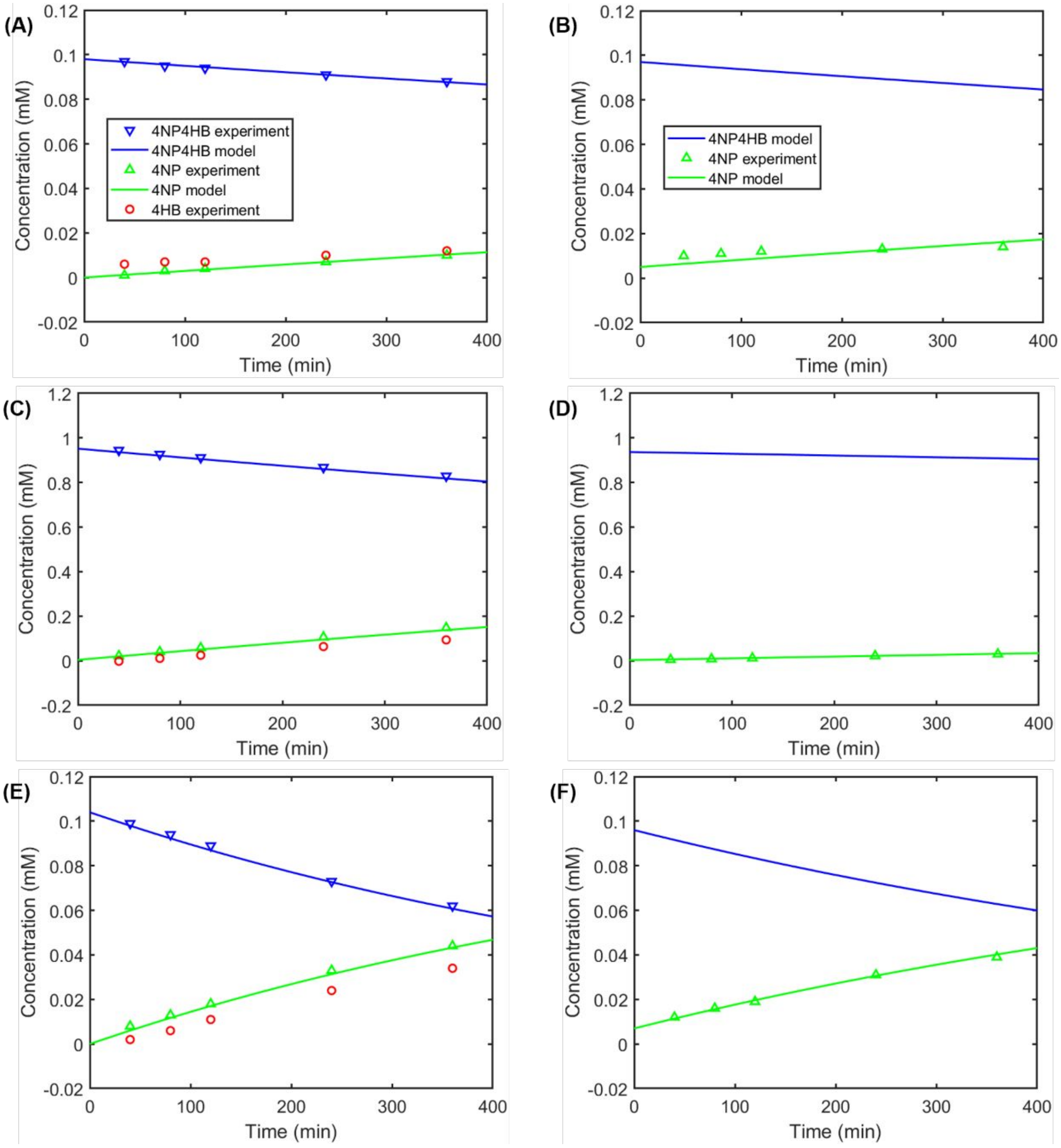

Figure S21. Experimental concentrations and kinetic model for reaction kinetics analysis of model ester hydrolysis at room temperature in 5% v/v acetonitrile solution with $[4NP4HB]_i$ of 0.1 mM and catalyst/feed of 0.1 catalyzed by (**A**) FDM-His-MAL and (**B**) α-Chymotrypsin; at room temperature in 50% v/v acetonitrile solution with $[4NP4HB]_i$ of 1.0 mM and catalyst/feed of 0.1 catalyzed by (**C**) FDM-His-MAL and (**D**) α-Chymotrypsin; 50℃ in 5% v/v acetonitrile solution with $[4NP4HB]_i$ of 0.1 mM and catalyst/feed of 0.1 catalyzed by (**E**) FDM-His-MAL and (**F**) α-Chymotrypsin.
